# Structural and Functional Insights Into (*S*)‐5′‐*C*‐Guanidinopropyl Uridine‐Modified Oligonucleotides With 2′‐*O*‐Methyl and 2′‐Fluoro Substitutions for RNAi Therapeutics

**DOI:** 10.1002/cmdc.70413

**Published:** 2026-08-07

**Authors:** Sugahara Masaki, Elsayed M. Mahmoud, Shuichi Sakamoto, Yoshihito Ueno

**Affiliations:** ^1^ United Graduate School of Agricultural Science Gifu University Gifu Japan; ^2^ Course of Applied Life Science Faculty of Applied Biological Sciences Gifu University Gifu Japan; ^3^ Department of Pharmaceutical Organic Chemistry Faculty of Pharmacy Zagazig University Zagazig Egypt; ^4^ Institute of Microbial Chemistry (BIKAKEN) Numazu Branch Microbial Chemistry Research Foundation Numazu Shizuoka Japan; ^5^ Department of Life Science and Chemistry The Graduate School of Natural Science and Technology Gifu University Gifu Japan; ^6^ Center for One Medicine Innovative Translational Research (COMIT) Tokai National Higher Education and Research System Gifu University Gifu Japan

**Keywords:** 2′‐fluoro, 2′‐*O*‐methyl, guanidinopropyl uridine, mismatch discrimination, nuclease resistance, oligonucleotides, RNA interference (RNAi), thermal denaturation

## Abstract

Guanidinium moieties are important structural motifs for improving the physicochemical and biological properties of oligonucleotides. Here, two novel uridine analogs, (*S*)‐5′‐*C*‐guanidinopropyl‐2′‐*O*‐methyluridine (5′‐Gp‐2′‐MoU, **2**) and (*S*)‐5′‐*C*‐guanidinopropyl‐2′‐fluorouridine (5′‐Gp‐2′‐FU, **3**), were synthesized and incorporated into RNA oligonucleotides. Thermal denaturation studies revealed that single incorporation of **2** slightly reduced duplex stability, whereas **3** produced modest stabilization; however, multiple incorporations markedly decreased melting temperatures, indicating cumulative destabilization. Thermodynamic analyses showed that this effect was primarily enthalpy‐driven and partially compensated by favorable entropy contributions. Circular dichroism spectroscopy confirmed preservation of the A‐form helical structure, although slight reductions in negative band intensity suggested localized conformational perturbations. siRNAs containing terminal or overhang modifications retained typical A‐form signatures without detectable spectral changes. Mismatch discrimination studies demonstrated enhanced sequence selectivity, particularly against U:U and U:G mismatches. Serum stability assays further revealed substantially improved nuclease resistance, with 5′‐Gp‐2′‐MoU (**2**) exhibiting the highest stability, comparable to the previously reported 5′‐Ap‐2′‐MoU (**1**). Moreover, *KNTC2*‐targeting siRNAs containing these modifications maintained potent gene‐silencing activity in HCT116 cells under lipofection conditions, demonstrating compatibility with RNA‐induced silencing complex (RISC) function. These findings identify 5′‐guanidinopropyl‐modified uridine analogs as promising nucleoside modifications for improving the stability and therapeutic potential of RNA‐based therapeutics.

## Graphical abstract

Novel (*S*)‐5′‐guanidinopropyl uridine analogs bearing 2′‐*O*‐methyl or 2′‐fluoro substitutions enhance nuclease resistance and mismatch discrimination while preserving A‐form RNA structure and potent RNAi activity. These modifications provide a promising strategy for improving the stability, specificity, and therapeutic potential of siRNA‐based medicines.

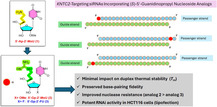

## 1 Introduction

In recent decades, nucleic acid‐based therapeutics have emerged as a transformative class of medicines, offering a novel approach to disease treatment distinct from traditional small‐molecule drugs and protein‐based biologics [1, 3]. These agents, which are composed of DNA or RNA sequences, function by modulating gene expression at the transcriptional or post‐transcriptional level, thereby enabling the precise control of cellular pathways [4, 5]. Small interfering RNAs (siRNAs) have garnered considerable attention owing to their ability to silence disease‐associated genes through RNA interference (RNAi), a naturally occurring and highly specific post‐transcriptional gene regulation mechanism [6, 7].

The therapeutic appeal of siRNAs lies in their unique mode of action. Unlike conventional drugs that target protein structures, siRNAs can be designed to bind virtually any mRNA sequence via complementary base pairing [8, 9]. This versatility significantly broadens the druggable genome, particularly for targets that are considered inaccessible to traditional therapeutic modalities [10]. Additionally, siRNAs exhibit high sequence specificity, are chemically synthesizable, and offer rapid adaptability, making them attractive candidates for the treatment of genetic disorders, cancer, and infectious diseases [11, 12].

Mechanistically, RNAi involves the incorporation of double‐stranded siRNAs into the RNA‐induced silencing complex (RISC), where the antisense (guide) strand directs the RISC to a complementary target mRNA. This interaction leads to endonucleolytic cleavage by the Argonaute 2 (Ago2) protein, resulting in the degradation of the target transcript and subsequent gene silencing [13, 14]. Although this sequence‐specific mechanism underpins the therapeutic potential of siRNAs, their clinical application is hampered by several structural and functional limitations [15].

One of the most critical challenges is the poor cellular uptake of siRNAs owing to their polyanionic nature, which causes electrostatic repulsion from negatively charged cell membranes [16, 17]. Furthermore, siRNAs are inherently unstable in biological environments and highly vulnerable to rapid degradation by nucleases present in both extracellular fluids and intracellular compartments [15]. These factors significantly reduce the circulation half‐life and therapeutic efficacy [18, 19]. Additionally, siRNAs can display off‐target effects by partially hybridizing with unintended mRNA sequences, leading to nonspecific gene silencing and adverse biological consequences [20, 21].

To overcome these obstacles, extensive studies have focused on chemical modifications that enhance the pharmacological profile of siRNAs without compromising their gene‐silencing capabilities [22, 23]. One of the most widely adopted approaches involves modification of the ribose sugar, particularly at the 2′‐position. Substituents such as 2′‐*O*‐methyl (2′‐OMe) and 2′‐fluoro (2′‐F) not only confer resistance to nuclease degradation but also promote puckering of the *N*‐type (C3′‐*endo*) sugar, which is essential for maintaining the A‐form helical geometry required for efficient RISC loading [24, 25]. These modifications further contribute to improved selectivity by minimizing off‐target interactions and nonspecific incorporation into the RNAi machinery.

Our laboratory previously investigated structural modifications at less commonly explored positions of the ribose, specifically the 4′ and 5′ carbons [26, 29]. We introduced aminoalkyl side chains at these sites to evaluate their potential to enhance nuclease resistance and cellular uptake. The rationale behind this design is the proximity of these positions to the internucleoside phosphodiester linkage, the primary target of nuclease attack [30]. The incorporation of bulky side chains at these sites provides steric hindrance against enzymatic degradation, whereas the introduction of positively charged amino groups under physiological conditions improves duplex stability through electrostatic shielding of the phosphate backbone [31]. Moreover, these modifications may enhance membrane interactions and uptake by leveraging electrostatic and hydrogen bonding interactions [31]. As a representative example, the 5′‐*C*‐aminopropyl modification, particularly in the (*S*)‐configuration, has emerged as a highly effective strategy for enhancing siRNA performance [32, 35]. This modification improves the thermal stability of dsRNA and siRNA and confers superior serum stability compared with the 4′‐*C*‐aminopropyl and (*R*)‐5′‐*C*‐aminopropyl analogs [35]. Moreover, 5′‐*C*‐aminopropyl‐modified siRNAs incorporating 2′‐*O*‐methyl and 2′‐fluoro substitutions exhibit a balanced profile of enhanced stability, retained RNAi activity, and improved cellular internalization, underscoring the therapeutic potential of this modification [32, 35].

Despite these promising results, further optimization is necessary to fully address the challenges associated with siRNA therapeutics. Among the various structural modifications under investigation, guanidino groups have been recognized as promising alternatives owing to their distinctive and desirable physicochemical properties. These moieties are characterized by a resonance‐stabilized delocalized positive charge distributed over three nitrogen atoms, which imparts strong basicity and robust hydrogen‐bonding capabilities [36]. Compared with conventional amino groups, guanidine functionalities offer a greater density of positive charges, which effectively counteracts the inherent anionic nature of oligonucleotides (ONs) [37, 38]. This enhanced cationic character not only strengthens electrostatic interactions with nucleic acids but also protects against nuclease‐mediated degradation [39]. Furthermore, guanidino groups enhance cellular uptake by mimicking the membrane‐permeabilizing function of arginine residues commonly found in arginine‐rich cell‐penetrating peptides. Accordingly, guanidino modifications have been introduced at various sites within ONs, including the 2′ [40] and 4′ [41] ribose positions, nucleobases [42, 43], and interphosphate linkages [36], to enhance their therapeutic performance.

Building on these insights, we hypothesized that replacing the terminal amino group of the (*S*)‐5′‐*C*‐aminopropyl side chain with a bulkier guanidino moiety would yield a structurally optimized nucleoside analog with superior performance. Such a modification is anticipated to retain or enhance nuclease resistance and cellular uptake, while further improving target recognition and duplex stability owing to the multifunctional properties of the guanidine group.

**FIGURE 1:**
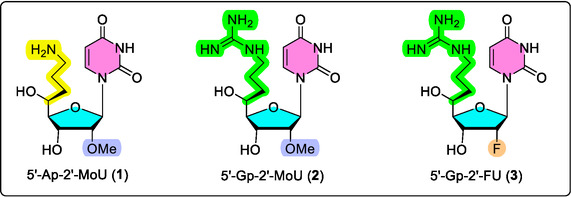
Chemical structures of the modified uridine derivatives investigated in this study. Compound 5′‐Ap‐2′‐MoU (**1**), which was previously characterized, serves as a reference for the comparative evaluation of the biochemical properties of the newly synthesized derivatives, 5′‐Gp‐2′‐MoU (**2**) and 5′‐Gp‐2′‐FU (**3**).

In this work, we describe the design, synthesis, and comprehensive characterization of a novel class of ON analogs bearing an (*S*)‐5′‐*C*‐guanidinopropyl side chain in combination with established 2′ modifications, namely, 2′‐*O*‐methyl and 2′‐fluoro substitutions (denoted as analogs **2** and **3**; Figure 1). We present a synthetic strategy that enables the stereochemically pure introduction of a guanidopropyl functionality at the 5′ position of uridine derivatives and systematically evaluate its impact on key biophysical and biochemical properties, including duplex thermodynamic stability, mismatch discrimination, and conformational integrity. Nuclease resistance was further examined through comparative analyses with unmodified dsRNA and a previously reported (*S*)‐5′‐*C*‐aminopropyl‐modified analog (analog **1**; Figure 1). Finally, *KNTC2*‐targeting siRNAs incorporating these modifications were designed and evaluated for their gene‐silencing efficacy in HCT116 human colon cancer cells under lipofection conditions, thereby validating their potential utility in RNAi‐based therapeutic applications. This study introduces a new structural paradigm for RNAi therapeutics that enhances delivery, stability, and specificity, while maintaining effective gene silencing, thereby advancing siRNA design for gene therapy and precision medicine.

## 2 Results and Discussion

### 2.1 Synthesis of Nucleoside Analogs

**SCHEME 1:**
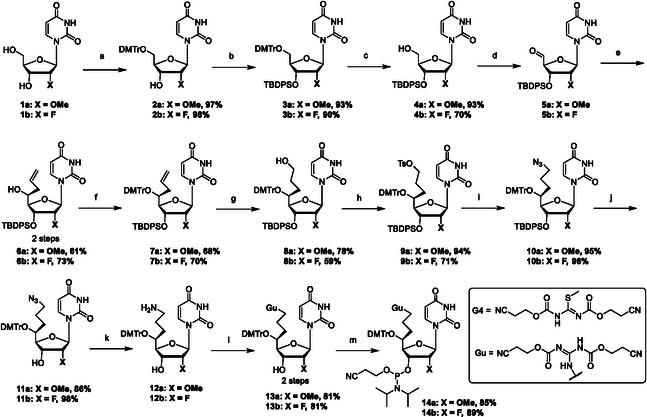
Synthesis of phosphoramidites **14a** and **14b** of uridine analogs 5′‐Gp‐2′‐MoU (**2**) and 5′‐Gp‐2′‐FU (**3**)^a^. ^a^Reagents and conditions: (a) DMTrCl, pyridine, r.t., 21 h; (b) TBDPSCl, imidazole, DMF, r.t.,19 h; (c) TFA:MeOH:DCM (=1:5:14), r.t., 5 h; (d) EDC·HCl, CHCl_2_CO_2_H, DMSO/CH_2_Cl_2_ (1:1; v/v), 0 °C, 2 h; (e) allyltrimethylsilane, BF_3_·OEt_2_, CH_2_Cl_2_, 0 °C, 2 h; (f) DMTrCl, AgNO_3_, THF/pyridine, r.t., 22 h; (g) 9‐BBN, THF, r.t., 24 h then 30% H_2_O_2_ aq., 2N NaOH aq., r.t., 3 h; (h) *p*‐TsCl, pyridine, CH_2_Cl_2_, −20 °C, 19 h; (i) NaN_3_, DMF, 50 °C, 20 h; (j) TBAF/THF, THF, r. t., 3 h; (k) PPh_3_, H_2_O, THF, 40 °C, 6 h; (l) G4 (structure shown in Scheme 1), pyridine, CH_2_Cl_2_, 45 °C, 2 h; (m) 2‐cyanoethyl *N*,*N*‐diisopropylchlorophosphoramidite, *N*,*N*‐diisopropylethylamine, THF, r.t., 3 h.

Scheme 1 illustrates the synthetic pathway for the preparation of monomeric 5‐guanidinopropyl‐modified nucleoside analogs, culminating in the formation of phosphoramidite derivatives **14a** and **14b**. The synthesis commenced from commercially available 2′‐*O*‐methyluridine and 2′‐fluorouridine, which were transformed into the key azidopropyl intermediates **10a** and **10b** through a nine‐step sequence, following previously reported protocols [32, 35]. The synthetic route involved initial selective protection of the 5′‐hydroxyl group using a dimethoxytrityl (DMTr) group, followed by silylation at the 2′‐position. Subsequent removal of the DMTr group regenerated the primary 5′‐hydroxyl functionality, which was then subjected to oxidation to afford the corresponding aldehyde intermediates **5a** and **5b**. Without further purification, the crude aldehydes underwent allylation with allyltrimethylsilane in the presence of boron trifluoride diethyl etherate (BF_3_·OEt_2_) as a Lewis acid, yielding the corresponding 5′‐*C*‐allyluridine derivatives. These intermediates were then subjected to hydroboration–oxidation to furnish the 5′‐*C*‐hydroxypropyluridine derivatives **8a** and **8b**. Subsequent tosylation of the hydroxyl group, followed by nucleophilic substitution with azide, afforded the key azidopropyl intermediates **10a** and **10b**, as previously reported by our group [32, 35]. Selective removal of the *tert*‐butyldiphenylsilyl (TBDPS) protecting group was then achieved using tetrabutylammonium fluoride (TBAF) in tetrahydrofuran (THF), affording compounds **11a** and **11b** in excellent yields of 86% and 98%, respectively. The subsequent reduction of the azide groups via the Staudinger reaction using triphenylphosphine (PPh_3_) and distilled water in THF yielded the corresponding amino derivatives **12a** and **12b**. These crude amines were used directly in the next step without further purification. Introduction of the guanidino functionality at the 5′‐position was achieved by reacting the amines with the guanidinating reagent G4 [*N*,*N*′‐bis(2‐cyanoethoxycarbonyl)‐*S*‐methylisothiourea], synthesized following established procedures [44, 45].

This two‐step conversion furnished the guanidino‐substituted intermediates **13a** and **13b** in 81% overall yield. Finally, phosphorylation at the 3′‐hydroxyl position was accomplished using 2‐cyanoethyl *N*,*N*‐diisopropylchlorophosphoramidite in the presence of *N*,*N*‐diisopropylethylamine as the base, affording the target phosphoramidite derivatives **14a** and **14b** in 85% and 89% yields, respectively. These final products are compatible with automated solid‐phase ON synthesis. The complete synthetic route comprised 13 steps, resulting in overall isolated yields of 15% for **14a** and 9% for **14b**. The ^31^P NMR spectrum of compound **14b** displayed signals corresponding to partially oxidized P(V) species. Given that phosphoramidites are extremely sensitive to trace amounts of oxygen and moisture, some degree of oxidation probably occurred during handling, storage, or the preparation of the NMR sample following isolation. Nevertheless, compound **14b** exhibited coupling efficiency comparable to that of standard RNA phosphoramidites during automated RNA synthesis under routine anhydrous conditions.

Before incorporating the modified nucleosides into RNA oligomers, their sugar‐puckering conformations were systematically evaluated. The relative populations of the *North* (C3′‐*endo*) conformation in nucleoside analogs **2** and **3** were estimated based on the vicinal proton–proton coupling constant (*J*_1′−2′_) obtained via nuclear magnetic resonance (NMR) spectroscopy [46, 48]. The percentage of the *North* conformer was calculated using the equation: *North* (%) = [100 − *J*_1′−2′_ × 10], and the results were compared to those of natural uridine. The calculated *North* conformer populations for analogs **2** and **3** were 52% and 70%, respectively, whereas the natural uridine exhibited a lower value of 45%. These results indicate that the structural modifications in analogs **2** and **3** shift the sugar conformational equilibrium toward the C3′‐*endo* pucker relative to the unmodified nucleoside. Moreover, analog **3** displayed a higher propensity for the *N*‐type conformation than analog **2**, likely owing to the enhanced anomeric effect associated with the increased electronegativity of the fluorine substituent. These conformational shifts are anticipated to contribute to improved binding affinity for RNA/RNA duplex formation.

ONs incorporating analogs **1**, **2**, and **3** were successfully synthesized on an automated DNA/RNA synthesizer using standard solid‐phase phosphoramidite chemistry. Following cleavage from the solid support and deprotection, the entire crude product from each synthesis was purified by 20% denaturing polyacrylamide gel electrophoresis (PAGE). The identity of the purified ONs was confirmed by MALDI–TOF mass spectrometry, and their purity was further assessed by HPLC analysis (Figures S1 and S2). The sequences of the synthesized– ONs are listed in  Tables 1–5.

**TABLE 1:** | Sequences of ssRNAs, dsRNAs, and *T*_m_ values of dsRNAs.

Abbreviation of dsRNA	Abbreviation of ssRNAs	Sequence^a^	*T*_m_, °C^b^	Δ*T*_m_, °C^c^
dsRNA **1**	RNA **1**	UUC UUC UUC UU	42.3 (±0.6)	—
	RNA **2**	AAG AAG AAG AA		
dsRNA **2**	RNA **3**	UUC U**u**C UUC UU	43.9 (±0.3)	+ 1.6
	RNA **2**	AAG AAG AAG AA		
dsRNA **3**	RNA **4**	UUC U **u** C UUC UU	44.2 (±0.1)	+ 1.9
	RNA **2**	AAG AAG AAG AA		
dsRNA **4**	RNA **5**	UUC U **2** C UUC UU	42.0 (±0.4)	−0.3
	RNA **2**	AAG AAG AAG AA		
dsRNA **5**	RNA **6**	UUC U **3** C UUC UU	42.5 (±0.3)	+ 0.2
	RNA **2**	AAG AAG AAG AA		
dsRNA **6**	RNA **7**	U **2** C UUC **2** UC **2** U	40.3 (±0.3)	−2.0
	RNA **2**	AAG AAG AAG AA		
dsRNA **7**	RNA **8**	U **3** C UUC **3** UC **3** U	40.3 (±0.2)	−2.0
	RNA **2**	AAG AAG AAG AA		

a **u**, **u** , **2** **( **red** )**, and **3** **( **blue** )** denote 2′‐MoU, 2′‐FU, 5′‐Gp‐2′‐MoU (analog **2**), and 5′‐Gp‐2′‐FU (analog **3**), respectively.

b The *T*_m_s was measured in a buffer containing 10 mM sodium phosphate (pH 7.0) and 100 mM NaCl. The duplex concentration was 3 µM. All experiments were carried out in triplicate, and data are presented as the mean ± SD.

c Δ*T*_m_ = [*T*_m_ (dsRNA_mod_) − *T*_m_ (dsRNA **1**)].

**TABLE 2:** | Sequences of ssRNAs, siRNAs, and *T*_m_ values of siRNAs.

Abbreviation of siRNA	Abbreviation of ssRNAs	Sequence^a^	*T*_m_, °C^b^	Δ*T*_m_, °C^c^
siRNA **1**	RNA **9**	5′‐uag uca AcU UGg uau auu uuu‐3′	77.1 (±0.1)	—
	RNA **10**	3′‐uua ucA gUu gaa CCa Uau aAa‐5′		
siRNA **2**	RNA **11**	5′‐ **2** ag uca AcU UGg uau auu uuu‐3′	76.6 (±0.1)	−0.5
	RNA **10**	3′‐uua ucA gUu gaa CCa Uau aAa‐5′		
siRNA **3**	RNA **12**	5′‐uag uca AcU UGg uau auu u **2** u‐3′	76.4 (±0.2)	−0.7
	RNA **10**	3′‐uua ucA gUu gaa CCa Uau aAa‐5′		
siRNA **4**	RNA **9**	5′‐uag uca AcU UGg uau auu uuu‐3′	76.2 (±0.1)	−0.9
	RNA **13**	3′‐u **2** a ucA gUu gaa CCa Uau aAa‐5′		
siRNA **5**	RNA **14**	5′‐ **3** ag uca AcU UGg uau auu uuu‐3′	76.3 (±0.1)	−0.8
	RNA **10**	3′‐uua ucA gUu gaa CCa Uau aAa‐5′		
siRNA **6**	RNA **15**	5′‐uag uca AcU UGg uau auu u **3** u‐3′	76.4 (±0.2)	−0.7
	RNA **10**	3′‐uua ucA gUu gaa CCa Uau aAa‐5′		
siRNA **7**	RNA **9**	5′‐uag uca AcU UGg uau auu uuu‐3′	77.1 (±0.1)	−
	RNA **16**	3′‐u **3** a ucA gUu gaa CCa Uau aAa‐5′		

a Small letter, capital letter, **2** **( **red** )**, and **3** **( **blue** )** indicate 2′‐*O*‐methyl (2′‐OMe), 2′‐fluoro (2′‐F), 5′‐Gp‐2′‐MoU (analog **2**), and 5′‐Gp‐2′‐FU (analog **3**), respectively.

b The *T*_m_s was measured in a buffer containing 10 mM sodium phosphate (pH 7.0) and 100 mM NaCl. The duplex concentration was 3 µM. All experiments were carried out three times, and data are presented as the mean ± SD.

c Δ*T*_m_ = [*T*_m_ (siRNA_mod_) − *T*_m_ (siRNA **1**)].

**TABLE 3:** | Thermodynamic parameters of dsRNAs.^a^

Abbreviation of dsRNA	Abbreviation of ssRNAs	Sequence^b^	Δ*H*° (kcal/mol)	Δ*S*° (cal/mol·K)	Δ*G*°_37 °C_ (kcal/mol)
dsRNA **1**	RNA **1**	UUC UUC UUC UU	−81.7	−230.7	−10.1
	RNA **2**	AAG AAG AAG AA			
dsRNA **6**	RNA **7**	U **2** C UUC **2** UC **2** U	−68.5	−192.0	−8.9
	RNA **2**	AAG AAG AAG AA			
dsRNA **7**	RNA **8**	U **3** C UUC **3** UC **3** U	−68.5	−191.5	−9.1
	RNA **2**	AAG AAG AAG AA			

a Thermodynamic parameters were determined from the plot of 1/Tm versus CT/4.

b **( **red** )**, and **3** **(** **blue** **)** denote 5′‐Gp‐2′‐MoU and 5′‐Gp‐2′‐FU, respectively.

**TABLE 4:** Summary of *T*_m_ values of fully matched aThermodynamic parameters of dsRNAsnd one‐mismatched dsRNAs.

*T*_m_, °C^c^ (Δ*T*_m_, °C)^d^		**X** b			
		a	u	g	c
a **U**	U	42.3 (−)	30.9 (−11.4)	40.0 (−2.3)	28.9 (−13.4)
	analog **2**	42.0 (−)	28.7 (−13.3)	36.1 (−5.9)	30.7 (−11.3)
	analog **3**	42.5 (−)	28.5 (−14.0)	37.7 (−4.8)	30.1 (−12.4)

a The RNA strand sequence used in this study is 5′‐ UUC U **U** C UUC UU ‐3,′ where “U” and “C” denote native uridine and cytidine, respectively. Additionally, “ **U** ” may also represent the uridine analogs 5′‐Gp‐2′‐MoU (**2**) or 5′‐Gp‐2′‐FU (**3**).

b The complementary RNA strand follows the sequence 3′‐ AAG A **X** G AAG AA ‐5,′ where “a” and “g” correspond to native adenosine and guanosine, respectively. The letter “ **X** ” indicates the presence of a mismatched site.

c The melting temperature (*T*_m_) measurements were conducted in a buffer solution containing 10 mM sodium phosphate (pH 7.0) and 100 mM NaCl, with a duplex concentration of 3 µM. All experiments were performed in triplicate, and the reported values represent the calculated averages.

d The Δ*T*_m_ value reflects the difference between the melting temperatures of the mismatched and fully matched duplexes. This was determined using the formula [*T*_m_ (mismatched) – *T*_m_ (fully matched)].

### 2.2 Thermal Stability of Duplexes

A thermal denaturation study was conducted to evaluate the thermodynamic stability of unmodified and chemically modified RNA duplexes, with a focus on assessing the individual and combined effects of the uridine analog modifications. The investigated modifications included the incorporation of 2′‐*O*‐methyluridine (2′‐MoU) and 2′‐fluorouridine (2′‐FU), which were employed as reference analogs, as well as (*S*)‐*C*‐5′‐guanidinopropyl‐2′‐*O*‐methyluridine (analog **2**) and (*S*)‐*C*‐5′‐guanidinopropyl‐2′‐fluorouridine (analog **3**), designed to probe the influence of a 5′‐guanidinopropyl substituent. UV melting profiles (Figure S3) were recorded under standard physiological buffer conditions (10 mM sodium phosphate, pH 7.0, and 100 mM NaCl) at a duplex concentration of 3 µM.

The unmodified RNA duplex (dsRNA **1**) exhibited a melting temperature (*T*_m_) of 42.3 °C, serving as a reference for subsequent comparisons. To determine the effect of the 5′‐guanidinopropyl group on thermal stability, a single substitution with 2′‐MoU or 2′‐FU was introduced at the central position of the duplex (dsRNA **2** and dsRNA **3**, respectively). These modifications resulted in moderate increases in thermal stability, with *T*_m_ values of 43.9 °C (Δ*T*_m_ = +1.6 °C) for 2′‐MoU and 44.2 °C (Δ*T*_m_ = +1.9 °C) for 2′‐FU. These results are consistent with previous reports indicating that 2′‐*O*‐methyl and 2′‐fluoro sugar modifications enhance base stacking and/or backbone rigidity in RNA duplexes [49, 50].

By contrast, the incorporation of a single guanidinopropyl‐substituted analog, analog **2** (dsRNA **4**), or analog **3** (dsRNA **5**) led to negligible changes in duplex stability. The observed *T*_m_ values were 42.0 °C (Δ*T*_m_ = –0.3 °C) and 42.5 °C (Δ*T*_m_ = +0.2 °C), respectively. These findings suggest that the addition of the 5′‐guanidinopropyl moiety to a 2′‐MoU residue (analog **2**) does not significantly disrupt duplex formation. Furthermore, its combination with a 2′‐fluoro group (analog **3**) results in a slight stabilizing effect compared with the unmodified RNA. However, when compared directly with the duplexes containing only the 2′‐MoU or 2′‐FU modifications (dsRNA **2** and dsRNA **3**), the incorporation of the guanidinopropyl group in analogs **2** and **3** appears to slightly reduce duplex stability. These observations indicate that the incorporation of a guanidinopropyl moiety slightly diminished the stabilizing effects conferred by the 2′‐*O*‐methyl (2′‐OMe) or 2′‐fluoro modifications. However, when these modifications were combined, the overall impact on duplex stability was either mildly destabilizing or marginally stabilizing relative to the native duplex. Despite its modest effect on structural stability, the inclusion of the guanidinopropyl moiety offers significant functional advantages, particularly in enhancing cellular uptake and increasing membrane permeability.

To examine the effect of modification density, three modified nucleosides derived from analogs **2** and **3** were incorporated into the RNA strand, resulting in the formation of duplexes dsRNA **6** and dsRNA **7**. Both modified duplexes exhibited reduced thermal stability, with identical *T*_m_ values of 40.3 °C (Δ*T*_m_ = –2.0 °C). This pronounced destabilization likely arose from cumulative steric effects, disruption of the hydration shells, and perturbations of the backbone conformation introduced by the bulkier guanidinopropyl moieties.

Overall, these results highlight the nuanced impact of uridine modifications on RNA duplex stability. Single substitutions with 2′‐*O*‐methyl or 2′‐fluoro groups provide modest stabilization, while their guanidinopropyl derivatives induce either minimal destabilization (analog **2**) or slight stabilization (analog **3**) relative to the unmodified duplex. However, increasing the frequency of guanidinopropyl‐substituted analogs leads to measurable destabilization, emphasizing the need for a strategic balance between the type and density of modifications to achieve the desired structural and thermodynamic properties in RNA‐based applications.

To examine the effect of 5′‐guanidinopropyl (Gp) nucleoside analogs on siRNA duplex stability, a series of *KNTC2*‐targeting siRNAs containing site‐specific 5′‐Gp‐2′‐MoU (analog **2**) or 5′‐Gp‐2′‐FU (analog **3**) substitutions were designed (Table 2). The modifications were positioned at the 5′ end and the 3′ overhang of the passenger strand, as well as at the 3′ overhang of the guide strand, enabling systematic evaluation of both chemical and positional effects. The thermal stability of the duplex was assessed by UV melting analysis under physiologically relevant ionic conditions.

All the modified siRNAs exhibited high melting temperatures, indicating efficient duplex formation and overall structural integrity. Relative to the unmodified siRNA **1** (*T*_m_ = 77.1 °C), siRNAs **2**–**6** displayed only modest reductions in *T*_m_, with Δ*T*_m_ values ranging from −0.5 to −0.9 °C. These data demonstrate that a single incorporation of analog **2** or **3** is generally well tolerated within the siRNA duplex and does not substantially disrupt Watson–Crick base pairing.

Notably, the magnitude of thermal destabilization depended on both the nature of the 2′‐substitution and the location of the modification. Among the modified constructs, siRNA **4**, which incorporates a 5′‐Gp‐2′‐MoU analog at the 3′ overhang of the guide strand, exhibited the lowest thermal stability (Δ*T*_m_ = −0.9 °C). The 3′ overhang of the guide strand contributes to terminal stacking interactions that stabilize the duplex ends; introduction of the sterically demanding 2′‐OMe group in conjunction with the positively charged guanidinopropyl side chain likely perturbs local backbone geometry and weakens end‐stacking interactions. This combination may increase terminal fraying, resulting in a measurable decrease in the melting temperature.

By contrast, siRNA **7**, bearing a 5′‐Gp‐2′‐FU analog at the same guide‐strand 3′ overhang position, displayed a melting temperature identical to that of the unmodified duplex. The enhanced stability conferred by the 2′‐fluoro substituent is consistent with its known preference for a *C*3′‐*endo* sugar pucker and strengthens base‐stacking interactions. In this context, the compact size and conformational preorganization induced by the 2′‐F group likely compensate for any destabilizing effects introduced by the guanidinopropyl moiety, thereby preserving duplex stability.

Taken together, these results reveal that the thermal behavior of 5′‐Gp‐modified siRNAs is governed by a delicate interplay between modification chemistry and positional context. While 2′‐OMe‐containing analogs can induce mild destabilization when placed at structurally sensitive terminal regions, 2′‐F analogs effectively maintain duplex stability, even at the guide‐strand 3′ overhang. Importantly, the minimal changes in *T*_m_ across the siRNA panel confirm that 5′‐guanidinopropyl analogs can be incorporated without compromising duplex formation, supporting their further evaluation in RNAi functional studies.

### 2.3 Thermodynamic Analysis of Modified and Unmodified dsRNAs

To evaluate the thermodynamic impact of the 5′‐guanidinopropyl‐modified analogs on RNA duplex stability, we measured the melting temperatures (*T*_m_) of dsRNA **1** (unmodified), dsRNA **6**, and dsRNA **7** (each incorporating the modified uridine analogs) at varying ON concentrations (Figure S4). The data were plotted according to the van’t Hoff equation using the relationship between 1/*T*_m_ and ln(*C*_T_/4), where *C*_T_ represents the total strand concentration (Figure S5). From the linear fit of the plots, the enthalpy (Δ*H*°) and entropy (Δ*S*°) changes were determined from the slope and intercept, respectively, and the Gibbs free energy change (Δ*G*°) at 37 °C was subsequently calculated (Table 3).

The unmodified duplex (dsRNA **1**) displayed a thermodynamic profile, with Δ*H*° = −81.7 kcal/mol and Δ*S*° = −230.7 cal/mol·K, resulting in a Δ*G*° (37 °C) value of −10.1 kcal/mol. By contrast, the modified duplexes, dsRNA **6** and dsRNA **7**, showed less favorable enthalpic contributions, with similar Δ*H*° values of −68.5 kcal/mol and Δ*S*° values of −192.0 and −191.5 cal/mol·K, respectively. Correspondingly, the Δ*G*° (37 °C) values for dsRNA **6** and dsRNA **7** were reduced to −8.9 and −9.1 kcal/mol, respectively.

These results indicate that incorporating (*S*)‐*C*‐5′‐guanidinopropyl‐modified uridine analogs thermodynamically destabilizes the RNA duplex, primarily owing to less favorable enthalpic interactions. This is likely attributable to steric hindrance introduced by the bulky guanidino side chain at the 5′ position, which may interfere with optimal base pairing and stacking interactions during duplex formation.

Interestingly, the modified duplexes exhibited a modest entropy improvement compared with the native duplex, suggesting that the modified guanidino groups engage in additional stabilizing interactions, such as hydrogen bonding, with the complementary strand post‐duplex formation. However, this entropy gain was insufficient to compensate for the enthalpic penalty, resulting in an overall less favorable free energy for duplex formation.

In summary, while the introduction of 5′‐guanidinopropyl modifications led to thermodynamic destabilization driven by unfavorable enthalpy, the observed entropy improvements highlight the potential for side‐chain interactions to partially restore duplex stability. These findings contribute to our understanding of how site‐specific chemical modifications influence RNA duplex thermodynamics and may inform the future design of modified ONs for therapeutic and diagnostic applications.

### 2.4 Base‐Discriminating Ability

The base‐discrimination properties of modified RNA analogs **2** and **3** were systematically evaluated by analyzing the thermal stability (*T*_m_) of the RNA duplexes containing these modifications (Figure S6). The results were compared with those of the unmodified RNA duplexes hybridized to both fully complementary and single‐base‐mismatched RNA strands. In each case, the modified analog replaced uridine at defined positions within a consistent RNA sequence, allowing for the direct assessment of its effect on duplex stability (Table 4).

For fully complementary duplexes ( **X** = A), analogs **2** and **3** exhibited *T*_m_ values of 42.0 and 42.5 °C, respectively. These values were comparable to that of the unmodified RNA duplex (*T*_m_ = 42.3 °C), suggesting that the incorporation of analog **2** resulted in a slight reduction (Δ*T*_m_ = −0.3 °C), whereas analog **3** slightly increased duplex stability (Δ*T*_m_ = +0.2 °C). These minimal differences indicate that analog **2** slightly decreased the hybridization affinity with adenosine while preserving overall duplex integrity, whereas analog **3** appeared to enhance base‐pairing interactions. By contrast, single‐base mismatches involving uridine ( **X** = U) significantly destabilized the native duplex, with a *T*_m_ of 30.9 °C (Δ*T*_m_ = −11.4 °C). Incorporating analogs **2** and **3** further reduced duplex stability, with *T*_m_ values of 28.7 and 28.5 °C, corresponding to Δ*T*_m_ values of −13.3 and −14.0 °C, respectively. The results obtained confirm that both analogs improve mismatch discrimination, particularly against uridine–uridine mismatches, by decreasing tolerance to noncanonical base pairing. Similarly, mismatches with cytidine ( **X** = C) yielded *T*_m_ values of 28.9 °C for the unmodified duplex (Δ*T*_m_ = −13.4 °C), while analogs **2** and **3** showed slightly higher *T*_m_ values of 30.7 °C and 30.1 °C (Δ*T*_m_ = −11.3 and −12.4 °C, respectively). These data support the conclusion that analogs **2** and **3** exhibit low sensitivity to cytidine mismatches, demonstrating mismatch discrimination comparable to that of the unmodified duplex, although slightly lower than that of the unmodified duplex.

For guanosine mismatches ( **X** = G), the native duplex had a *T*_m_ of 40.0 °C, corresponding to Δ*T*_m_ = −2.3 °C. By contrast, analogs **2** and **3** showed more pronounced destabilization, with *T*_m_ values of 36.1 and 37.7 °C (Δ*T*_m_ = −5.9 and −4.8 °C, respectively). This greater destabilization suggests a reduced wobble base‐pairing potential, likely owing to alterations in stacking interactions or local conformational changes induced by modifications. Notably, the stronger propensity of the 2′‐fluoro modification in analog **3** to enforce a *C*3′‐*endo* sugar pucker, relative to the 2′‐*O*‐methyl group in analog **2**, may partially compensate for mismatch‐induced destabilization and contribute to the comparatively higher duplex stability observed for analog **3**. These effects enhance base‐pairing fidelity and reduce off‐target hybridization, which are critical for therapeutic applications.

Overall, these findings demonstrate that modifications **2** and **3** maintain the thermal stability of fully matched duplexes, while significantly improving discrimination against mismatches. The consistent destabilization observed for U:U mismatches confirmed the absence of wobble base‐pair formation, thereby enhancing base‐pairing specificity. Furthermore, the substantial destabilization observed in G‐mismatched duplexes supports the hypothesis that these analogs reduce wobble pairing and contribute to improved sequence selectivity.

These results confirm the importance of precise structural modifications in ON design. Specifically, the incorporation of 5′‐guanidinopropyl substitutions in analogs **2** and **3** resulted in a favorable balance between hybridization efficiency and mismatch discrimination. This dual capability highlights their potential applications in therapeutic nucleic acid technologies, where high sequence specificity and minimal off‐target effects are critical for accurate genetic interventions.

### 2.5 CD Spectra

**FIGURE 2:**
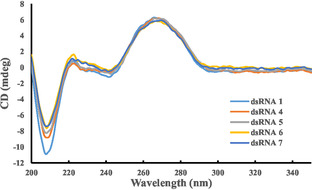
CD spectra of unmodified and modified dsRNAs recorded in a buffer containing 10 mM sodium phosphate (pH 7.0) and 100 nM NaCl at 25 °C. The duplex concentration was 4 μM.

Nucleic acids are known to adopt three principal types of tertiary structures: the A‐, B‐, and Z‐forms. Among these, RNA/RNA duplexes predominantly adopt an A‐form conformation. To investigate the influence of synthetic analog incorporation on RNA duplex architecture, we performed circular dichroism (CD) spectroscopy, a technique sensitive to nucleic acid conformational changes. The A‐form RNA duplex typically exhibits a characteristic CD spectrum with a strong negative band near 210 nm and a prominent positive band near 270 nm. In this study, RNA duplexes incorporating a 5′‐guanidinopropyl‐modified analog retained the overall spectral features of the A‐form, displaying a comparable positive maximum at ∼270 nm and a negative minimum near 210 nm (Figure 2).

However, a subtle but distinct reduction in the intensity of the negative band was observed in the CD spectra of the modified RNA duplexes compared with those of their unmodified counterparts. The extent of this decrease was slightly greater in the singly modified duplexes (dsRNA **4** and dsRNA **5**) than in the multiple modified duplexes (dsRNA **6** and dsRNA **7**), suggesting that the extent of modification influences the degree of perturbation of the local RNA structure. This attenuation of the negative CD signal implies that the 5′‐guanidinopropyl group, likely owing to its bulkiness and steric hindrance with adjacent nucleosides, induces localized conformational distortion in the RNA helix. Nevertheless, this distortion appears to be partially mitigated by favorable intramolecular interactions such as hydrogen bonds and electrostatic attraction afforded by the positively charged guanidino moiety.

Furthermore, thermodynamic analysis revealed that the incorporation of the 5′‐guanidinopropyl group led to a reduction in duplex stability, primarily through unfavorable changes in enthalpy. This thermodynamic destabilization aligns with the observed structural perturbations, reinforcing the notion that modification impairs duplex formation by subtly altering the higher‐order structure of RNA. Overall, while 5′‐guanidinopropyl modifications preserve the global A‐form conformation of RNA duplexes, they introduce localized distortions that affect both the CD spectral characteristics and the thermodynamic stability of the duplex, with the extent of perturbation being modulated by the number of modifications.

To further evaluate the structural impact of these modifications in the siRNA context, the CD spectra of unmodified and modified *KNTC2*‐targeting siRNAs (**1**–**7**) were recorded under identical conditions (Figure S7). siRNAs bearing analog **2** or **3** at the 5′ end of the passenger strand and at the 3′ overhangs of both strands displayed CD profiles nearly identical to those of the unmodified siRNA, retaining the characteristic A‐form signature without detectable perturbation of the negative band intensity. This behavior contrasts with the structural changes observed in the corresponding dsRNA constructs.

The CD spectra of siRNAs **2**–**7** were near‐mirror images of that of unmodified siRNA **1** and showed no evidence of structural destabilization, indicating that terminal and overhang modifications exert minimal influence on global helical conformation. This structural tolerance likely arises from the distal placement of the modifications relative to the central duplex and seed region, where the reduced base‐stacking constraints allow the accommodation of the bulky guanidinopropyl groups. These findings highlight the importance of modification positioning and demonstrate that strategic terminal incorporation preserves the structural integrity of siRNA, consistent with the retained RNAi activity of the modified constructs.

### 2.6 Nuclease Resistance of the Modified RNAs

**FIGURE 3:**
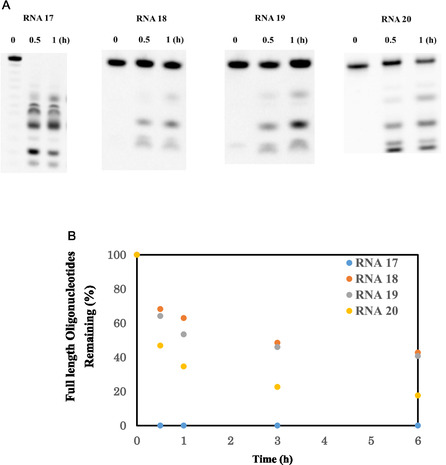
Results of the nuclease resistance assay using ssRNAs **17**–**20**. The reaction mixtures were incubated at 37 °C. PAGE analysis of ssRNAs at each incubation time (0, 0.5, 1, 3 and 6 h) with a BS concentration of 5%. (A) PAGE images obtained through luminescence analysis; (B) Time‐dependent profile of the percentage of remaining intact strands.

Natural RNA is inherently vulnerable to enzymatic degradation by nucleases present in biological fluids, such as serum, which poses a significant limitation for its application in therapeutic and diagnostic settings. To overcome this barrier, the incorporation of chemically modified nucleoside analogs has emerged as an effective strategy to enhance the nuclease resistance of RNA ONs. In this study, we evaluated the effect of introducing synthesized nucleoside analogs on the nuclease stability of single‐stranded RNA using bovine serum (BS) as a representative biological medium. Fluorescently labeled RNA ONs (Table 5) were incubated in a buffer containing 5% BS at physiological temperature (37 °C), and the degradation kinetics were monitored over time. The proportion of intact, full‐length RNA remaining at various time points (0, 0.5, 1, 3, and 6 h) was determined using 20% denaturing PAGE, followed by densitometric analysis using ImageJ software (Figure 3).

**TABLE 5:** | Sequences of ssRNAs used for nuclease resistance assay.

Abbreviation of ssRNA	Sequence^a^
RNA **17**	f‐5′‐ r (UUC UUC UUC UU) ‐3′
RNA **18**	f‐5′‐ r (U **1** C UUC **1** UC **1** U) ‐3′
RNA **19**	f‐5′‐ r (U **2** C UUC **2** UC **2** U) ‐3′
RNA **20**	f‐5′‐ r (U **3** C UUC **3** UC **3** U) ‐3′

a **1** **(** **orange** **)**, **2** **(** **red** **)**, and **3** **(** **blue** **)** indicate 5′‐Ap‐2′‐MoU (analog 1), 5′‐Gp‐2′‐MoU (analog 2), and 5′‐Gp‐2′‐FU (analog 3), respectively. f denotes fluorescein.

The unmodified RNA control (RNA **17**) rapidly degraded, with nearly complete loss of full‐length RNA within 30 min. By contrast, all the chemically modified RNAs exhibited markedly enhanced resistance to nuclease‐mediated degradation, with substantial amounts of full‐length RNA detectable after 6 h of incubation. These findings emphasize the protective effects of specific chemical modifications.

Among the modified ONs, RNA **18**, modified with 5′‐Ap‐2′‐MoU (analog **1**), was the most stable, retaining ∼42.7% of the intact strand after 6 h. Similarly, RNA **19**, incorporating 5′‐Gp‐2′‐MoU (analog **2**) modification, exhibited comparable resistance, with 40.7% of the strand remaining intact over the same time frame. By contrast, RNA **20**, which included the 5′‐Gp‐2′‐FU (analog **3**) modification, was much less stable: only 17.6% of the full‐length RNA remained after 6 h. The marked differences in stability among the modified ONs can be attributed to the chemical nature of the 2′‐substituents. The superior stability observed for RNAs **18** and **19** is likely due to the 2′‐*O*‐methyl group, which not only reduces ribose flexibility but also sterically hinders access to the 2′‐hydroxyl group, a key site of nuclease attack. Conversely, the smaller 2′‐fluoro group in RNA **20** lacks sufficient steric bulk to provide similar protection, rendering it more susceptible to enzymatic cleavage despite its electronegative nature. Additionally, the slight difference in nuclease resistance between RNA **18** and RNA **19** may be attributed to the nature of their 5′‐substituents. The 5′‐aminopropyl group in RNA **18**, being shorter and positively charged, likely contributes both steric hindrance and electrostatic repulsion, enhancing protection against nucleases. By contrast, the longer and more flexible 5′‐guanidinopropyl side chain in RNA **19** may result in reduced steric efficiency despite maintaining a positive charge, accounting for its slightly lower stability relative to RNA **18**.

Collectively, these results highlight the importance of dual chemical modifications for improving RNA stability under biologically relevant conditions. While all three analogs provided better protection than the unmodified RNA, the observed variations in the degradation profiles emphasize the necessity of optimizing both the geometry and physicochemical properties of RNA modifications to achieve maximal nuclease resistance. These findings support the strategic incorporation of 5′‐guanidinopropyl analogs alongside established modifications such as 2′‐*O*‐methylation to enhance RNA durability, a critical attribute for advancing RNA‐based therapeutics and diagnostics. Future investigations of the molecular mechanisms underlying these protective effects will be instrumental in guiding the rational design of robust nuclease‐resistant ON platforms.

### 2.7 Gene‐Silencing Activity of KNTC2‐Targeting siRNAs

To evaluate the functional performance of the synthesized guanidopropyl‐modified siRNAs, in vitro gene‐silencing studies were conducted in HCT116 human colon cancer cells targeting the kinetochore‐associated protein *KNTC2*. Seven *KNTC2*‐targeting siRNAs (siRNAs **1**– **7**; Table 2) were designed to systematically assess the effect of the position and type of modified nucleoside (analog **2** or analog **3**) on RNAi efficiency. The modifications were introduced either at the 5′ end or the 3′ overhang of the passenger (sense) strand, or at the 3′ overhang of the guide (antisense) strand.

**FIGURE 4:**
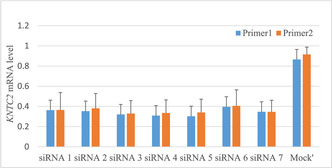
Relative *KNTC2* mRNA levels in human colon cancer HCT116 cells following treatment with siRNAs under lipofection conditions. Cells were transfected with siRNAs at a final concentration of 10 nM using 0.2% Lipofectamine RNAiMAX (LFA). After 24 h of incubation, the transfection medium was replaced with fresh medium, and the cells were cultured for an additional 48 h before mRNA analysis.

Cells were transfected with siRNAs at a final concentration of 10 nM using 0.2% Lipofectamine RNAiMAX (LFA). After 24 h of incubation, the medium was replaced, and the cells were further incubated for an additional 24 h before analysis. Relative *KNTC2* mRNA levels were quantified by qRT‐PCR using the ddCT method and normalized to those of mock‐treated cells (Figure 4). Under LFA‐mediated transfection conditions, all tested siRNAs exhibited robust gene‐silencing activity. Unmodified siRNA **1** reduced *KNTC2* mRNA levels by ∼63%, confirming the effectiveness of RNAi under these conditions. Importantly, all modified siRNAs containing either analog **2** or **3** demonstrated comparable silencing efficiencies, with *KNTC2* mRNA reduction ranging from approximately 59% to 67%.

Among the analog **2**–modified siRNAs, siRNA **2**, bearing the modification at the 5′ end of the passenger strand, achieved ∼62% knockdown. By contrast, siRNAs **3** and **4**, which incorporated analog **2** at the 3′ overhang of the passenger and guide strands, respectively, showed slightly enhanced silencing efficiencies (∼67% and ∼66%). This modest improvement is consistent with the known tolerance and functional preference of the RNAi machinery for chemical modifications at the 3′ overhang region. Similarly, the siRNAs modified with analog **3** maintained strong RNAi activity. siRNA **5** (5′‐modified passenger strand) and siRNA **7** (3′‐overhang‐modified guide strand) exhibited knockdown efficiencies of approximately 66% and 65%, respectively. By contrast, siRNA **6**, which contained analog **3** at the 3′ overhang of the passenger strand, displayed the lowest activity (∼59%); however, this reduction was modest and remained within the effective silencing range.

Overall, no substantial differences in gene‐silencing efficiency were observed between the modified and unmodified siRNAs under assisted transfection conditions. Incorporation of guanidopropyl‐modified nucleosides (5′‐Gp‐2′‐MoU, analog **2**, and 5′‐Gp‐2′‐FU, analog **3**) at the evaluated positions did not compromise RNAi activity, RISC loading, or target recognition when efficient cytoplasmic delivery was achieved using LFA. These results demonstrate that the introduced modifications are well tolerated by the RNAi machinery and preserve potent gene‐silencing activity, supporting their suitability for the development of chemically stabilized siRNA therapeutics.

## 3 Conclusions

In this study, two novel 5′‐guanidinopropyl‐modified uridine analogs, (*S*)‐5′‐C‐guanidinopropyl‐2′‐*O*‐methyluridine (5′‐Gp‐2′‐MoU, **2**) and (*S*)‐5′‐*C*‐guanidinopropyl‐2′‐fluorouridine (5′‐Gp‐2′‐FU, **3**), were successfully synthesized and incorporated into RNA ONs to evaluate their effects on duplex stability, structural integrity, sequence selectivity, nuclease resistance, and RNAi performance. Comprehensive physicochemical and biological investigations have demonstrated that both modifications are well‐tolerated within RNA frameworks and have properties beneficial to ON therapeutics. Thermal and thermodynamic analyses showed that single incorporation of 5′‐Gp‐2′‐MoU (**2**) resulted in only a modest reduction in duplex stability, whereas 5′‐Gp‐2′‐FU (**3**) imparted a slight stabilizing effect relative to the unmodified RNA duplexes. By contrast, multiple incorporations of either analog led to cumulative destabilization, indicating a clear dependence of thermodynamic stability on modification density. This destabilization was found to be predominantly enthalpy‐driven, consistent with localized perturbations of base stacking or hydrogen‐bonding interactions, while partial entropic compensation suggested favorable side‐chain or electrostatic interactions that helped preserve the overall duplex architecture.

CD spectroscopy confirmed that the modified dsRNAs retained their characteristic A‐form helical conformation with only minor spectral perturbations, whereas siRNAs bearing terminal or overhang modifications exhibited no detectable structural deviations. Importantly, mismatch discrimination studies have demonstrated the enhanced destabilization of mismatched duplexes, particularly U:U and U:G mismatches, indicating improved sequence selectivity. Furthermore, nuclease degradation assays revealed substantial enhancements in enzymatic stability, with 5′‐Gp‐2′‐MoU (**2**) being more resistant than 5′‐Gp‐2′‐FU (**3**) and affording protection comparable to the previously reported 5′‐Ap‐2′‐MoU (**1**). Finally, in vitro evaluation of *KNTC2*‐targeting siRNAs in HCT116 colon cancer cells demonstrated that incorporation of either analog at the 5′ end or 3′ overhang of the passenger strand, or at the 3′ overhang of the guide strand, did not compromise RNAi activity, RISC loading, or gene‐silencing efficiency under lipofection conditions. Altogether, these data establish 5′‐guanidinopropyl‐modified uridine analogs as versatile and effective nucleoside modifications that enhance nuclease resistance and sequence specificity while maintaining structural integrity and biological function, thereby supporting their further development for therapeutic RNA applications.

## 4 Experimental Section

### 4.1 General Remark

All chemicals and dry solvents (THF, CH_2_Cl_2_, and pyridine) were obtained from commercial sources (Fujifilm Wako, Osaka, Japan and TCI, Tokyo, Japan) and used without any further purification. Thin layer chromatography (TLC) was performed on silica gel plates precoated with a fluorescent indicator, with visualization by UV light or by dipping into a solution of 5% (v/v) concentrated H_2_SO_4_ in a mixture of *p*‐anisaldehyde and MeOH, then heating. Silica gel (63 –210 mesh) obtained from Kanto Chemical Company, Tokyo, Japan, was used for column chromatography. ^1^H NMR (400, 500 MHz), ^13^C {^1^H} NMR (101 MHz), and ^31^P NMR (162 MHz) were recorded on a 400 MHz JEOL spectrometer. CDCl_3_ was used as a solvent for obtaining NMR spectra. Chemical shifts (δ) are given in parts per million (ppm) from CDCl_3_ (7.26 ppm) for ^1^H NMR spectra; CDCl_3_ (77.2 ppm) for ^13^C NMR spectra; 80% H_3_PO_4_ (0.0 ppm) for ^31^P NMR. The abbreviations s, d, t, q, and m signify singlet, doublet, triplet, quadruplet, and multiplet, respectively. High‐resolution mass spectra (HRMS) were obtained in positive ion electrospray ionization (ESI‐TOF) mode.

#### 4.1.1 (*S*)*‐5′‐C‐Azidopropyl‐5′‐O‐*(*4*,*4′‐Dimethoxytrityl*)*‐2′‐O‐Methyluridine* (11a)

Under an argon atmosphere, compound **10a** (0.632 g, 0.72 mmol) was dissolved in THF (6.3 mL), and a 1 M solution of tetrabutylammonium fluoride (TBAF) (1.1 mL, 1.10 mmol) was added. The reaction mixture was stirred at room temperature for 3 h. Upon completion, the mixture was subjected to extraction with EtOAc and water. The organic phase was subsequently washed with a saturated aqueous solution of NaCl, dried over anhydrous Na_2_SO_4_, and concentrated under reduced pressure. The resulting residue was purified by silica gel column chromatography using a mixture of EtOAc and hexane (2:1, v/v) as the eluent, affording compound **11a** (0.397 g, 0.62 mmol, 86%) as a colorless solid. ^1^H NMR (400 MHz, CDCl_3_) δ: 8.09 (d, *J* = 8.2 Hz, 1H), 7.41–7.28 (m, 9H), 7.23 (q, *J* = 3.4 Hz, 1H), 6.84–6.81 (m, 4H), 5.94 (d, *J* = 2.3 Hz, 1H), 5.65 (d, *J* = 8.2 Hz, 1H), 4.16 (dd, *J* = 7.1, 5.7 Hz, 1H), 3.92 (dd, *J* = 7.3, 2.7 Hz, 1H), 3.80 (s, 6H), 3.59 (s, 3H), 3.55–3.52 (m, 1H), 3.03–2.85 (m, 2H), 1.70–1.62 (m, 1H), 1.49–1.34 (m, 2H), 1.24 (t, *J* = 6.9 Hz, 2H), 1.17–1.08 (m, 1H). ^13^C NMR (101 MHz, CDCl_3_) δ: 163.3, 158.9, 150.2, 146.0, 140.1, 136.4, 136.2, 130.8, 130.7, 128.6, 128.0, 127.3, 113.3, 102.4, 87.7, 87.1, 84.7, 83.9, 77.4, 72.9, 68.9, 59.0, 58.6, 55.4, 51.4, 29.1, 25.0, 18.6; HRMS (ESI‐TOF) *m/z:* Calcd. for [M + Na]^+^ C_34_H_37_N_5_NaO_8_; 666.25398, found 666.25331.

#### 4.1.2 (*S*)*‐5′‐C‐Azidopropyl‐5′‐O‐*(*4*,*4′‐Dimethoxytrityl*)*‐2′‐Fluorouridine* (11b)

Under an argon atmosphere, compound **10b** (1.26 g, 1.45 mmol) was dissolved in THF (13.0 mL), and a 1 M solution of tetrabutylammonium fluoride (TBAF) (2.2 mL, 2.20 mmol) was added. The reaction mixture was stirred at room temperature for 3 h. Upon completion, the mixture was subjected to extraction with EtOAc and water. The organic phase was subsequently washed with a saturated aqueous solution of NaCl, dried over anhydrous Na_2_SO_4_, and concentrated under reduced pressure. The resulting residue was purified by silica gel column chromatography using a mixture of EtOAc and hexane (3:2, v/v) as the eluent, affording compound **11b** (0.891 g, 1.41 mmol, 98%) as a colorless solid. ^1^H NMR (400 MHz, CDCl_3_) δ: 7.86 (d, *J* = 7.8 Hz, 1H), 7.47–7.28 (m, 9H), 7.25–7.21 (m, 1H), 6.83 (dt, *J* = 6.6, 2.7 Hz, 4H), 5.91 (dd, *J* = 17.6, 1.6 Hz, 1H), 5.71 (d, *J* = 8.2 Hz, 1H), 5.04 (ddd, *J* = 52.8, 4.7, 1.7 Hz, 1H), 4.26 (ddd, *J* = 19.0, 7.8, 4.8 Hz, 1H), 3.94 (dd, *J* = 7.8, 2.3 Hz, 1H), 3.80 (s, 6H), 3.64–3.59 (m, 1H), 3.03–2.97 (m, 1H), 1.69 (dd, *J* = 19.9, 8.9 Hz, 1H), 1.52–1.45 (m, 2H), 1.27 (q, *J* = 7.2 Hz, 2H), 1.22–1.18 (m, 1H). ^13^C NMR (101 MHz, CDCl_3_) δ: 163.0, 159.0, 149.9, 145.8, 140.5, 136.3, 136.1, 130.7, 128.5, 128.0, 127.3, 113.3, 102.8, 94.6, 92.7, 88.7, 88.4, 87.8, 83.5, 77.4, 72.1, 69.3, 69.1, 60.6, 55.5, 51.4, 29.1, 25.1, 21.2, 14.4; HRMS (ESI‐TOF) *m/z:* Calcd. for [M + K]^+^ C_33_H_34_FN_5_KO_7_; 670.20793, found 670.20591.

#### 4.1.3 (*S*)*‐5′‐C‐Aminopropyl‐5′‐O‐*(*4*,*4′‐Dimethoxytrityl*)*‐2′‐O‐Methyluridine* (12a)

Compound **11a** (0.397 g, 0.617 mmol) was dissolved in THF (8.0 mL), followed by the addition of triphenylphosphine (Ph_3_P) (0.404 g, 1.54 mmol) and distilled water (0.45 mL, 24.7 mmol). The reaction mixture was stirred at 40 °C for 6 h. Upon completion, the solvent was removed under reduced pressure to afford the crude product as a white solid, yielding compound **12a**, which was used in the subsequent step without further purification.

#### 4.1.4 (*S*)*‐5′‐C‐Aminopropyl‐5′‐O‐*(*4*,*4′‐Dimethoxytrityl*)*‐2′‐Fluorouridine* (12b)

Compound **11b** (0.891 g, 1.41 mmol) was dissolved in THF (17.0 mL), followed by the addition of triphenylphosphine (Ph_3_P) (0.923 g, 3.53 mmol) and distilled water (1.0 mL, 56.4 mmol). The reaction mixture was stirred at 40 °C for 6 h. Upon completion, the solvent was removed under reduced pressure to afford the crude product as a white solid, yielding compound **12a**, which was used in the subsequent step without further purification.

#### 4.1.5 (*S*)*‐5′‐C‐{N*, *N′‐Bis‐*[(*2‐Cyanoethoxy*)*‐Carbonyl*]*Guanidinopropyl}‐5′‐O‐*(*4*,*4′‐Dimethoxytrityl*)*‐2′‐O‐Methyluridine* (13a)

Under an argon atmosphere, compound **12a** was dissolved in CHCl_3_ (4.0 mL), and activated 4 A^o^ molecular sieves were added. The mixture was stirred at room temperature for 15 min, after which compound G4 (0.421g, 1.48 mmol), dissolved in a pyridine (0.23 mL, 2.96 mmol) /CHCl_3_ (4.0 mL) mixture, was added. The reaction mixture was stirred at 45 °C for 2 h. Upon completion, the mixture was extracted with CHCl_3_ and a saturated aqueous solution of NaCl. The organic layer was dried over anhydrous Na_2_SO_4_, and the solvent was removed under reduced pressure. The resulting residue was purified by silica gel column chromatography using a mixture of CHCl_3_ and methanol (200:1, v/v) as the eluent, affording compound **13a** (0.429 g, 0.502 mmol, 81%) as a colorless solid. ^1^H NMR (400 MHz, CDCl_3_) δ: 11.66 (s, 1H), 9.18 (s, 1H), 8.07–8.03 (m, 2H), 7.40–7.38 (m, 2H), 7.32–7.28 (m, 5H), 7.25–7.18 (m, 2H), 6.85–6.80 (m, 4H), 5.93 (d, *J* = 2.3 Hz, 1H), 5.66 (d, *J* = 7.8 Hz, 1H), 4.37 (t, *J* = 6.2 Hz, 2H), 4.28 (t, *J* = 6.6 Hz, 2H), 4.19–4.10 (m, 1H), 3.93 (q, *J* = 3.2 Hz, 1H), 3.83 (q, *J* = 2.6 Hz, 1H), 3.79 (d, *J* = 2.7 Hz, 6H), 3.58 (s, 3H), 3.50–3.47 (m, 1H), 3.15 (q, *J* = 6.3 Hz, 2H), 2.77–2.72 (m, 4H), 2.49 (d, *J* = 8.2 Hz, 1H), 1.64 (dd, *J* = 20.4, 9.4 Hz, 1H), 1.44–1.32 (m, 2H), 1.25–1.12 (m, 1H). ^13^C NMR (101 MHz, CDCl_3_) δ: 163.4, 163.0, 158.8, 155.8, 153.1, 150.3, 146.0, 140.1, 136.3, 136.2, 130.7, 130.6, 128.5, 127.9, 127.3, 117.2, 116.3, 113.2, 102.4, 87.6, 87.1, 84.6, 83.9, 77.4, 60.8, 59.9, 58.9, 55.4, 41.2, 28.9, 24.8, 18.2, 18.1; HRMS (ESI‐TOF) *m/z:* Calcd. for [M + Na]^+^ C_43_H_47_N_7_NaO_12_; 876.31804, found 876.32178.

#### 4.1.6 (*S*)*‐5′‐C‐{N*, *N′‐Bis‐*[(*2‐Cyanoethoxy*)*‐Carbonyl*]*Guanidinopropyl}‐5′‐O‐*(*4*,*4′‐Dimethoxytrityl*)*‐2′‐Fluorouridine* (13b)

Under an argon atmosphere, compound **12b** was dissolved in CHCl_3_ (8.6 mL), and activated 4 A^o^ molecular sieves were added. The mixture was stirred at room temperature for 15 min, after which compound G4 (0.961 g, 3.38 mmol), dissolved in a pyridine (0.55 mL, 6.76 mmol)/CHCl_3_ (8.6 mL) mixture, was added. The reaction mixture was stirred at 45 °C for 2 h. Upon completion, the mixture was extracted with CHCl_3_ and a saturated aqueous solution of NaCl. The organic layer was dried over anhydrous Na_2_SO_4_, and the solvent was removed under reduced pressure. The resulting residue was purified by silica gel column chromatography using a mixture of CHCl_3_ and methanol (50:1, v/v) as the eluent, affording compound **13b** (0.971 g, 1.15 mmol, 81%) as a colorless solid. ^1^H NMR (400 MHz, CDCl_3_) δ: 11.66 (s, 1H), 8.75 (s, 1H), 8.11 (t, *J* = 5.5 Hz, 1H), 7.81–7.76 (m, 1H), 7.46–7.40 (m, 2H), 7.36–7.27 (m, 5H), 7.25–7.20 (m, 2H), 6.85–6.81 (m, 4H), 5.89–5.84 (m, 1H), 5.69 (dd, *J* = 8.2, 2.3 Hz, 1H), 5.12–4.98 (m, 1H), 4.38 (t, *J* = 6.4 Hz, 2H), 4.28 (dd, *J* = 7.1, 6.2 Hz, 2H), 4.23 (dd, *J* = 8.0, 4.8 Hz, 1H), 4.02 (dd, *J* = 8.0, 3.0 Hz, 1H), 3.79 (d, *J* = 2.3 Hz, 6H), 3.62–3.60 (m, 1H), 3.25–3.08 (m, 2H), 2.78–2.71 (m, 4H), 2.31 (dd, *J* = 8.2, 2.3 Hz, 1H), 1.58 (dd, *J* = 12.8, 9.2 Hz, 1H), 1.46–1.37 (m, 2H), 1.33–1.21 (m, 1H). ^13^C NMR (101 MHz, CDCl_3_) δ: 163.0, 162.9, 158.8, 155.8, 153.2, 149.9, 145.8, 140.6, 136.2, 136.2, 130.7, 130.6, 128.5, 127.9, 127.2, 117.2, 116.3, 113.2, 102.6, 94.6, 92.7, 89.0, 88.7, 87.5, 83.2, 77.3, 72.1, 69.2, 69.1, 60.7, 59.8, 55.4, 41.0, 28.6, 24.7, 18.2, 18.1; HRMS (ESI‐TOF) *m/z:* Calcd. for [M + Na]^+^ C_42_H_44_FN_7_NaO_11_; 864.29805, found 864.29837.

#### 4.1.7 *3′‐O‐*(*2‐Cyanoethyl‐N*, *N‐Diisopropylphosphoramidite*)*‐*(*S*)*‐5′‐C‐{N*, *N′‐bis‐*[(*2‐Cyanoethoxy*)*‐Carbonyl*]*Guanidinopropyl}‐5′‐O‐*(*4*,*4′‐Dimethoxytrityl*)*‐2′‐O‐Methyluridine* (14a)

Under an argon atmosphere, compound **13a** (0.429 g, 0.502 mmol) was dissolved in THF (4.3 ml), followed by the addition of *N*,*N*‐diisopropylethylamine (DIPEA) (0.225 mL, 1.00 mmol) and 2‐cyanoethyl *N*,*N*‐diisopropylchlorophosphoramidite (CEP‐Cl) (0.439 mL, 2.51 mmol). The reaction mixture was stirred at room temperature for 3 h. Upon completion, the mixture was extracted with CHCl_3_ and a saturated aqueous solution of NaHCO_3_. The organic layer was subsequently washed with a saturated aqueous solution of NaCl, dried over anhydrous Na_2_SO_4_, and concentrated under reduced pressure. The resulting residue was purified by silica gel column chromatography using EtOAc and CHCl_3_ (2:1, v/v) as the eluent, affording compound **14a** (0.451 g, 0.428 mmol, 85%) as a colorless solid. ^31^P NMR (162 MHz, CDCl_3_) δ: 151.9, 150.8, 150.2; HRMS (ESI‐TOF) *m/z:* Calcd. for [M + K] ^+^ C_52_H_64_KN_9_O_13_P; 1092.39982, found 1092.39629.

#### 4.1.8 *3′‐O‐*(*2‐Cyanoethyl‐N*, *N‐Diisopropylphosphoramidite*)*‐*(*S*)*‐5′‐C‐{N*, *N′‐bis‐*[(*2‐Cyanoethoxy*)*‐Carbonyl*]*Guanidinopropyl}‐5′‐O‐*(*4*,*4′‐Dimethoxytrityl*)*‐2′‐Fluorouridine* (14b)

Under an argon atmosphere, compound **13b** (0.971 g, 1.15 mmol) was dissolved in THF (10.0 mL), followed by the addition of *N*,*N*‐diisopropylethylamine (DIPEA) (0.515 mL, 2.31 mmol) and 2‐cyanoethyl *N*,*N*‐diisopropylchlorophosphoramidite (CEP‐Cl) (1.01 mL, 5.77 mmol). The reaction mixture was stirred at room temperature for 3 h. Upon completion, the mixture was extracted with CHCl_3_ and a saturated aqueous solution of NaHCO_3_. The organic layer was subsequently washed with a saturated aqueous solution of NaCl, dried over anhydrous Na_2_SO_4_, and concentrated under reduced pressure. The resulting residue was purified by silica gel column chromatography using EtOAc and CHCl_3_ (2:1, v/v) as the eluent, affording compound **14b** (0.107 g, 1.03 mmol, 89%) as a colorless solid. ^31^P NMR (162 MHz, CDCl_3_) δ: 151.3, 151.0, 150.9; HRMS (ESI‐TOF) *m/z:* Calcd. for [M + Na]^+^ C_51_H_61_FN_9_NaO_12_P; 1064.40590, found 1064.40161.

### 4.2 Solid‐Phase Oligonucleotide Synthesis

ONs were synthesized on a 0.2 μmol scale using an automated DNA/RNA synthesizer and standard solid‐phase phosphoramidite chemistry on a universal solid support. Phosphoramidites of natural RNA, as well as 2′‐*O*‐methyl‐ and 2′‐fluoro‐modified nucleosides, were prepared at a concentration of 0.10 M in acetonitrile (CH_3_CN). Phosphoramidites bearing modified nucleoside analogs, including 5′‐guanidinopropyl‐modified residues, were prepared at a concentration of 0.15 M in CH_3_CN. The phosphoramidite of (*S*)‐5′‐aminopropyl‐2′‐*O*‐methyluridine, employed for comparative studies, was synthesized and handled as previously reported and used at a concentration of 0.15 M in CH_3_CN. All coupling reactions were activated using 0.25 M benzylthio‐1*H*‐tetrazole in CH_3_CN. The coupling efficiencies of the 5′‐guanidinopropyl‐modified nucleoside phosphoramidites were comparable to those of conventional RNA phosphoramidites, demonstrating their compatibility with automated ON synthesis. Following chain assembly, the cyanoethyl protecting groups were removed by treating the CPG‐bound ONs with 10% diethylamine in CH_3_CN. For ONs composed exclusively of natural RNA or containing free amino functionalities, cleavage from the solid support and base deprotection were carried out using a 1:1 (v/v) mixture of 28% aqueous ammonia and 40% aqueous methylamine at 65 °C for 10 min. For ONs containing 5′‐guanidinopropyl‐modified nucleosides, initial cleavage and deprotection using 50% piperidine in water resulted in undesired side reactions, specifically nucleophilic substitution at the C6 position of guanine. To suppress this reaction, the deprotection conditions were re‐optimized. Treatment of the CPG‐bound ONs with a mixture of H_2_O and aqueous ammonia (1:3, v/v; 1.0 mL) at 55 °C for 8 h efficiently afforded the desired ONs without detectable side reactions. Using these optimized conditions, siRNAs **1**–**7** were successfully obtained with high integrity. Subsequent removal of the 2′‐*O*‐*tert*‐butyldimethylsilyl (2′‐OTBDMS) protecting groups was achieved by treatment with triethylamine trihydrofluoride (Et_3_N·3HF) in DMSO at 65 °C for 90 min, followed by quenching with 0.1 M triethylammonium acetate (TEAA) buffer (pH 7.0). After deprotection, the entire crude ON product obtained from each 0.2 μmol synthesis was purified by 20% denaturing PAGE. The purified ONs were recovered by passive elution, desalted using a Sep‐Pak C18 cartridge, and lyophilized. The identity and integrity of the synthesized ONs were confirmed by MALDI–TOF mass spectrometry, and their purity was further assessed by HPLC analysis (Figure S1 and S2).

### 4.3 Reverse‐Phase HPLC Analysis of Oligonucleotides

The purity of all synthesized ONs was assessed by reverse‐phase high‐performance liquid chromatography (RP‐HPLC) using a YMC‐Triart C18 wide‐pore column (YMC Co., Ltd.). For RNA **1**–**8** and RNA **17**–**20**, the analysis was performed using a linear gradient elution program starting from 100% buffer A, increasing to 10% buffer B over 1 min, and then to 25% buffer B over 25 min. For RNA **9**–**16**, elution was initiated with 100% buffer A, followed by a linear gradient to 20% buffer B at 1 min and then to 25% buffer B at 25 min. In all analyses, the flow rate was maintained at 1.0 mL min^−1^. Buffer A consisted of 5% acetonitrile (MeCN) in 0.1 M triethylammonium acetate (TEAA) buffer (pH 7.0), whereas buffer B consisted of 50% MeCN in 0.1 M TEAA buffer (pH 7.0).

### 4.4 Thermal Denaturation Study

Thermal denaturation studies were conducted using RNA duplexes, each prepared at a final concentration of 3.0 μM. ONs (600 pmol) were dissolved in a buffer containing 10 mM sodium phosphate (pH 7.0) and 100 mM NaCl. Duplex formation was achieved by heating the samples to 100 °C, followed by gradual cooling to room temperature. Thermal denaturation profiles were recorded by monitoring absorbance at 260 nm using a UV–Vis spectrophotometer equipped with a temperature‐controlled quartz cuvette (path length: 1.0 cm). The temperature was increased at a rate of 0.5 °C per minute. Thermodynamic parameters of duplex formation were determined by analyzing the melting temperature (*T*_m_) as a function of strand concentration. Specifically, the thermodynamic parameters of the duplexes on duplex formation were determined by calculations based on the slope of a 1/*T*_m_ versus ln (C_T_/4) plot, where CT (1, 3, 6, 12, 15, 21, 16, 30, and 60 μM) is the total concentration of single strands.

### 4.5 CD Spectroscopy

CD spectra were recorded at 25 °C to analyze the secondary structure of RNA duplexes. ONs (1200 pmol) were dissolved in a buffer containing 10 mM sodium phosphate (pH 7.0) and 100 mM NaCl, yielding a final duplex concentration of 4.0 μM. Duplex formation was achieved through annealing, wherein the samples were heated to 100 °C for 5 min, followed by gradual cooling to room temperature over the course of 1 h. CD measurements were conducted using a spectropolarimeter under the following conditions: spectral resolution of 0.1 nm, response time of 1.0 s, scan speed of 50 nm/min, and an accumulation of 10 scans to enhance signal quality.

### 4.6 Nuclease resistance

The nuclease resistance of single‐stranded RNA was evaluated using fluorescein‐labeled ONs. Labeled ssRNAs (300 pmol) were dissolved in 37.5 μL of Opti‐MEM medium and subsequently incubated with 1.9 μL of bovine serum (final concentration: 5% serum) at 37 °C. Aliquots (5 μL) were collected at designated time points (0, 0.5, 1, 3, and 6 h) and immediately mixed with a loading buffer containing 10 mM EDTA in formamide (10 μL) to halt enzymatic degradation. Samples were then analyzed by 20% PAGE under denaturing conditions at a constant current of 20 mA for 3 h. Gel visualization was performed using a Luminescent Image Analyzer LAS‐4000 (Fujifilm).

### 4.7 Evaluation of gene expression suppression ability

Human colon cancer HCT116 cells were seeded at a density of 2 **×** 10^5^ cells per well in a 24‐well plate and incubated overnight. The following day, siRNA was introduced at a final concentration of 10 nM in the presence of 0.2% Lipofectamine RNAiMAX (LFA) using culture medium supplemented with 8% fetal bovine serum (FBS). After 48 h of transfection, the culture medium was replaced with fresh Dulbecco’s Modified Eagle Medium (DMEM) containing 10% FBS. On day 5, total RNA was extracted, and quantitative real‐time polymerase chain reaction (qRT‐PCR) was performed to assess gene expression levels. Complementary DNA (cDNA) was synthesized using the PrimeScript RT Master Mix (Takara Bio), and qRT‐PCR was conducted with the TB Green Premix Ex Taq II (Takara Bio) on a Thermal Cycler Dice Real‐Time System (Takara Bio). The primer sequences used were as follows: *KNTC2* Primer 1 forward 5′‐CCTCTCCATGCAGGAGTTAAGA‐3′ and reverse 5′‐GGTCTCGGGTCCTTGATTTTCT‐3′; *KNTC2* Primer 2 forward 5′‐GGTCTCAATGAGGAAATTGCTAGA‐3′ and reverse 5′‐GGTTGTCAATGATATTCTGTAGTCG‐3′. All reactions were performed in duplicate, and relative gene expression levels were determined using the ΔΔCT method.

## Funding

This study was supported by the Japan Agency for Medical Research and Development (24ae0121029h0004).

## Conflicts of Interest

The authors declare no conflicts of interest.

## Supporting information

Supplementary Material

## Data Availability

The data that support the findings of this study are available in the Supporting Information of this article.
