# Supplementary material for: Structural and Functional Insights Into (S)‐5′‐C‐Guanidinopropyl Uridine‐Modified Oligonucleotides With 2′‐O‐Methyl and 2′‐Fluoro Substitutions for RNAi Therapeutics
Source: ChemMedChem. 2026 Aug 7;21(15):e70413. doi: 10.1002/cmdc.70413 (PMC13451541; doi:10.1002/cmdc.70413)

Supplementary Materials

Structural and Functional Insights into (*S*)-5′-*C*-Guanidinopropyl Uridine-Modified Oligonucleotides with 2′-*O*-Methyl and 2′-Fluoro Substitutions for RNAi Therapeutics

Sugahara Masaki,[1] Elsayed M. Mahmoud,[2, 6] Shuichi Sakamoto,[5] and Yoshihito Ueno [1, 2, 3, 4, *]

[1]United Graduate School of Agricultural Science, Gifu University, 1-1 Yanagido, Gifu, 501-1193, Japan.

[2]Course of Applied Life Science, Faculty of Applied Biological Sciences, Gifu University, 1-1 Yanagido, Gifu, 501-1193, Japan.

[3]Department of Life Science and Chemistry, the Graduate School of Natural Science and Technology, Gifu University, 1-1 Yanagido, Gifu, 501-1193, Japan.

[4]Center for One Medicine Innovative Translational Research (COMIT), Tokai National Higher Education and Research System, Gifu University 1-1 Yanagido, Gifu, 501-1193, Japan.

[5]Institute of Microbial Chemistry (BIKAKEN) Numazu Branch, Microbial Chemistry Research Foundation, 18-24 Miyamoto, Numazu, Shizuoka 410-0301, Japan.

[6]Department of Pharmaceutical Organic Chemistry, Faculty of Pharmacy, Zagazig University, Zagazig, 44519, Egypt

*To whom correspondence should be addressed.

Phone: +81-58-293-2919; Fax: +81-58-293-2919.

E-mail: ueno.yoshihito.e7@f.gifu-u.ac.jp

Table of Contents

1. MALDI-TOF MS Analysis of Oligonucleotides ....S**Error! Bookmark not defined.**

2. Figure S1. MALDI-TOF Mass Spectra of ssRNAs **1**–**20** .S**Error! Bookmark not defined.**

3. Figure S2. HPLC Characterization of ssRNAs **1**–**20** ....S**Error! Bookmark not defined.**

4. Figure S3. UV Melting Profiles of Modified and Unmodified dsRNAs (**1**–**7**) and siRNAs (**1**–**7**) Incorporating Different Modification Designs. S3

5. Figure S4. UV Melting Curves of dsRNAs at Various Concentrations for Thermodynamic Analysis. S4

6. Figure S5. Plots of $1/T_m$ versus ln(CT/4) for dsRNAs **1**, **6**, and **7**. S5

7. Figure S6. UV Melting Profiles of Modified and Unmodified dsRNAs Used for Base-Discrimination Analysis.. .... S5

8. Figure S7. Circular Dichroism (CD) Spectra of *KNTC2*-Targeting siRNAs...................... S6

9. Copies of NMR spectra (^{1}H, ^{13}C, and 31P) S7

1. MALDI-TOF MS Analysis of Oligonucleotides

The spectra were obtained with a time-of-flight mass spectrometer equipped with nitrogen laser (337 nm, 3 ns pulse). A solution of 3-hydroxypicolinic acid (3-HPA) and diammonium hydrogen citrate in H_2O was used as the matrix. Data of synthetic RNAs:

RNA **1**: *m*/*z* = 3302.72 (calcd for $C_{99}H_{125}N_{25}O_{83}P_{10}$ $[M-H]^-$, 3302.91); RNA **2**: *m*/*z* = 3607.70 (calcd for $C_{110}H_{133}N_{55}O_{67}P_{10}$ $[M-H]^-$, 3607.30); RNA **3**: *m*/*z* = 3317.03 (calcd for $C_{100}H_{127}N_{25}O_{83}P_{10}$ $[M-H]^-$, 3316.93); RNA **4**: *m*/*z* = 3304.06 (calcd for $C_{99}H_{124}FN_{25}O_{82}P_{10}$ $[M-H]^-$, 3304.90); RNA **5**: *m*/*z* = 3416.25 (calcd for $C_{104}H_{136}N_{28}O_{83}P_{10}$ $[M-H]^-$, 3415.87); RNA **6**: *m*/*z* = 3403.57 (calcd for $C_{103}H_{133}FN_{28}O_{82}P_{10}$ $[M-H]^-$, 3403.85); RNA **7**: *m*/*z* = 3642.17 (calcd for $C_{114}H_{158}N_{34}O_{83}P_{10}$ $[M-H]^-$, 3641.80); RNA **8**: *m*/*z* = 3605.86 (calcd for $C_{111}H_{149}F_3N_{34}O_{80}P_{10}$ $[M-H]^-$, 3605.74); RNA **9**: *m*/*z* = 6843.49 (calcd for $C_{214}H_{272}F_4N_{68}O_{147}P_{20}$ $[M-H]^-$, 6843.75); RNA **10**: *m*/*z* = 6871.59 (calcd for $C_{215}H_{270}F_6N_{78}O_{137}P_{20}$ $[M-H]^-$, 6871.58); RNA **11**: *m*/*z* = 6941.60 (calcd for $C_{218}H_{281}F_4N_{71}O_{147}P_{20}$ $[M-H]^-$, 6942.89); RNA **12**: *m*/*z* = 6943.27 (calcd for $C_{218}H_{281}F_4N_{71}O_{147}P_{20}$ $[M-H]^-$, 6942.89); RNA **13**: *m*/*z* = 6971.14 (calcd for $C_{219}H_{279}F_6N_{81}O_{137}P_{20}$ $[M-H]^-$, 6970.72); RNA **14**: *m*/*z* = 6929.03 (calcd for $C_{217}H_{278}F_5N_{71}O_{146}P_{20}$ $[M-H]^-$, 6930.86); RNA **15**: *m*/*z* = 6930.84 (calcd for $C_{217}H_{278}F_5N_{71}O_{146}P_{20}$ $[M-H]^-$, 6930.86); RNA **16**: *m*/*z* = 6958.08 (calcd for $C_{218}H_{276}F_7N_{81}O_{136}P_{20}$ $[M-H]^-$, 6958.69); RNA **17**: *m*/*z* = 3841.85 (calcd for $C_{126}H_{150}N_{26}O_{92}P_{11}$ $[M-H]^-$, 3840.37); RNA **18**: *m*/*z* = 4053.92 (calcd for $C_{138}H_{177}N_{29}O_{92}P_{11}$ $[M-H]^-$, 4053.74); RNA **19**: *m*/*z* = 4181.34 (calcd for $C_{141}H_{183}N_{35}O_{92}P_{11}$ $[M-H]^-$, 4179.26); RNA **20**: *m*/*z* = 4144.93 (calcd for $C_{138}H_{174}F_3N_{35}O_{89}P_{11}$ $[M-H]^-$, 4143.20).

2. Figure S1. MALDI-TOF Mass Spectra of ssRNAs **1–20**

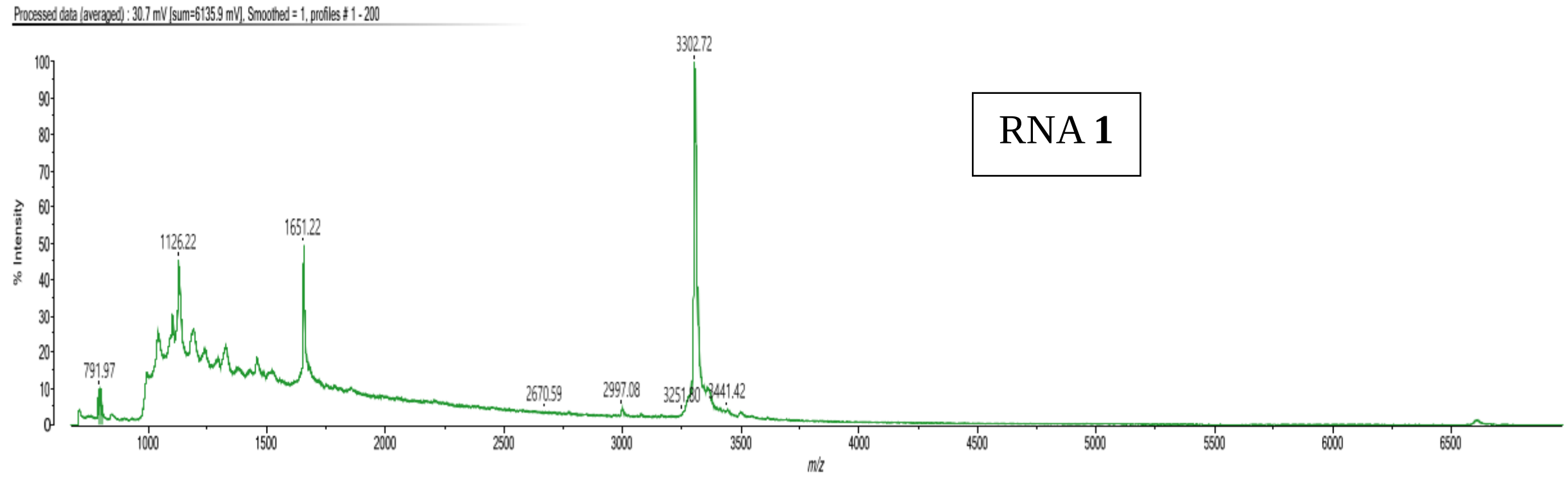

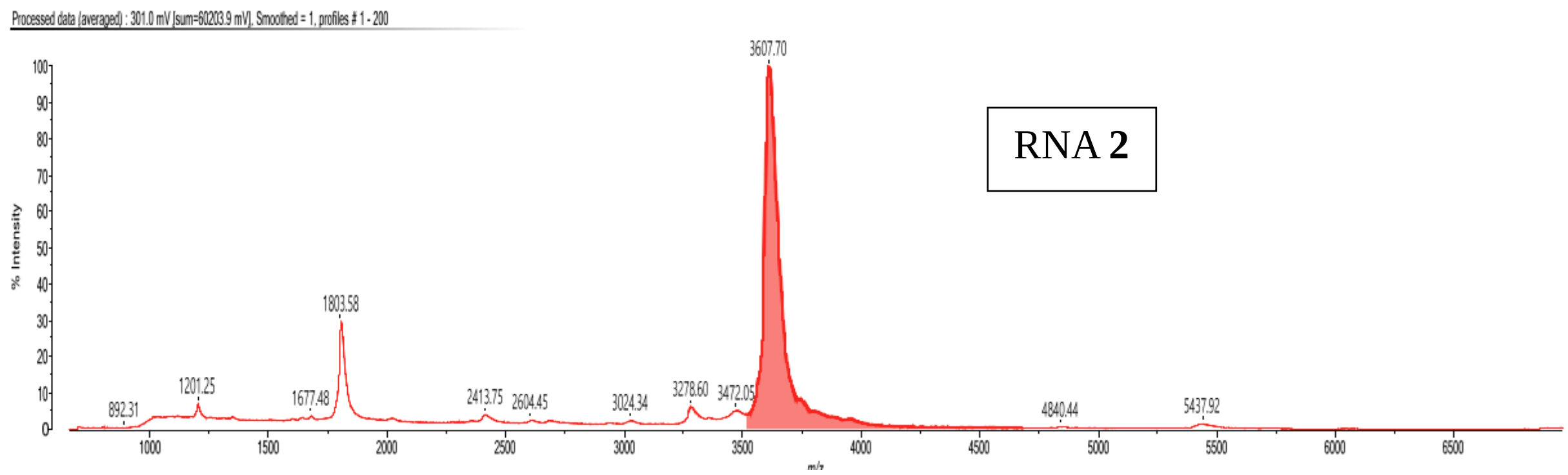

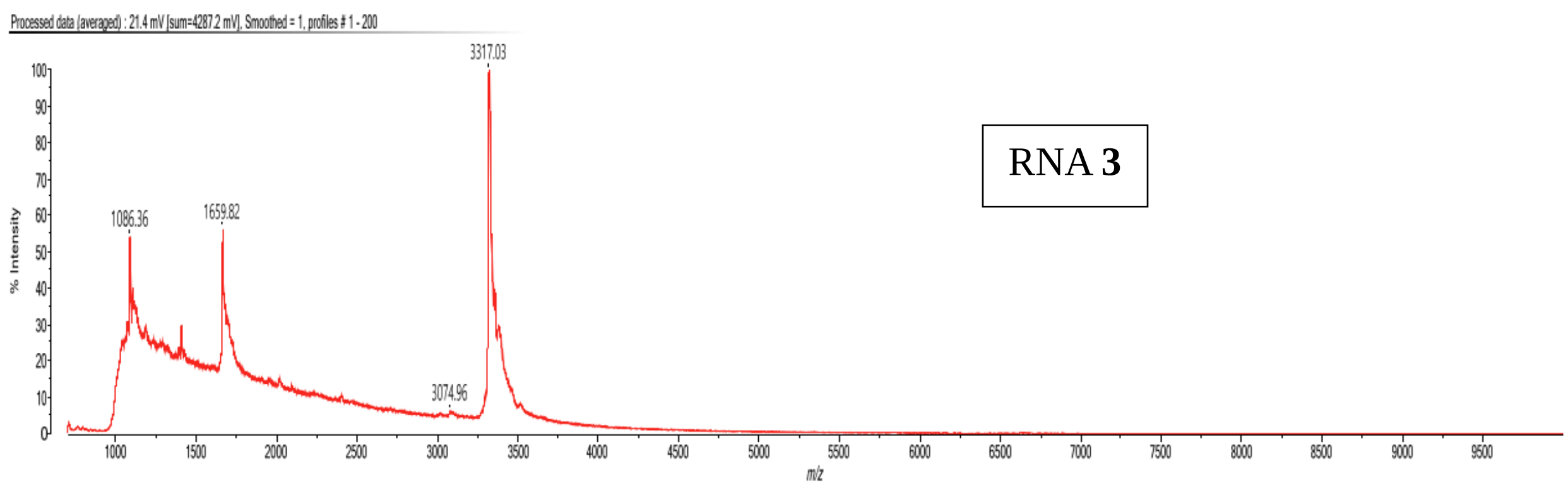

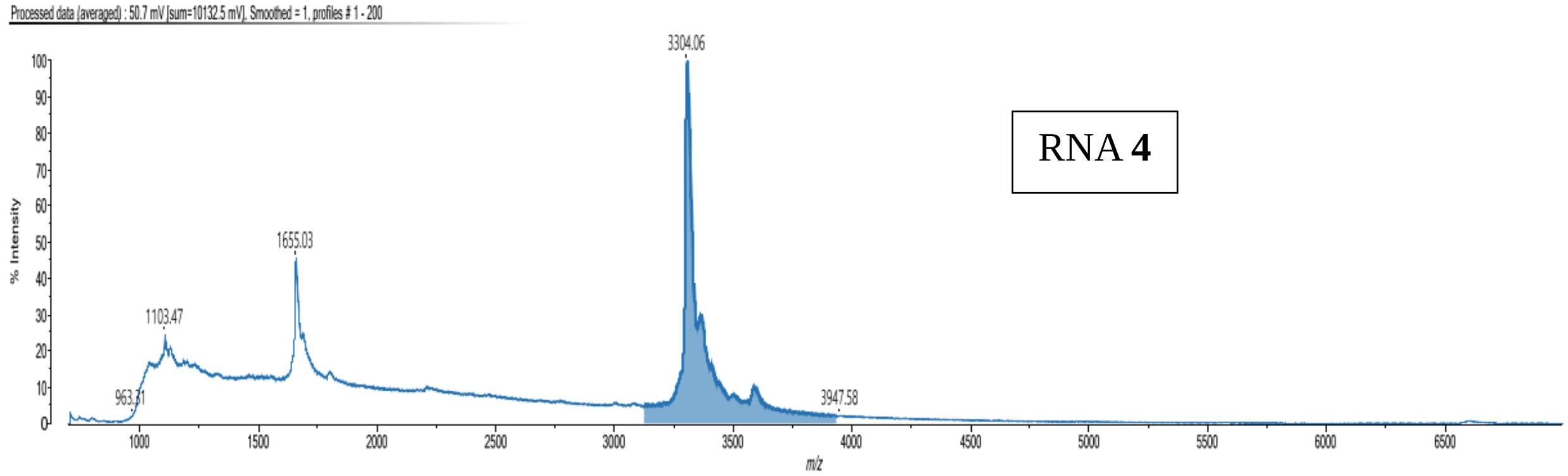
Processed data (averaged) : 50.7 mV [sum=10132.5 mV], Smoothed = 1, profiles # 1 - 200
RNA 4
3304.06
1655.03
1103.47
963.31
3947.58
% Intensity
m/z

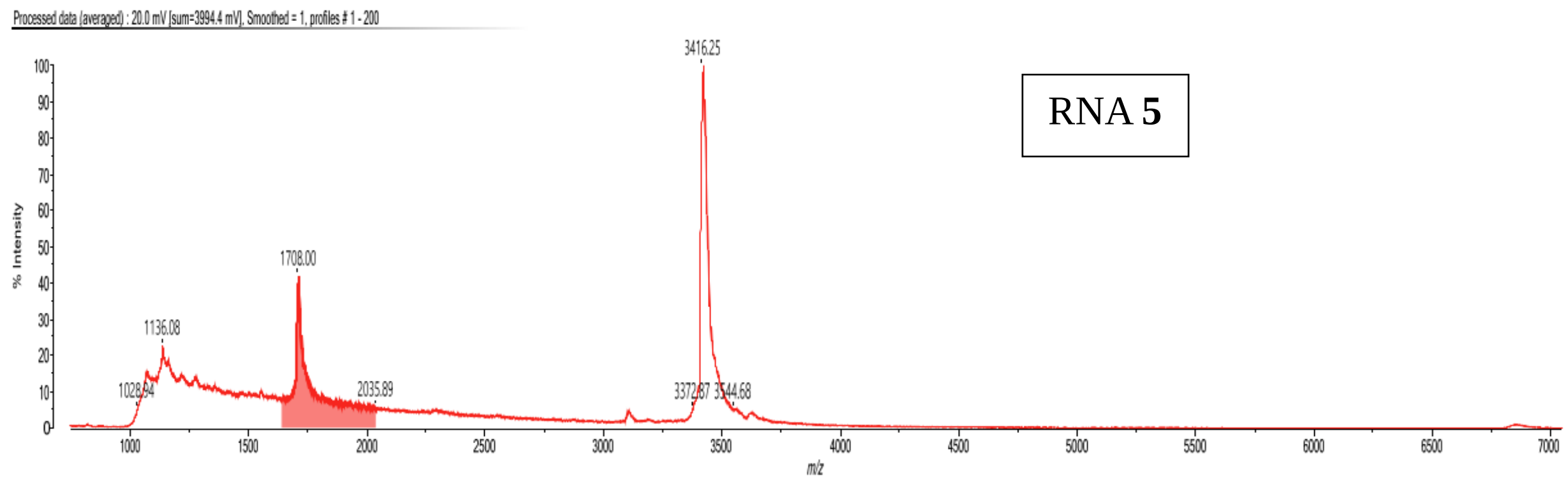
Processed data (averaged) : 20.0 mV [sum=3994.4 mV], Smoothed = 1, profiles # 1 - 200
RNA 5
3416.25
1708.00
1136.08
1028.94
2035.89
3372.67
3544.68
% Intensity
m/z

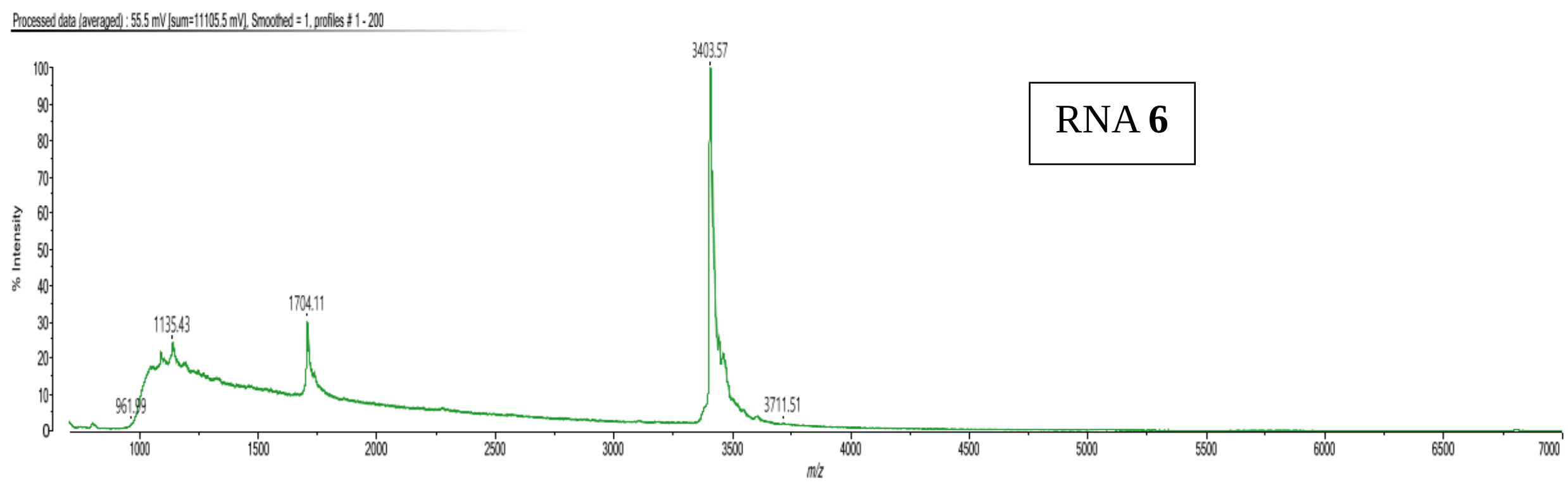
Processed data (averaged) : 55.5 mV [sum=11105.5 mV], Smoothed = 1, profiles # 1 - 200
RNA 6
3403.57
1704.11
1135.43
961.99
3711.51
% Intensity
m/z

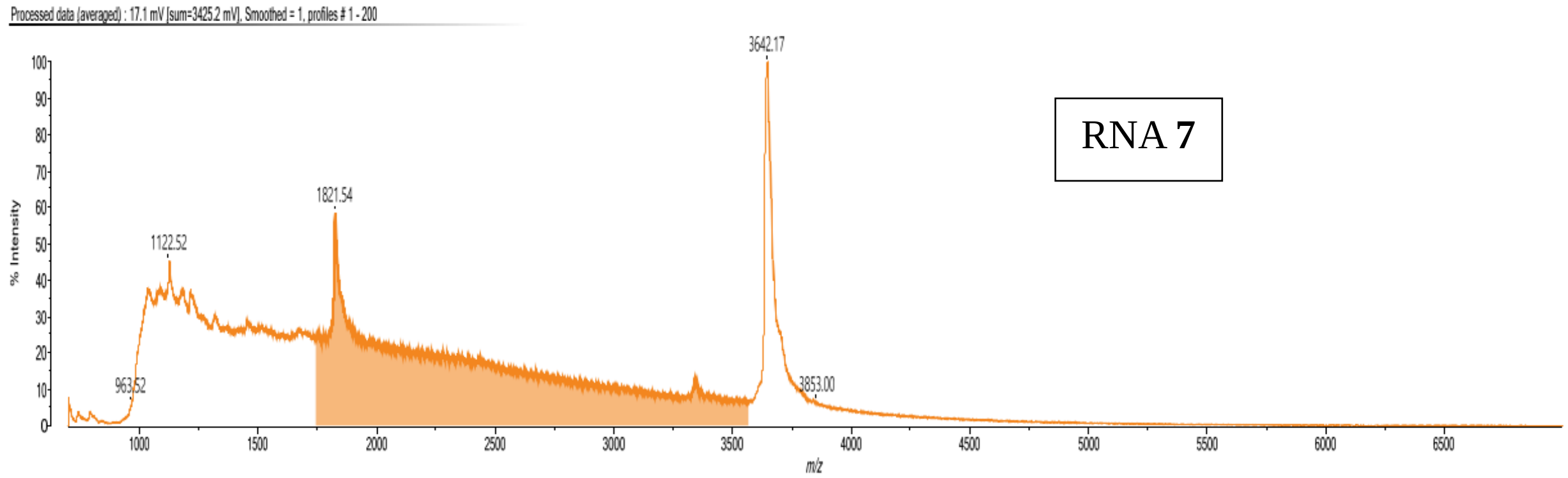
Processed data (averaged) : 17.1 mV [sum=3425.2 mV], Smoothed = 1, profiles # 1 - 200
RNA 7
3642.17
1821.54
1122.52
963.52
3853.00
% Intensity
m/z

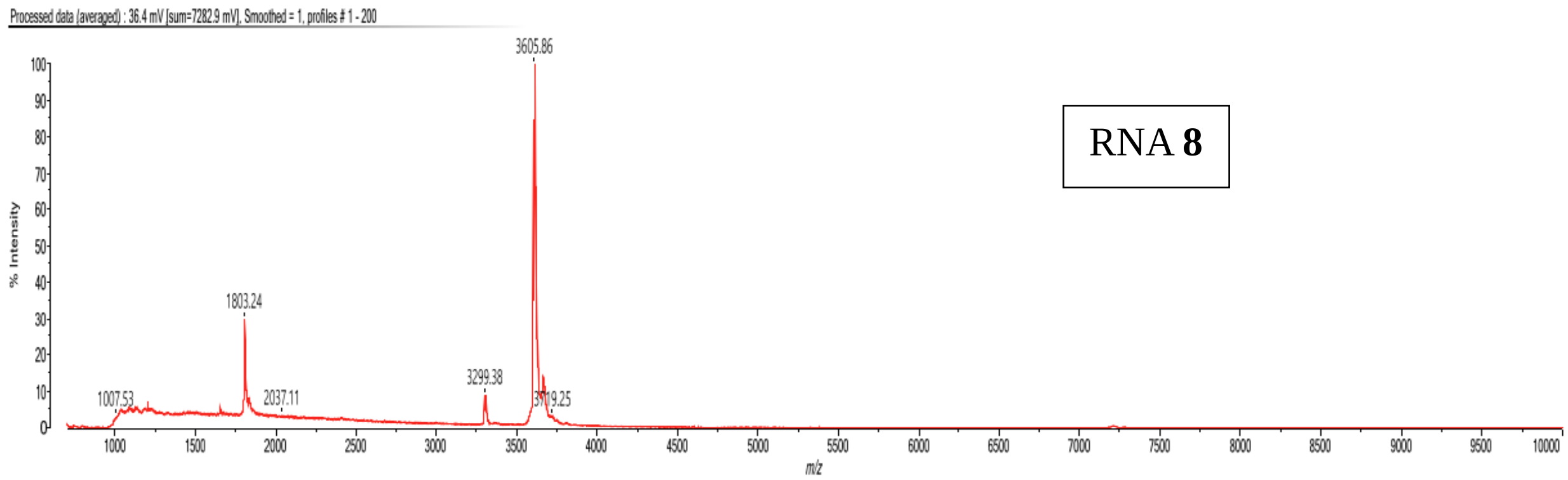
Processed data (averaged) : 36.4 mV [sum=7282.9 mV], Smoothed = 1, profiles # 1 - 200
RNA 8
3605.86
1803.24
1007.53
2037.11
3299.38
% Intensity
m/z

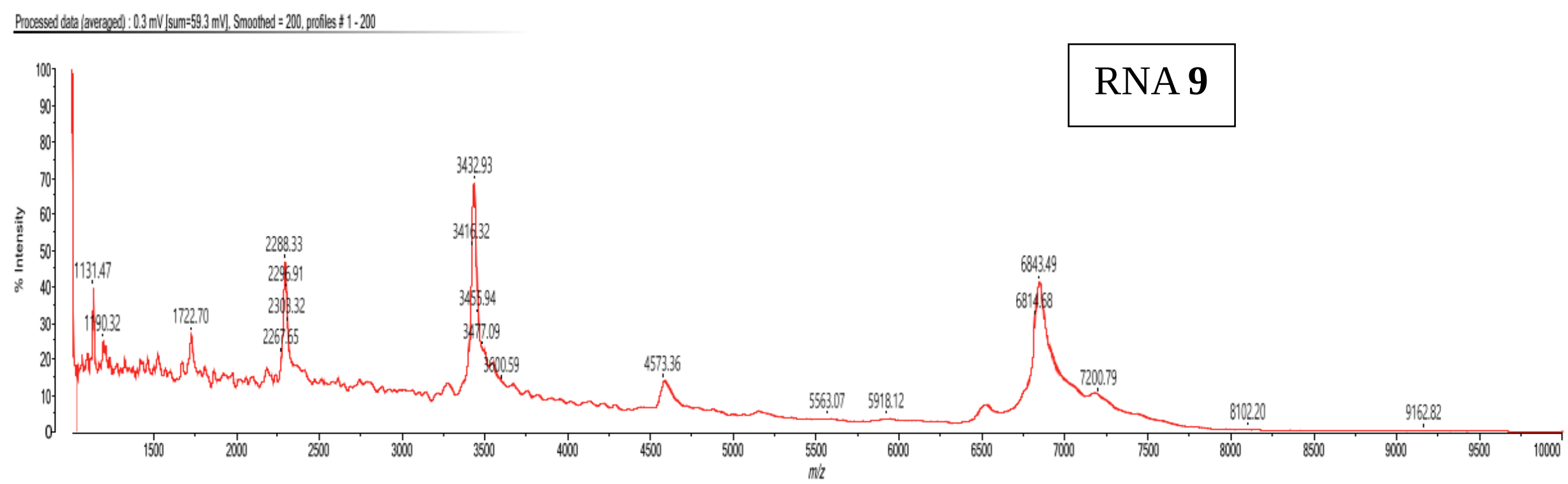
Processed data (averaged) : 0.3 mV [sum=59.3 mV], Smoothed = 200, profiles # 1 - 200
RNA 9
3432.93
2288.33
1131.47
1722.70
4573.36
6843.49
7200.79
5563.07
5918.12
8102.20
9162.82
% Intensity
m/z

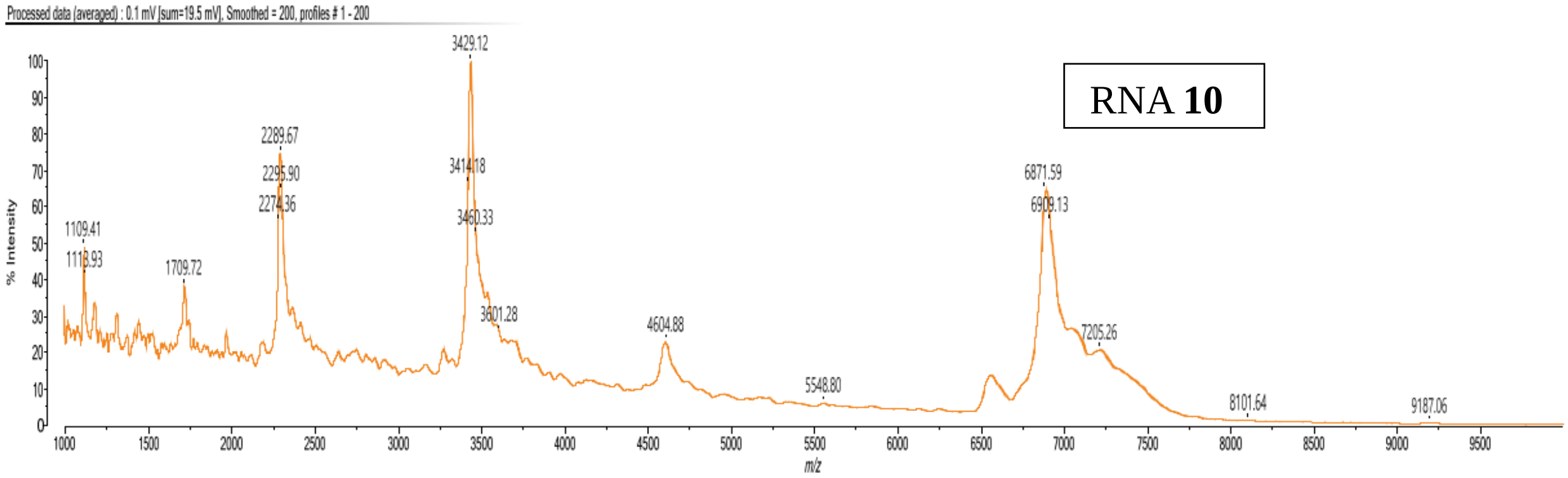
Processed data (averaged) : 0.1 mV [sum=19.5 mV], Smoothed = 200, profiles # 1 - 200
RNA 10
% Intensity
m/z
3429.12
2289.67
2295.90
2274.36
3414.18
3460.33
1109.41
1118.93
1709.72
3601.28
4604.88
6871.59
6909.13
7205.26
5548.80
8101.64
9187.06

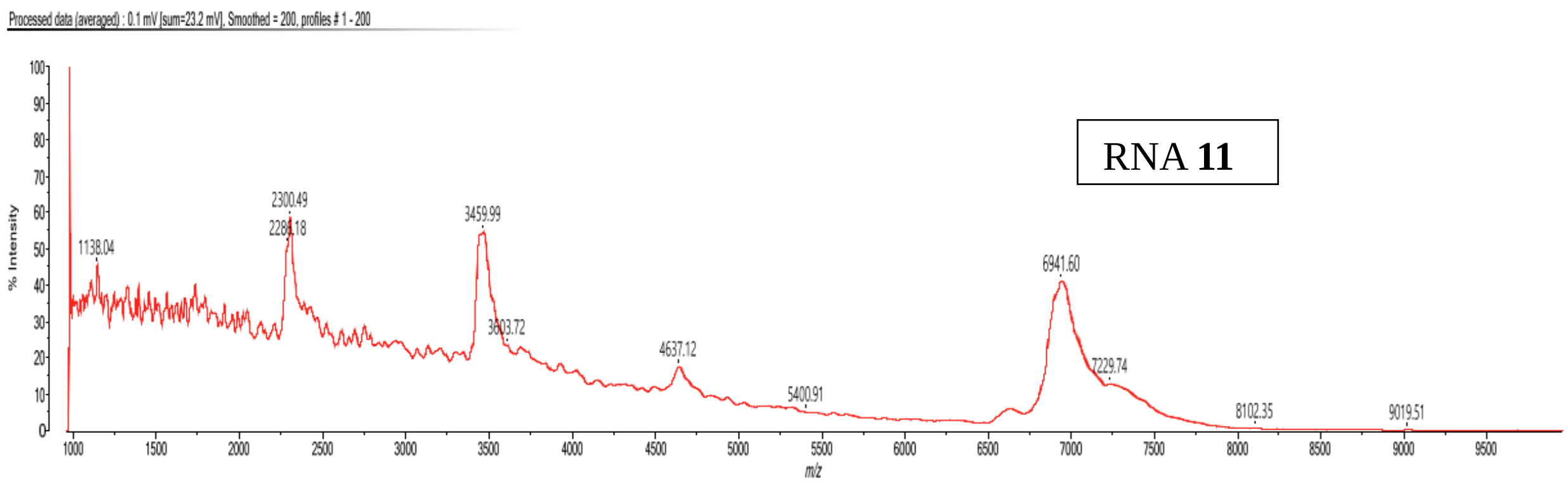
Processed data (averaged) : 0.1 mV [sum=23.2 mV], Smoothed = 200, profiles # 1 - 200
RNA 11
% Intensity
m/z
2300.49
2284.18
3459.99
1138.04
6941.60
3603.72
4637.12
7229.74
5400.91
8102.35
9019.51

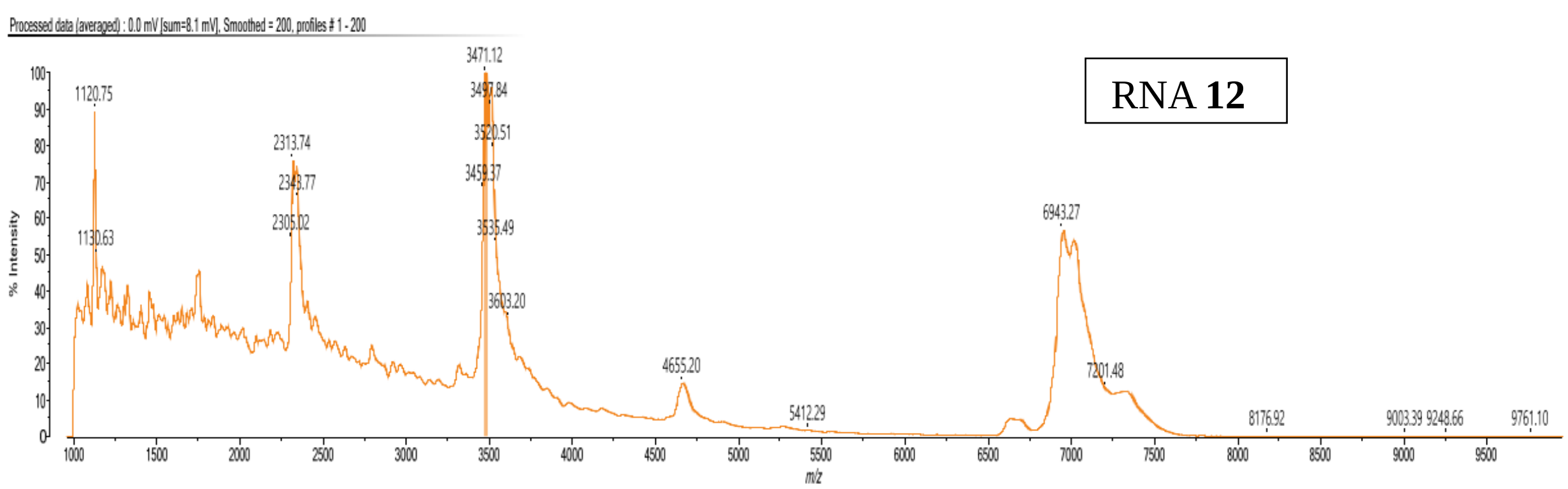
Processed data (averaged) : 0.0 mV [sum=8.1 mV], Smoothed = 200, profiles # 1 - 200
RNA 12
% Intensity
m/z
3471.12
3497.84
3520.51
3459.37
3535.49
3603.20
1120.75
1130.63
2313.74
2343.77
2305.02
6943.27
7201.48
4655.20
5412.29
8176.92
9003.39 9248.66
9761.10

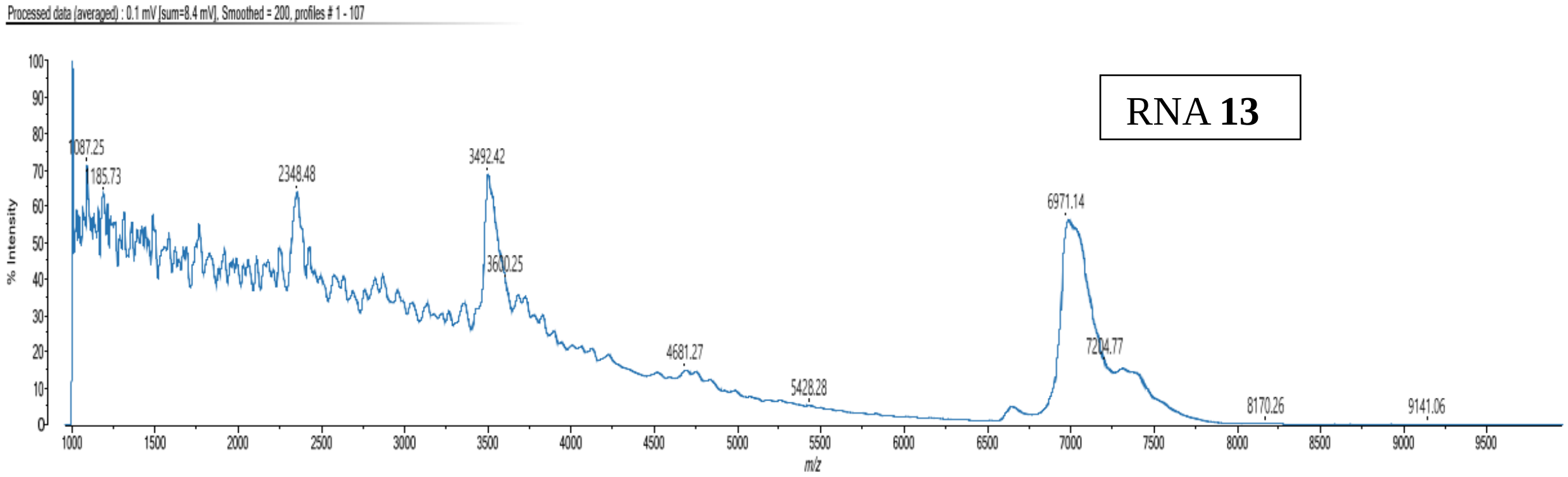
Processed data (averaged) : 0.1 mV [sum=8.4 mV], Smoothed = 200, profiles # 1 - 107
RNA 13
% Intensity
m/z

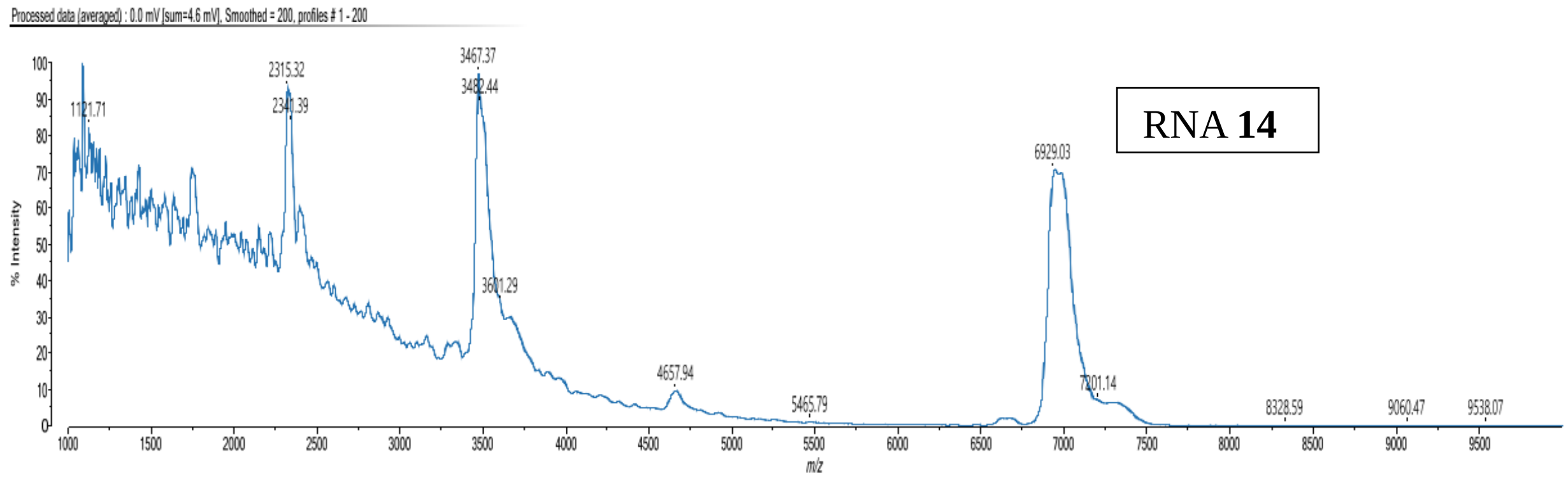
Processed data (averaged) : 0.0 mV [sum=4.6 mV], Smoothed = 200, profiles # 1 - 200
RNA 14
% Intensity
m/z

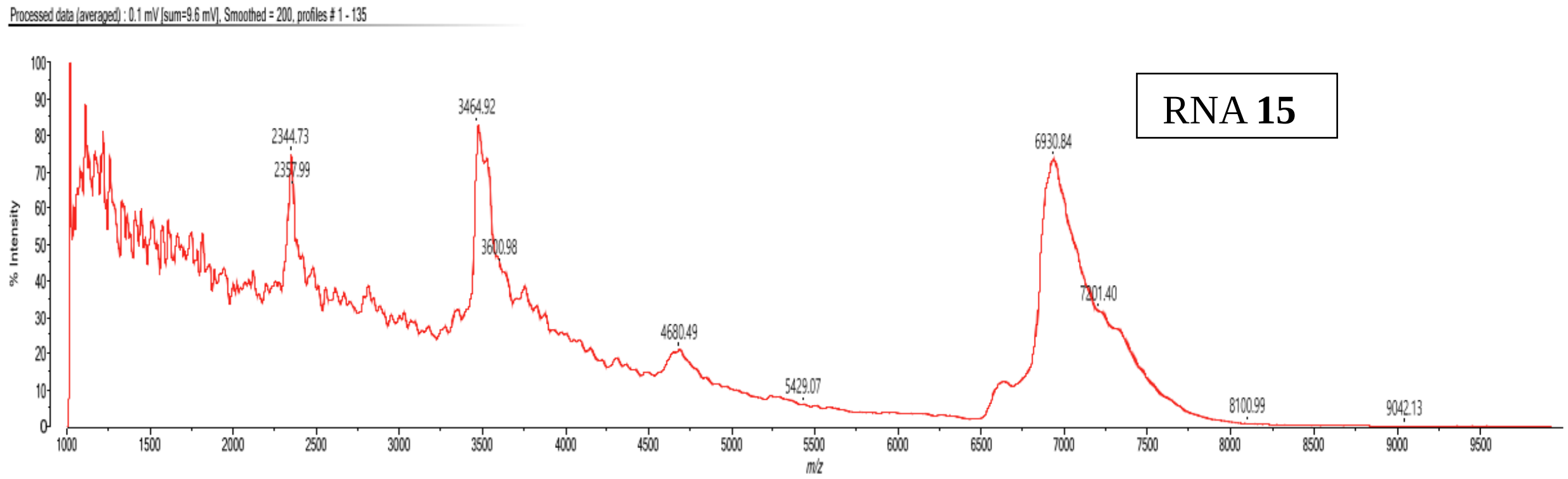
Processed data (averaged) : 0.1 mV [sum=9.6 mV], Smoothed = 200, profiles # 1 - 135
RNA 15
% Intensity
m/z

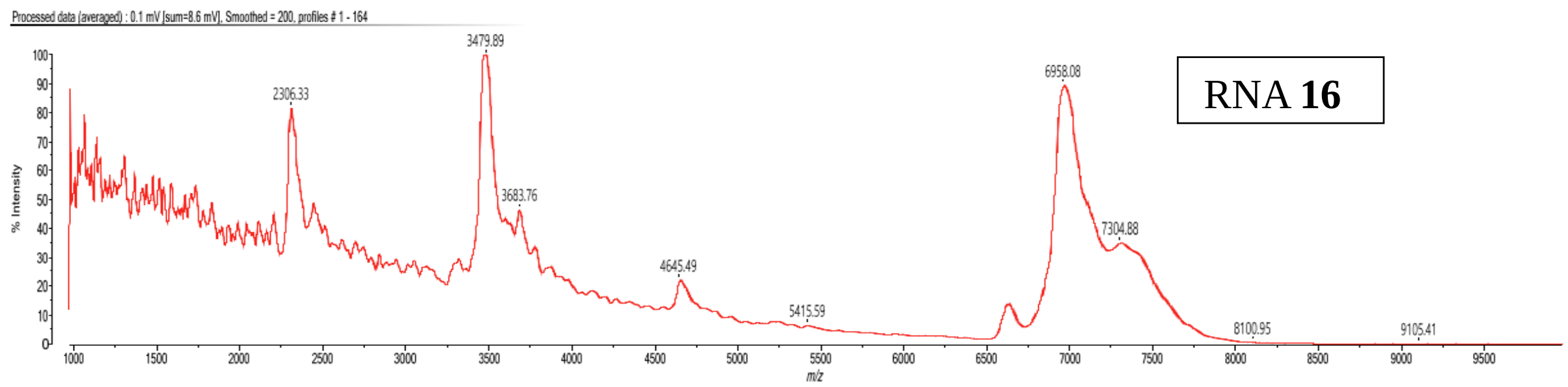

RNA **16**

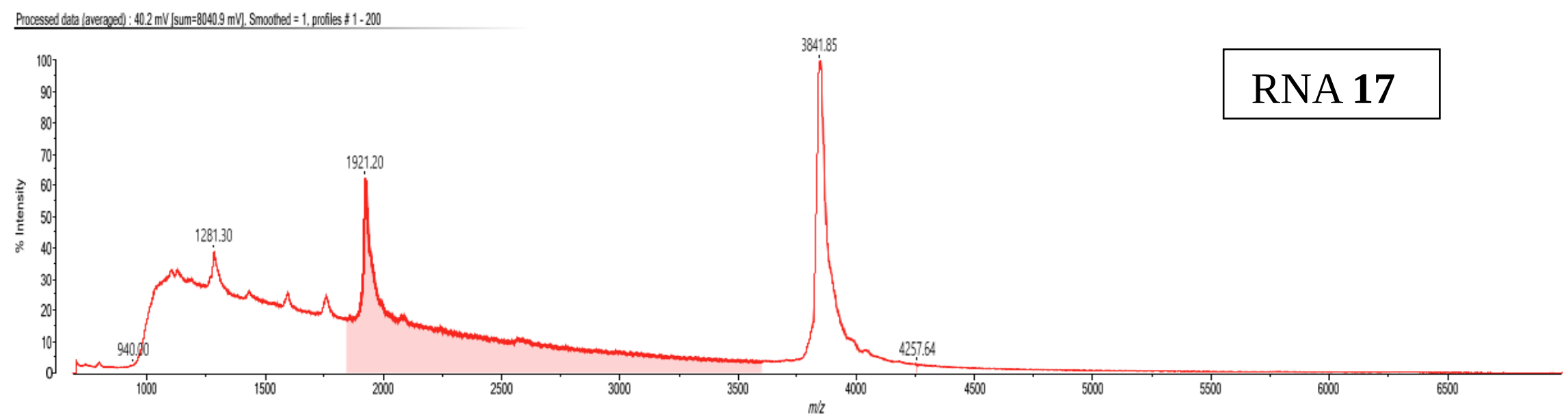

RNA **17**

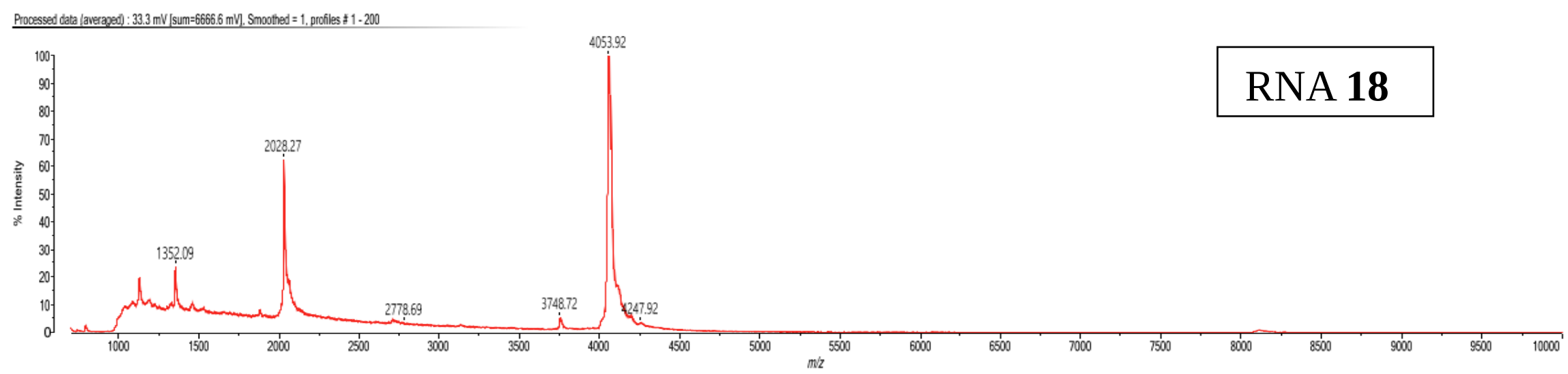

RNA **18**

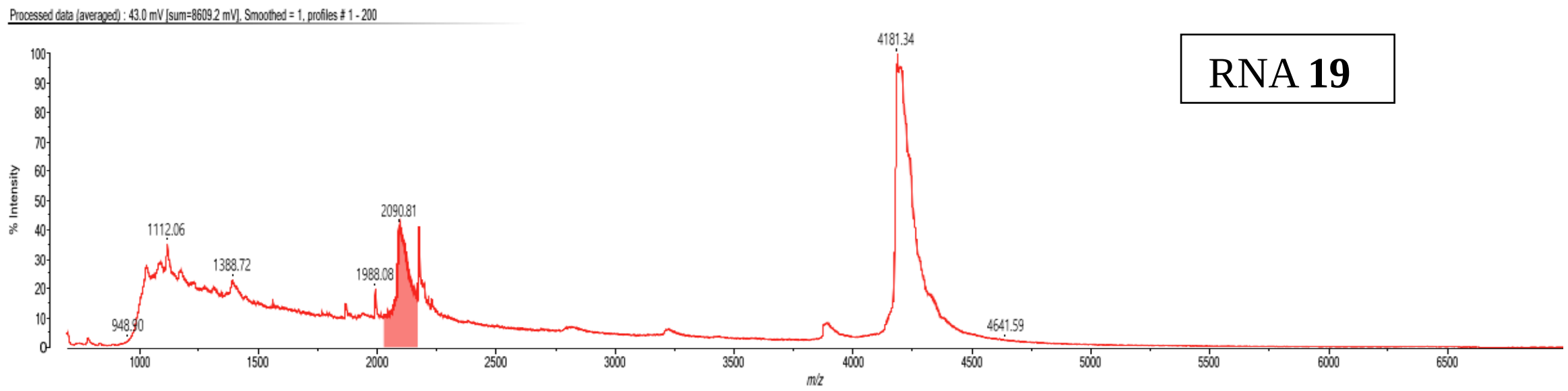

Processed data (averaged) : 43.0 mV [sum=8609.2 mV], Smoothed = 1, profiles # 1 - 200
RNA 19
4181.34
2090.81
1112.06
1388.72
1988.08
948.90
4641.59
% Intensity
m/z

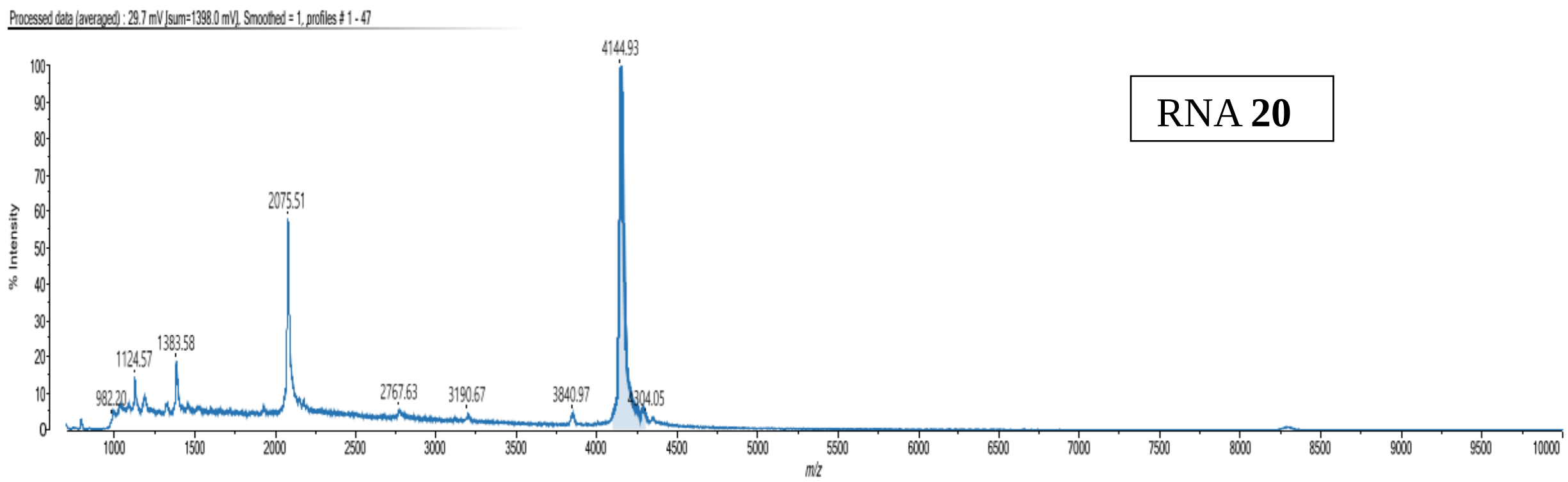

Processed data (averaged) : 29.7 mV [sum=1398.0 mV], Smoothed = 1, profiles # 1 - 47
RNA 20
4144.93
2075.51
1383.58
1124.57
982.20
2767.63
3190.67
3840.97
% Intensity
m/z

3. Figure S2. HPLC Characterization of ssRNAs **1–20**

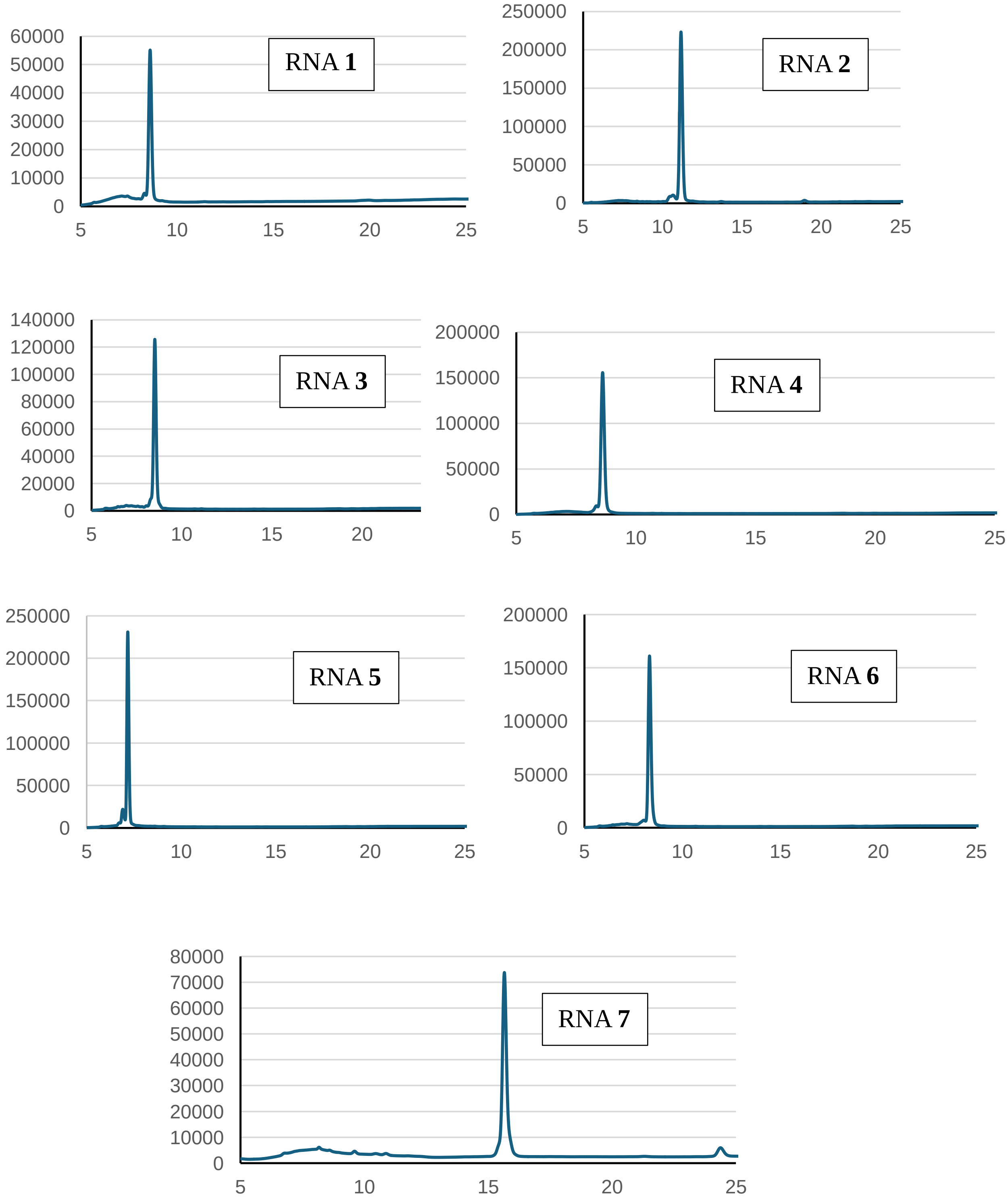

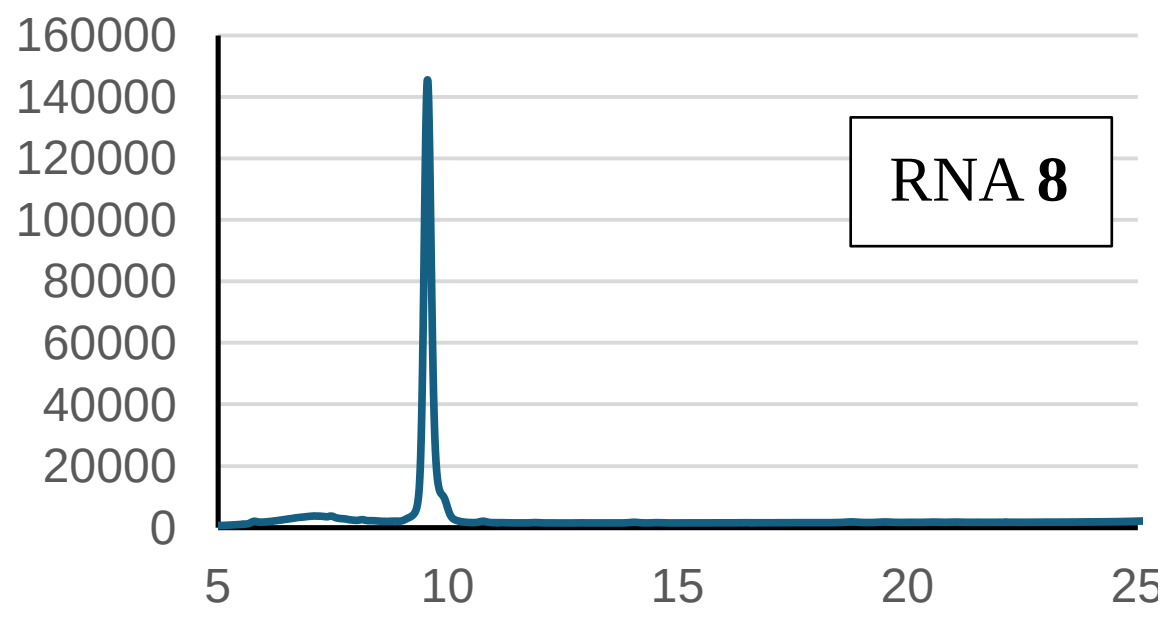
RNA 8
160000
140000
120000
100000
80000
60000
40000
20000
0
5
10
15
20
25

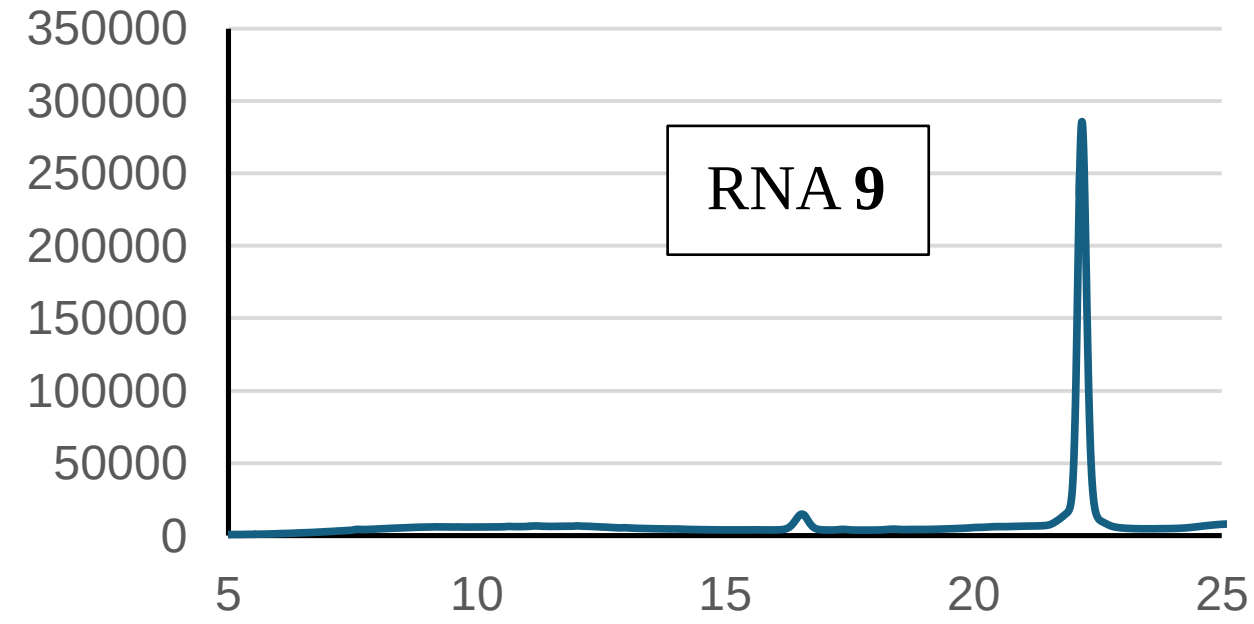
RNA 9
350000
300000
250000
200000
150000
100000
50000
0
5
10
15
20
25

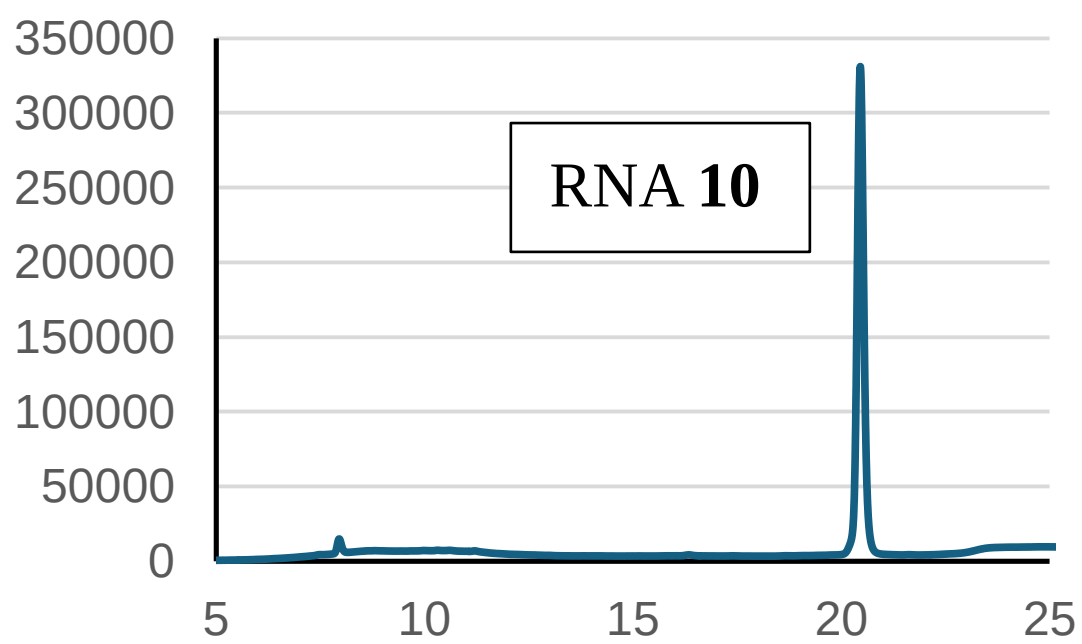
RNA 10
350000
300000
250000
200000
150000
100000
50000
0
5
10
15
20
25

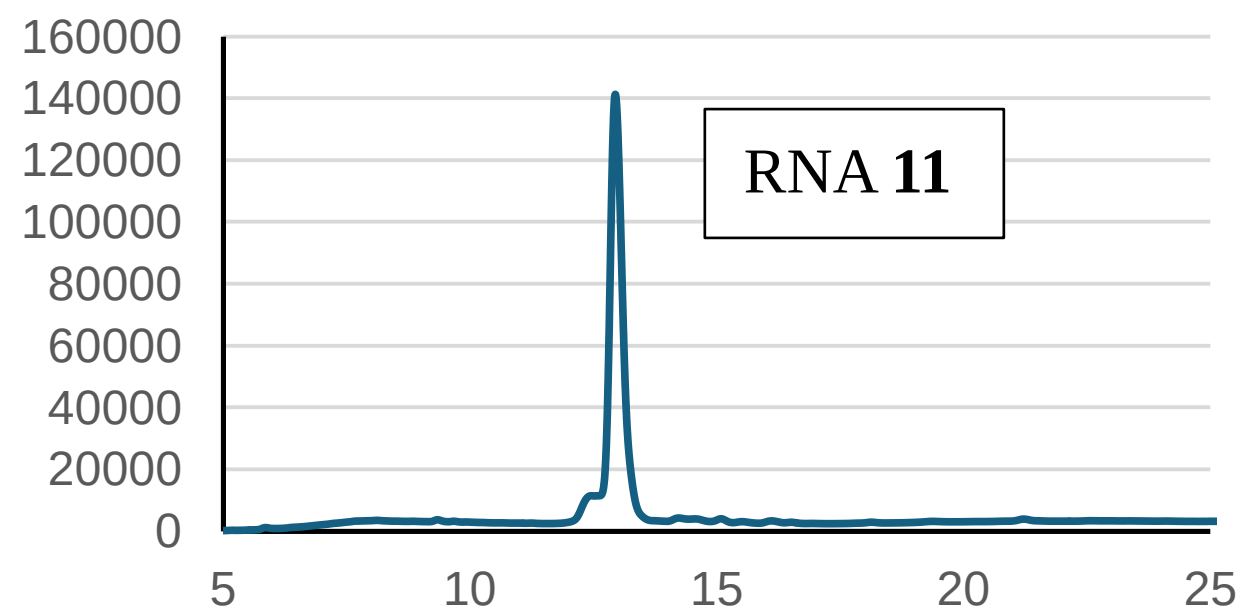
RNA 11
160000
140000
120000
100000
80000
60000
40000
20000
0
5
10
15
20
25

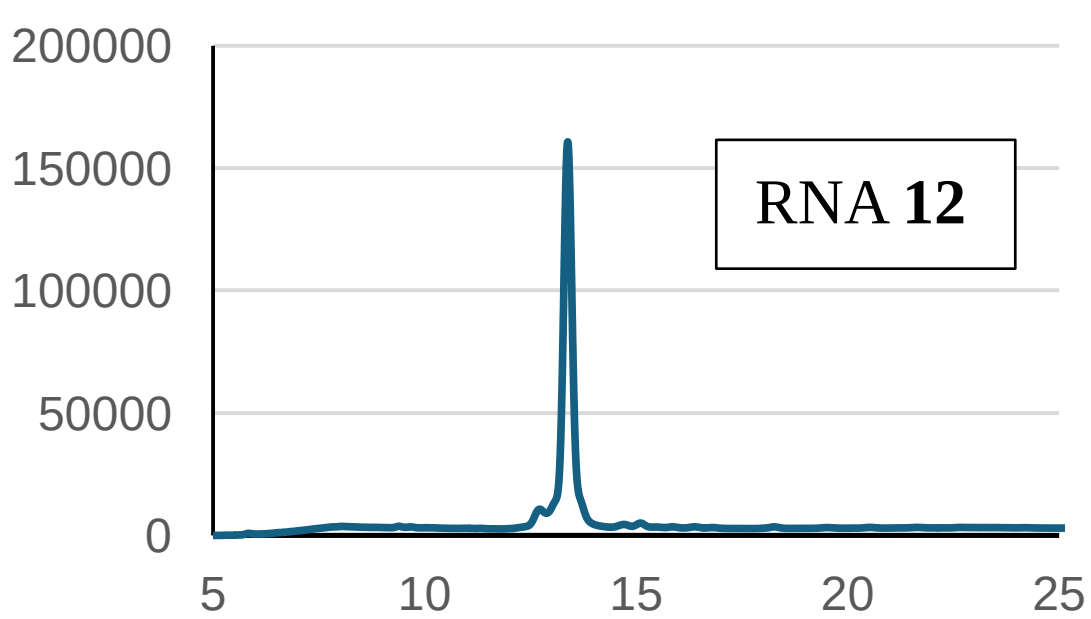
RNA 12
200000
150000
100000
50000
0
5
10
15
20
25

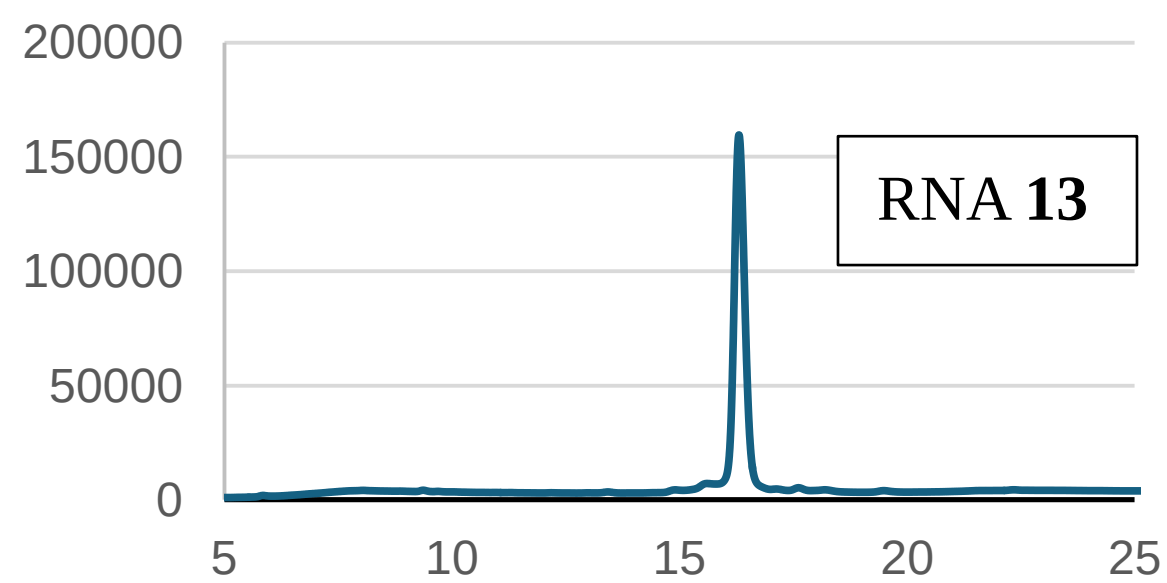
RNA 13
200000
150000
100000
50000
0
5
10
15
20
25

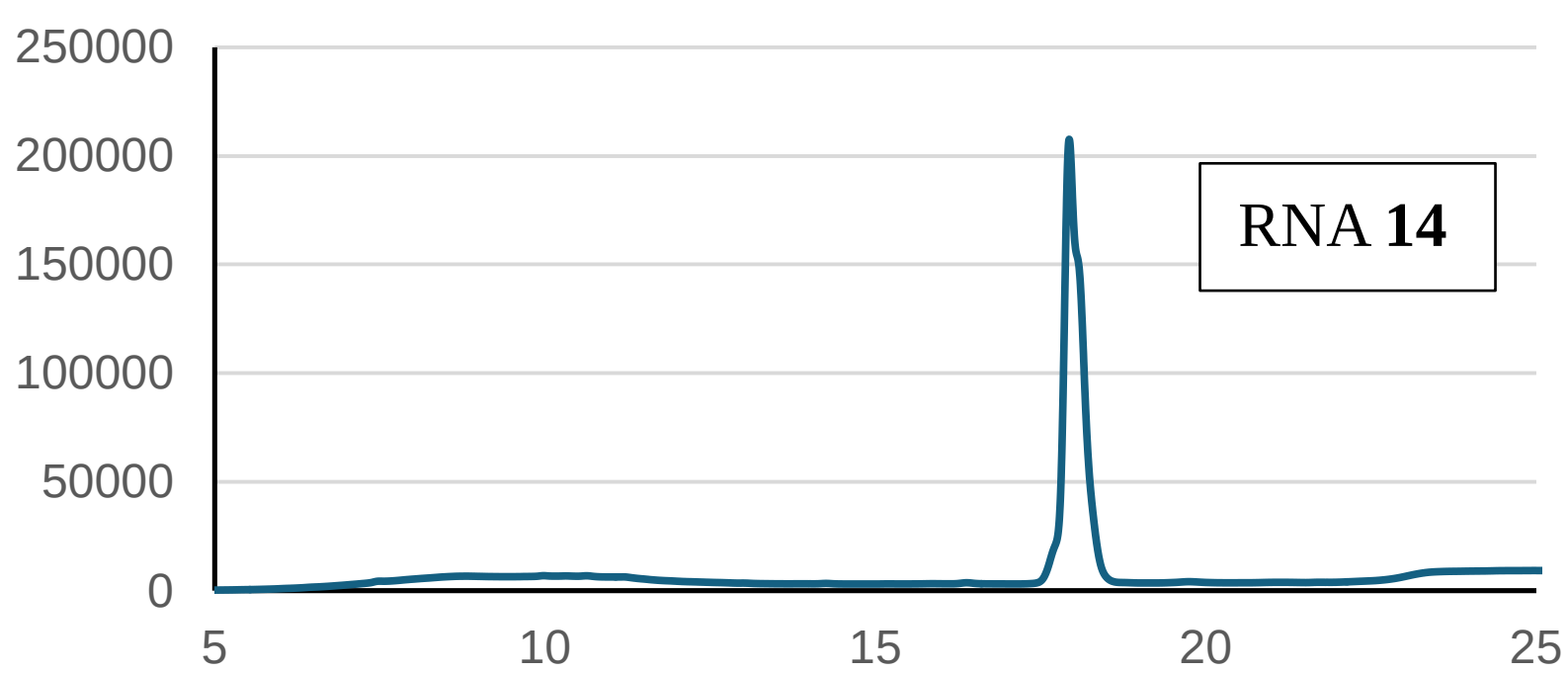
RNA 14
250000
200000
150000
100000
50000
0
5
10
15
20
25

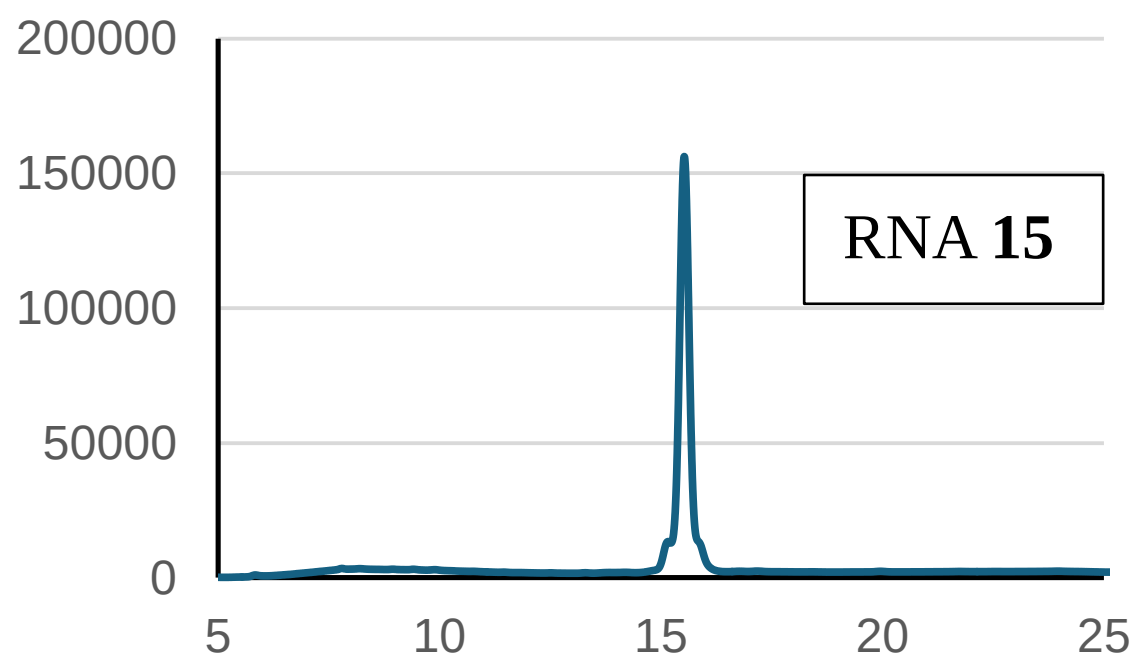
RNA **15**
200000
150000
100000
50000
0
5
10
15
20
25

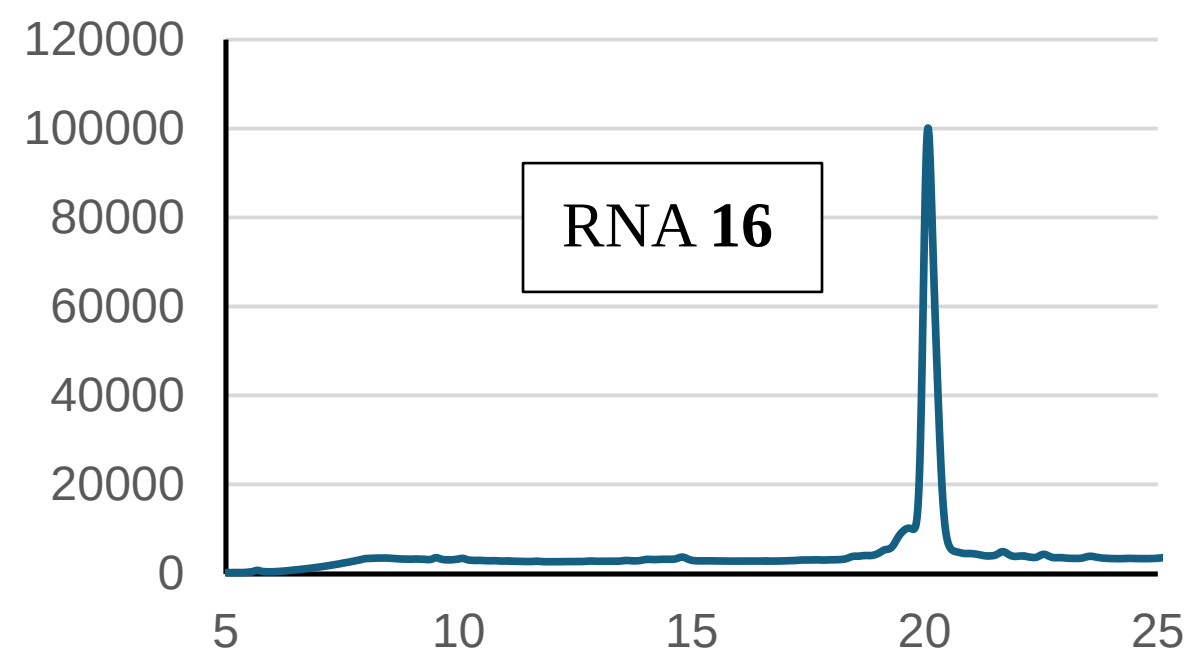
RNA **16**
120000
100000
80000
60000
40000
20000
0
5
10
15
20
25

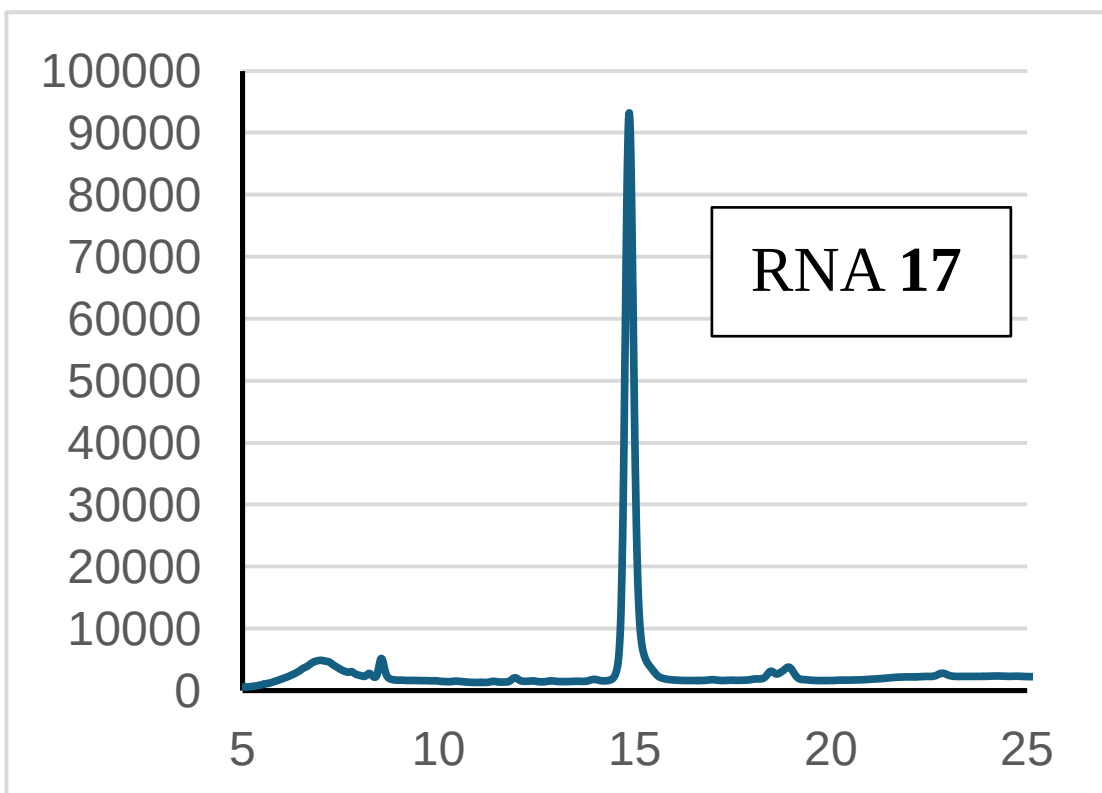
RNA **17**
100000
90000
80000
70000
60000
50000
40000
30000
20000
10000
0
5
10
15
20
25

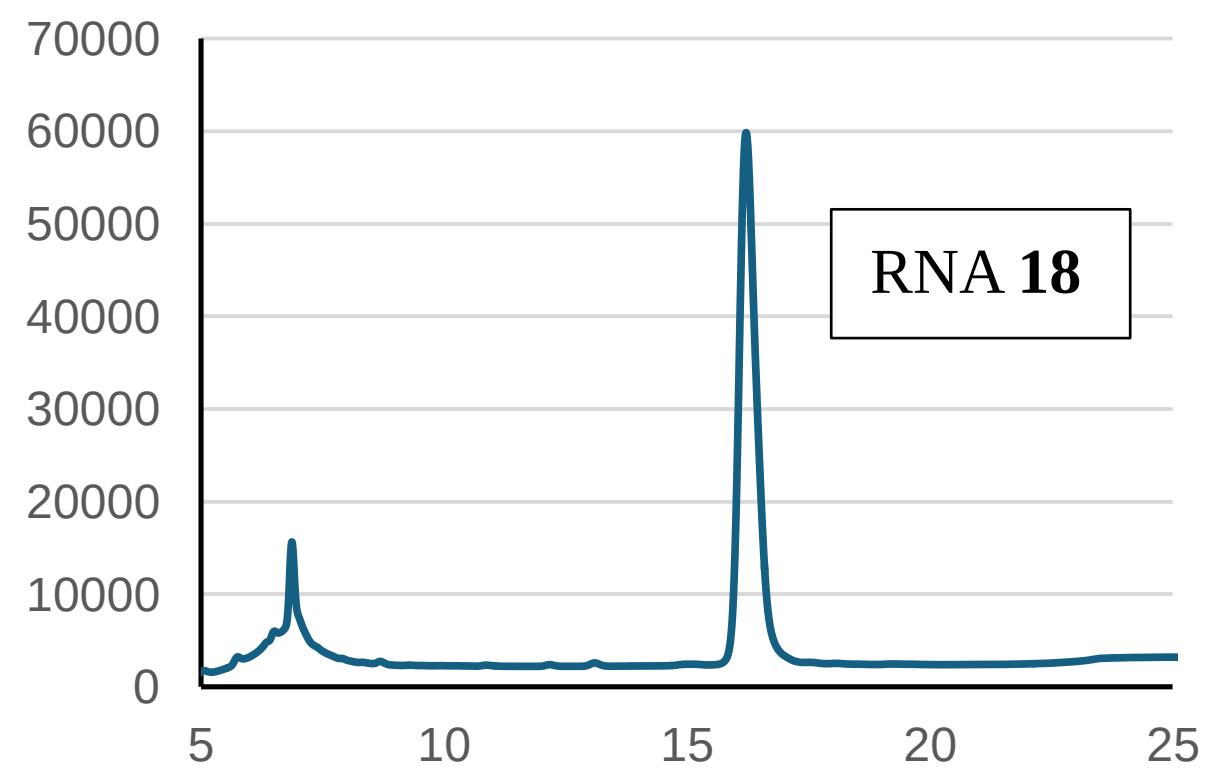
RNA **18**
70000
60000
50000
40000
30000
20000
10000
0
5
10
15
20
25

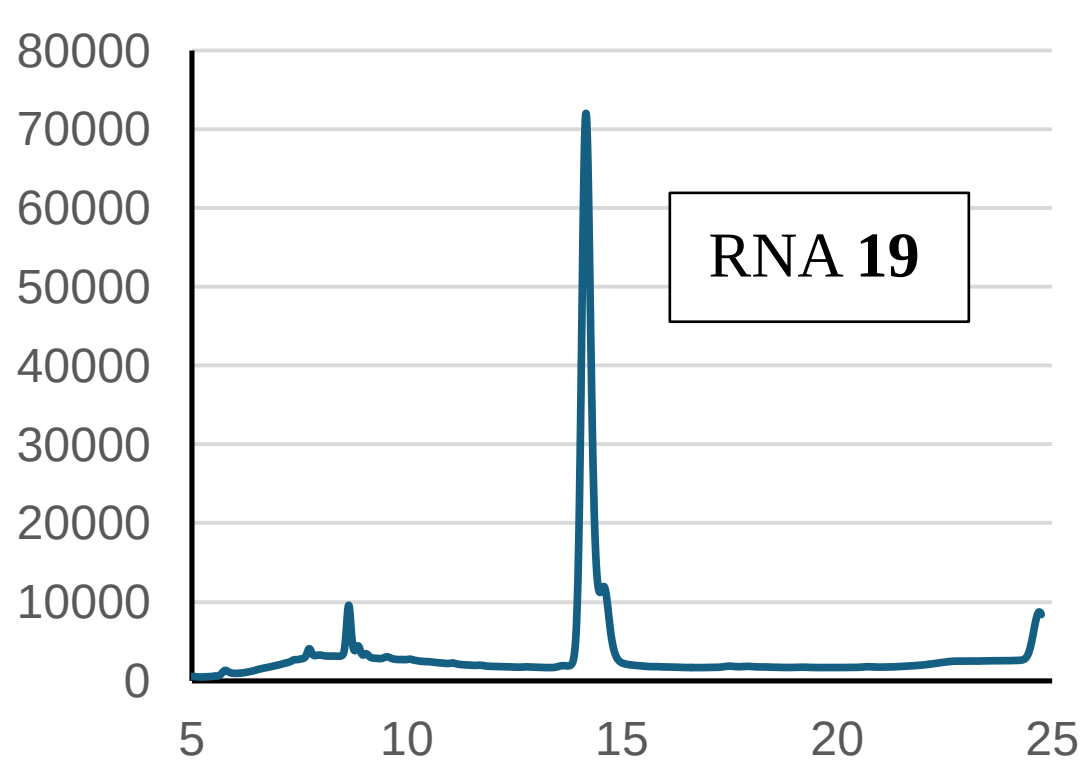
RNA **19**
80000
70000
60000
50000
40000
30000
20000
10000
0
5
10
15
20
25

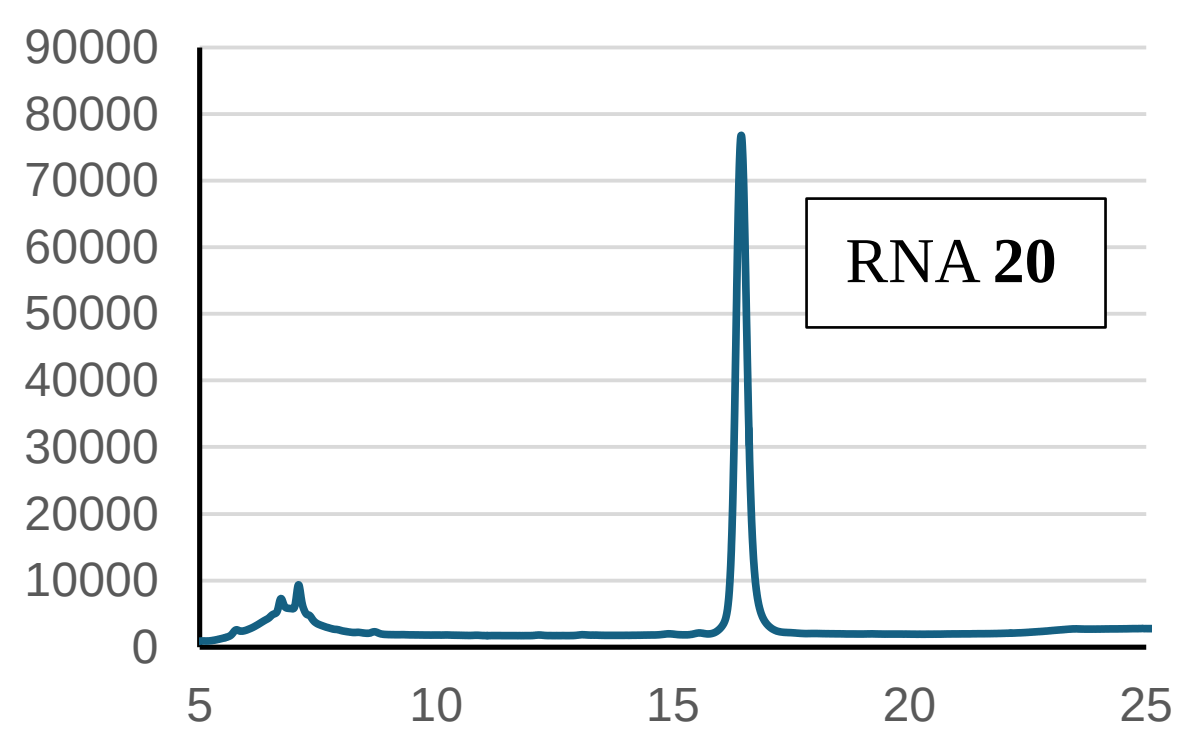
RNA **20**
90000
80000
70000
60000
50000
40000
30000
20000
10000
0
5
10
15
20
25

4. Figure S3. UV Melting Profiles of Modified and Unmodified dsRNAs (**1**–**7**) and siRNAs (**1**–**7**) Incorporating Different Modification Designs

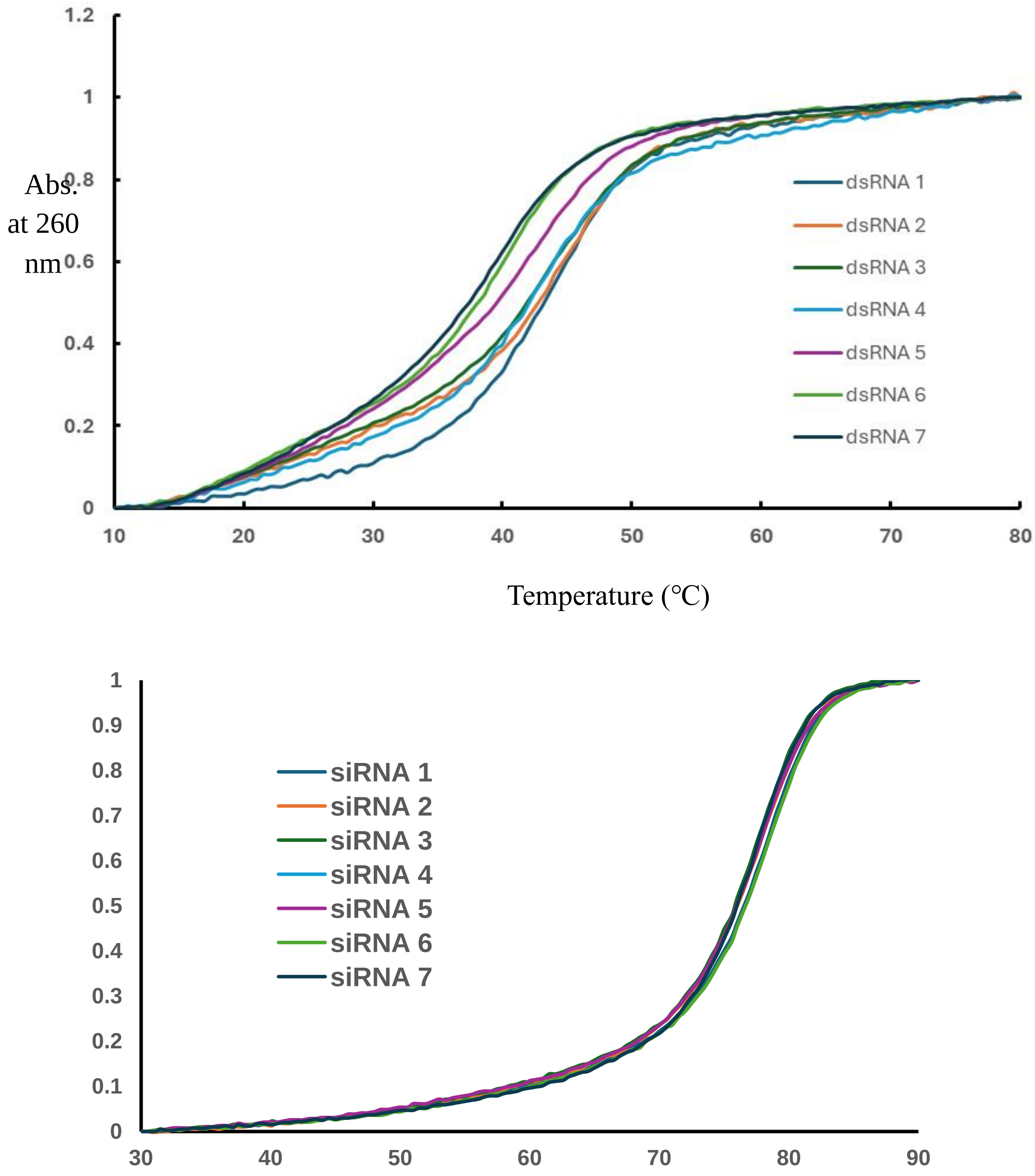

5. Figure S4. UV Melting Curves of dsRNAs at Various Concentrations for Thermodynamic Analysis: (**a**, **b**) Native Duplex, (**c**, **d**) Modified with (*S*)-*C*-5'-Guanidinopropyl-2'-*O*-Methyluridine, and (**e**, **f**) Modified with (*S*)-*C*-5'-Guanidinopropyl-2'-Fluorouridine

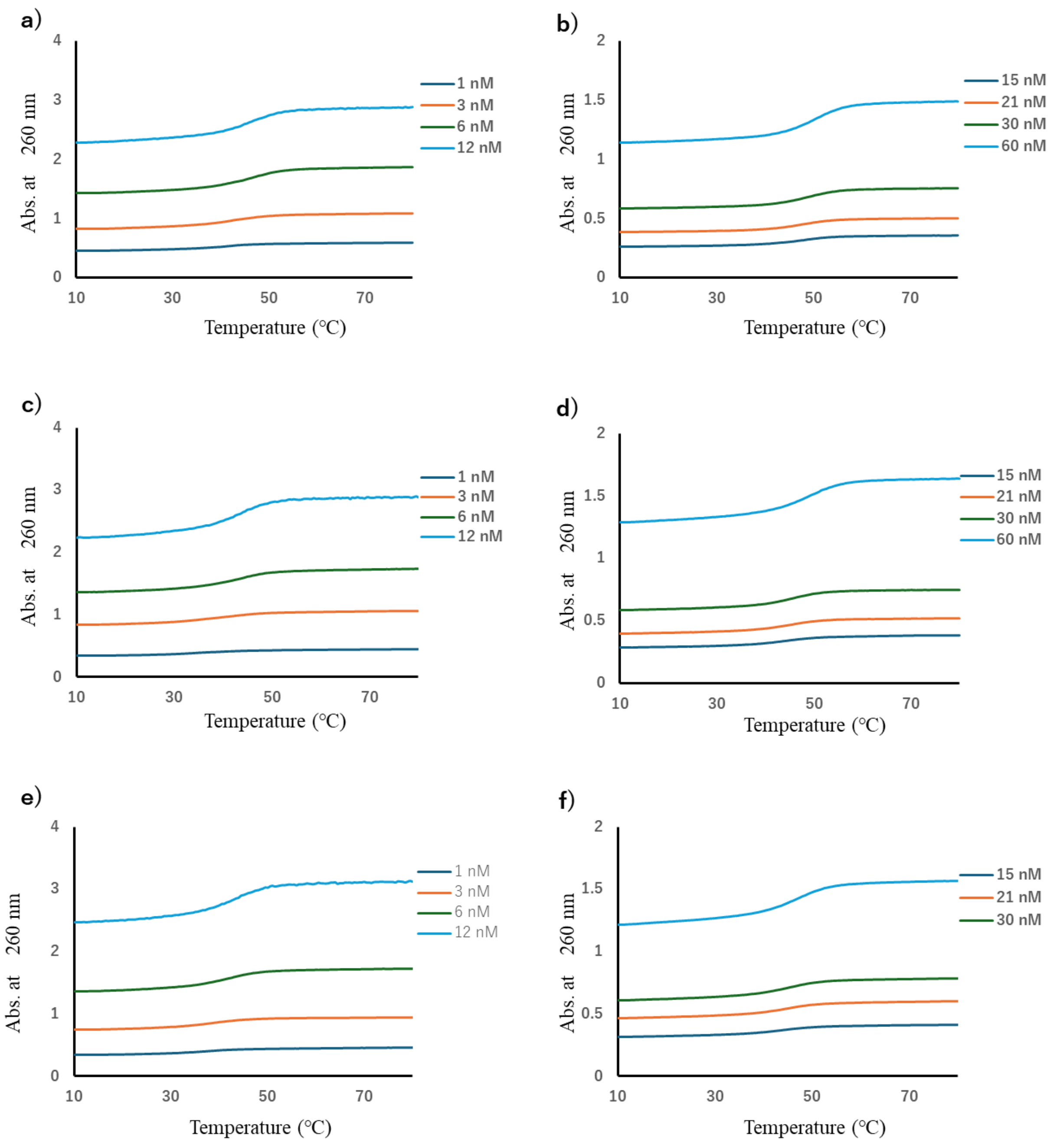

6. Figure S5. Plots of $1/T_m$ versus ln $(C_T/4)$ for dsRNAs **1**, **6**, and **7**

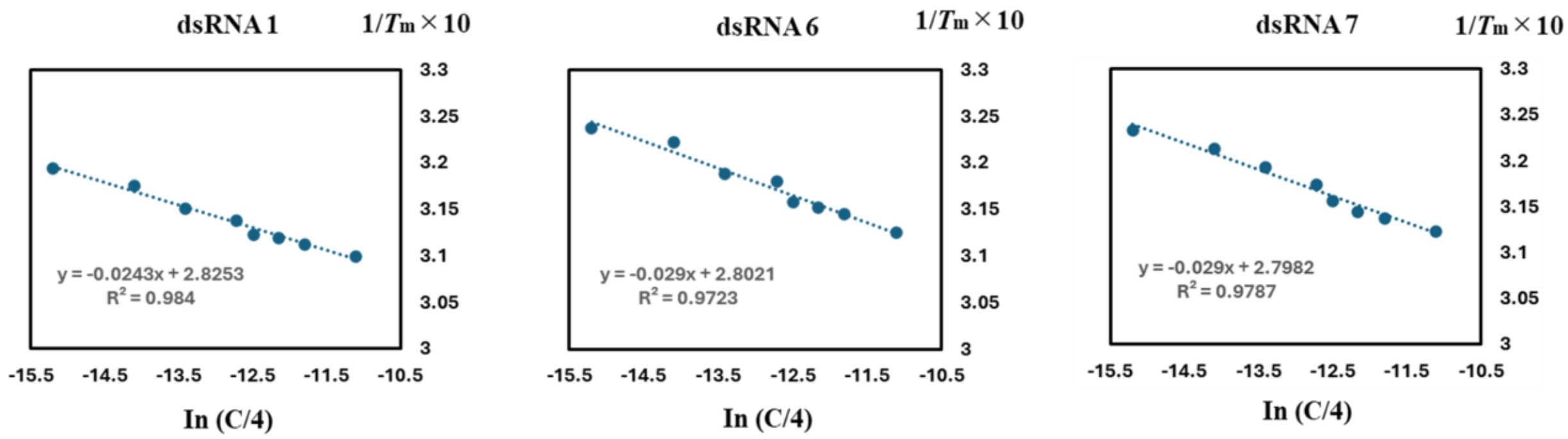

7. Figure S6. UV Melting Profiles of Modified and Unmodified dsRNAs Used for Base-Discrimination Analysis

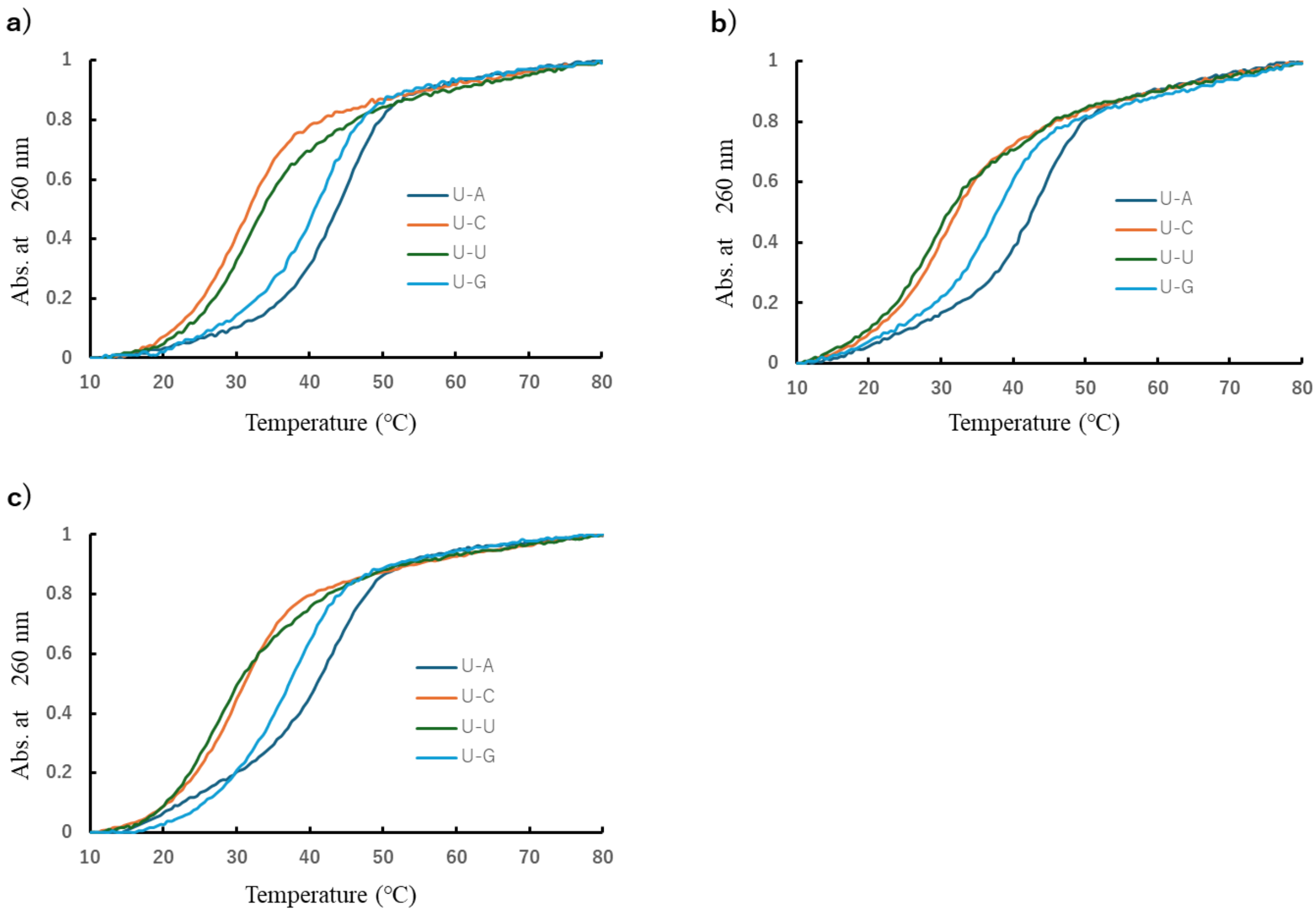

8. Figure S7. Circular Dichroism (CD) Spectra of *KNTC2*-Targeting siRNAs: CD spectra of both unmodified and modified *KNTC2*-targeting siRNAs (**1-7**) were recorded in a buffer containing 10 mM sodium phosphate (pH 7.0) and 100 nM NaCl at 15 °C. The concentration of the duplexes was 4 μM.

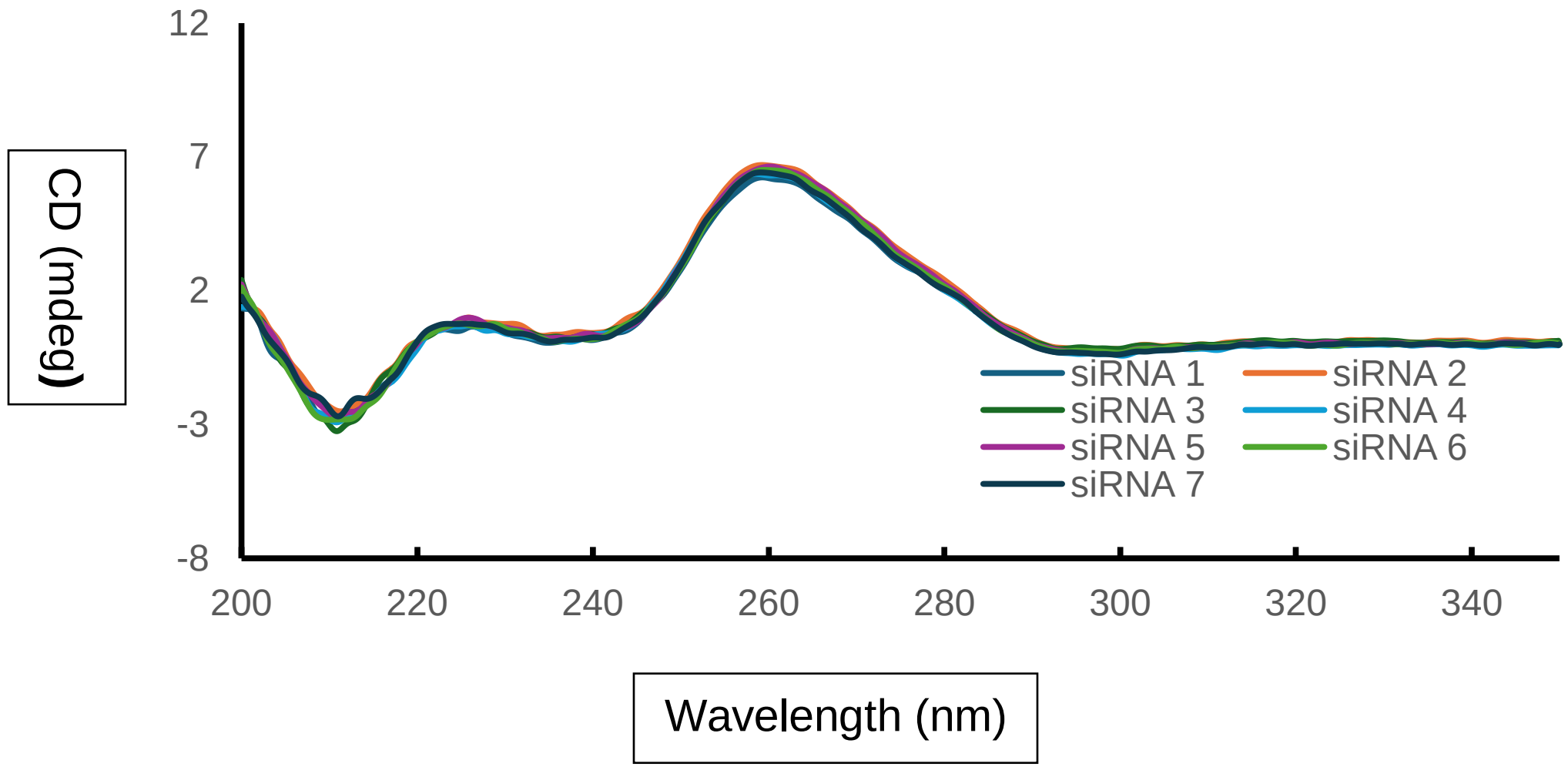

CD (mdeg)
12
7
2
-3
-8
200
220
240
260
280
300
320
340
siRNA 1
siRNA 2
siRNA 3
siRNA 4
siRNA 5
siRNA 6
siRNA 7
Wavelength (nm)

9. Copies of NMR spectra (^{1}H, ^{13}C, and ^{31}P)

^{1}HNMR (400 MHz, $CDCl_3$) **11a**

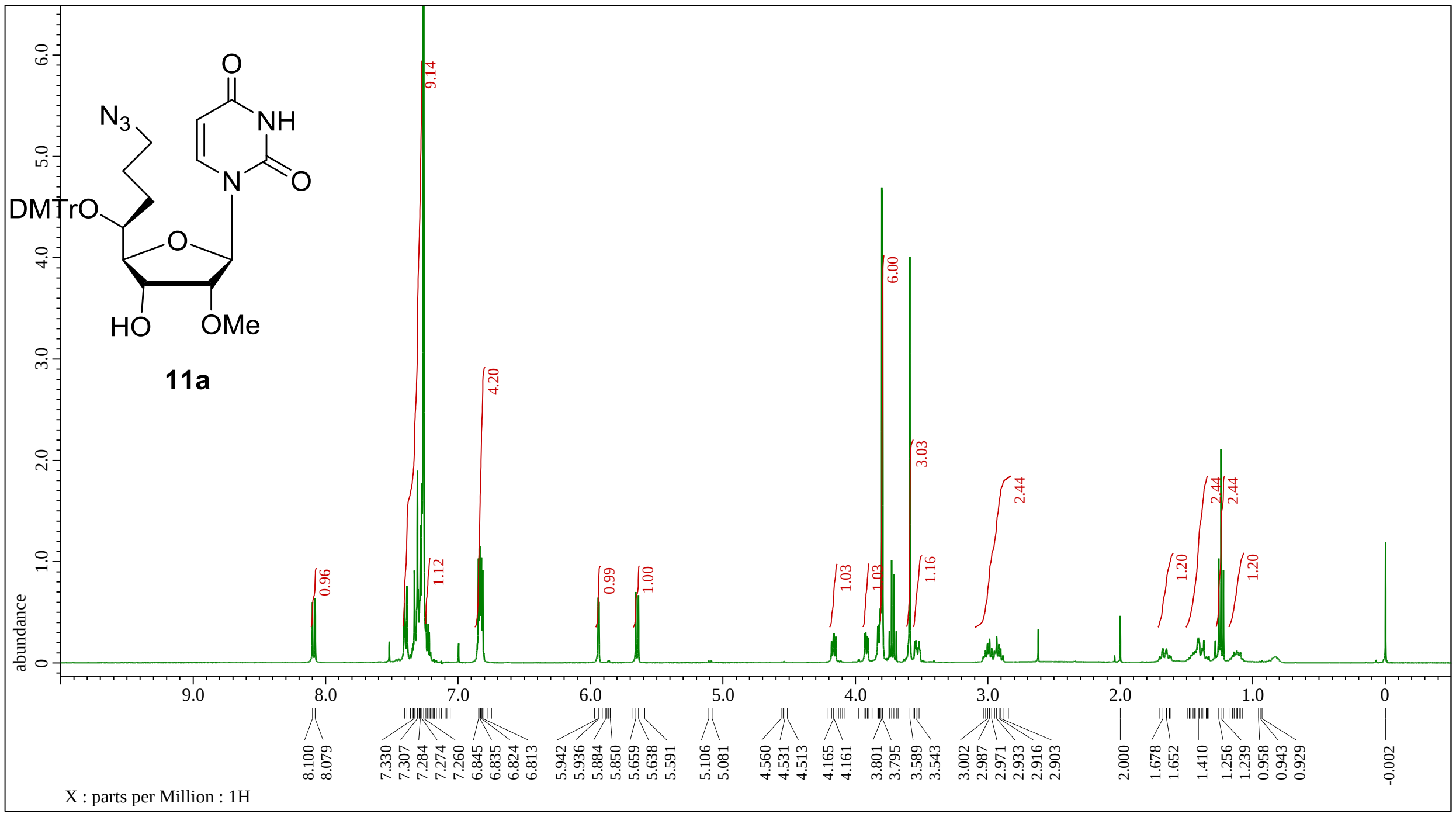

^{13}CNMR (101 MHz, $CDCl_3$) **11a**

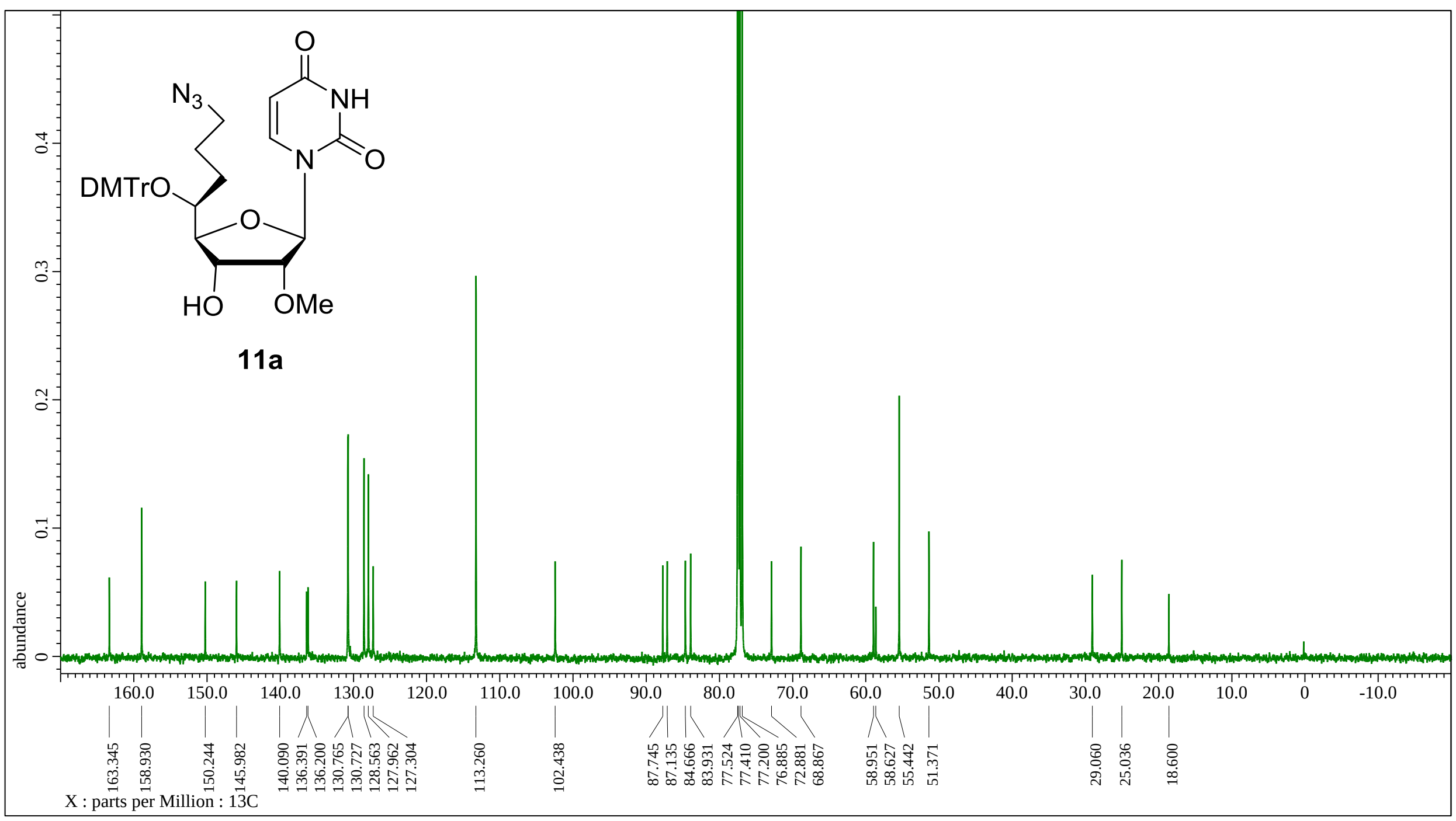

^{1}HNMR (400 MHz, $CDCl_3$) **11b**

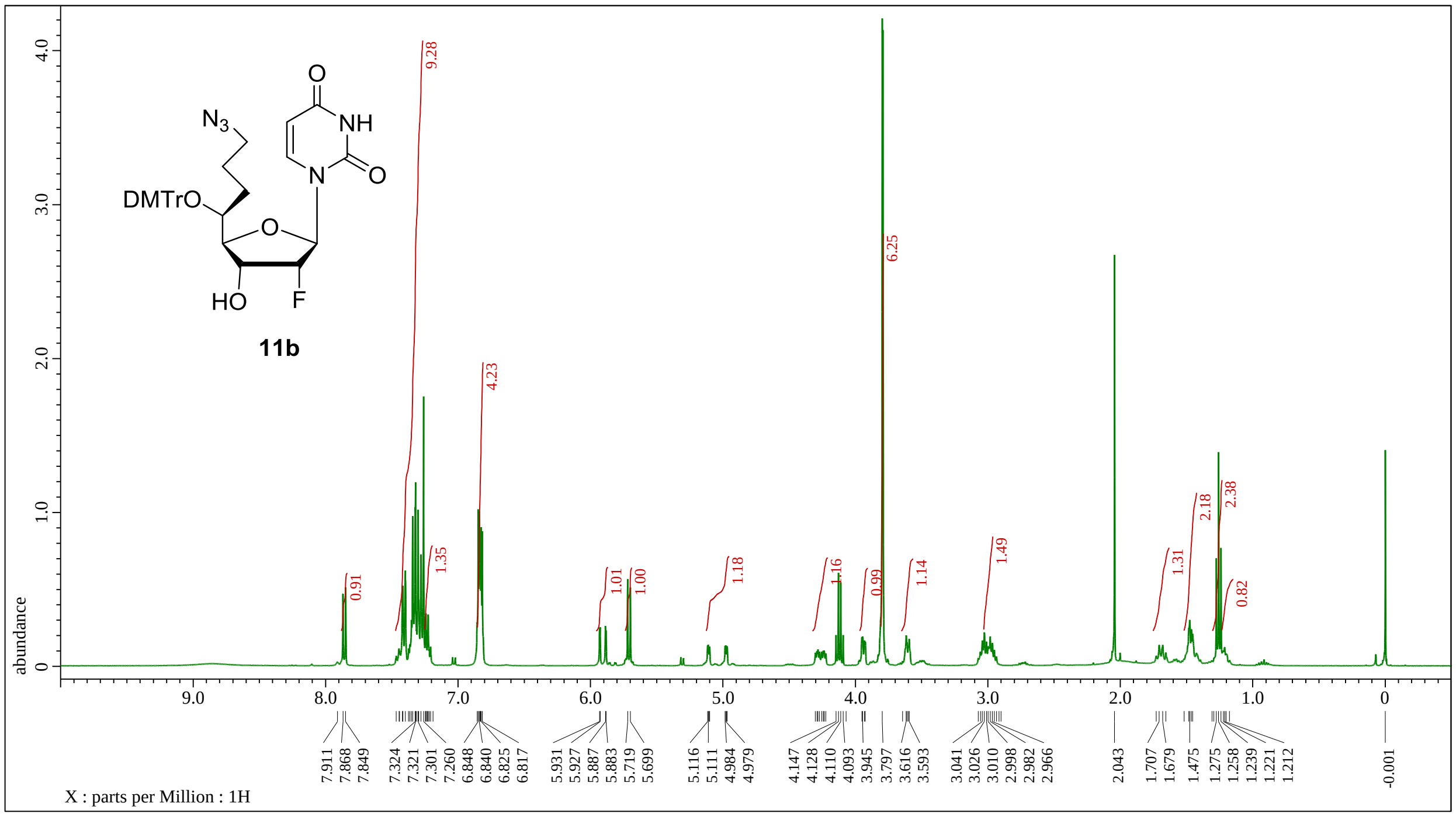

^{13}CNMR (101 MHz, $CDCl_3$) **11b**

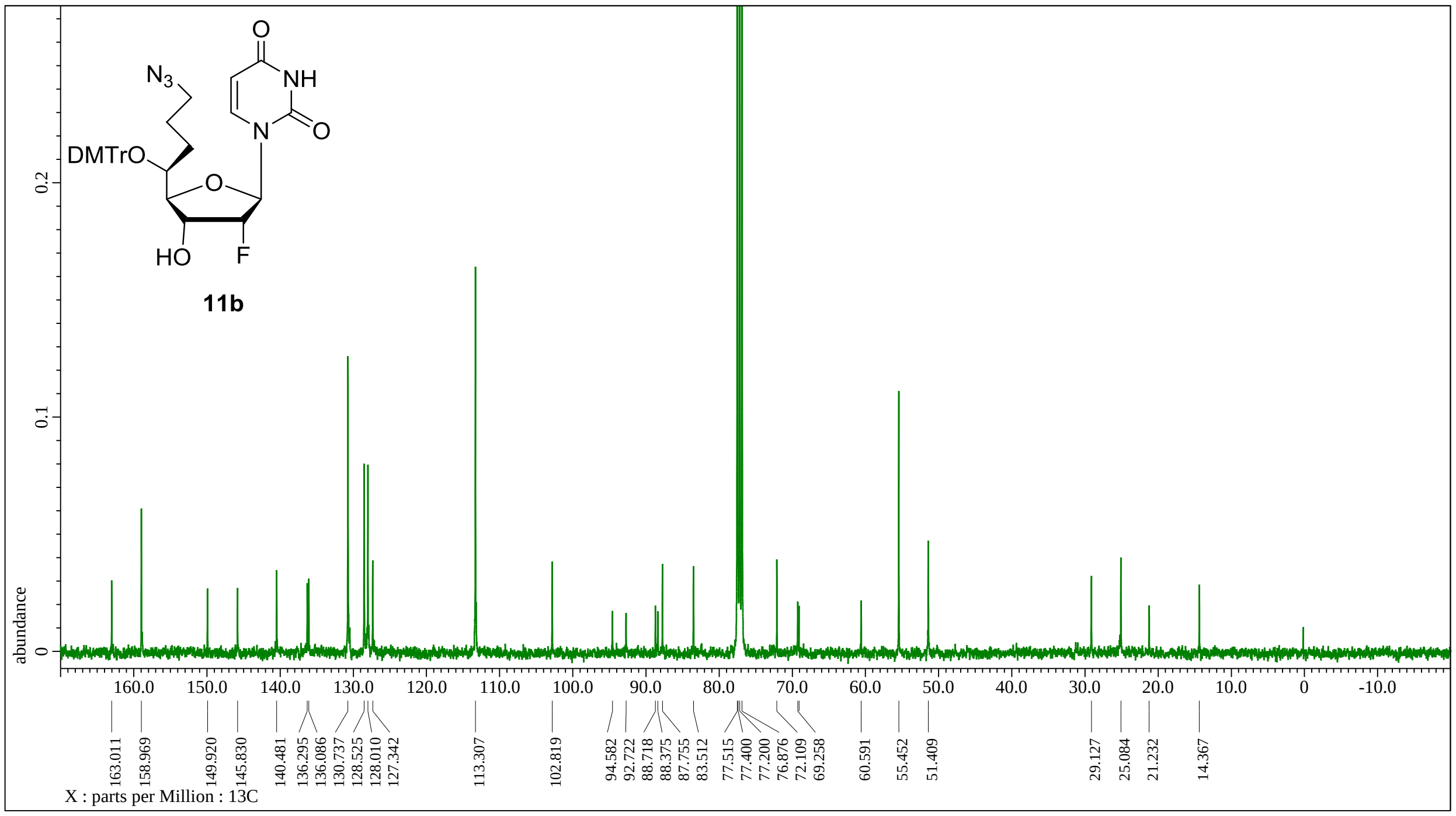

^{1}HNMR (400 MHz, $CDCl_3$) **13a**

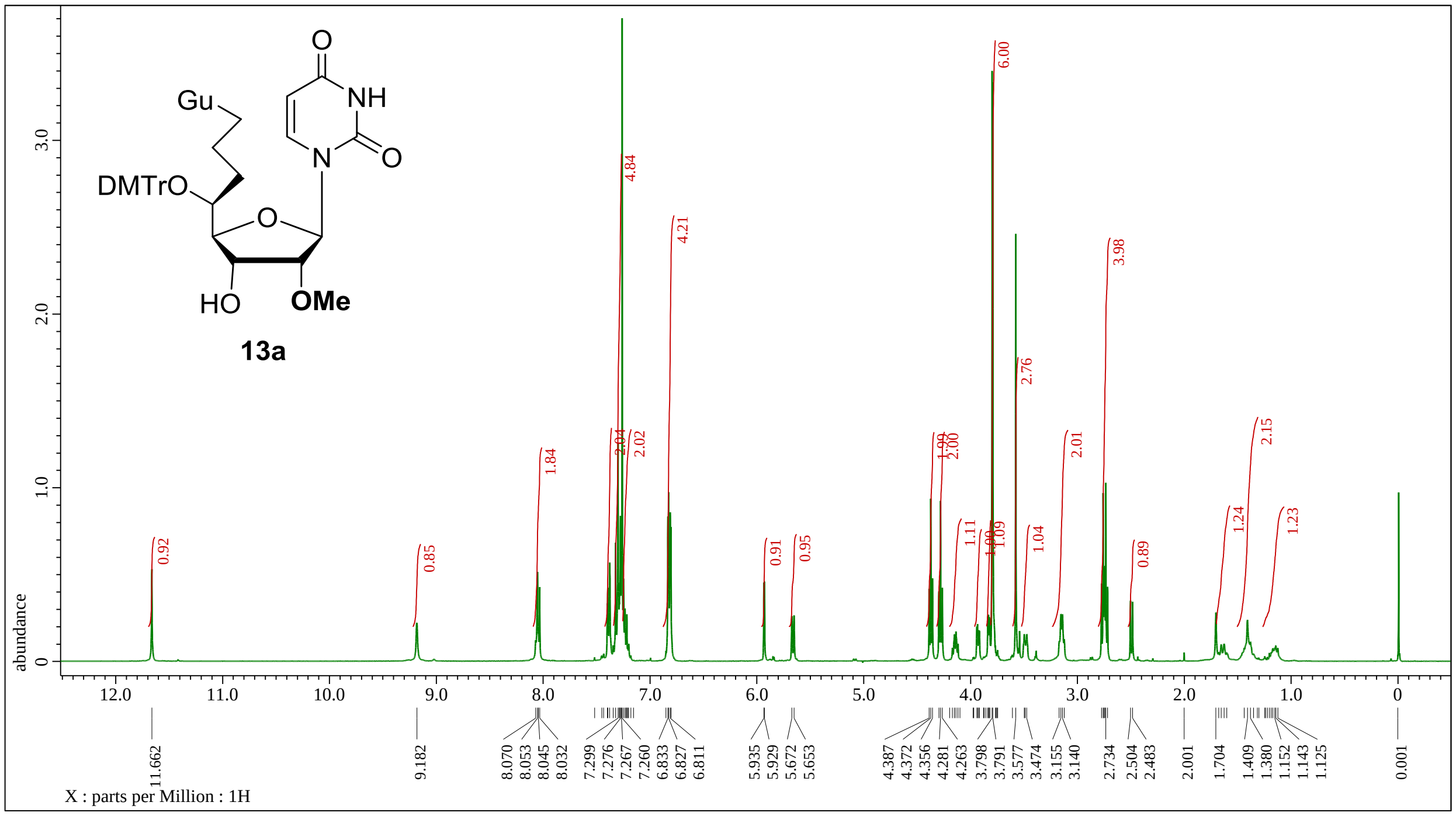

^{13}CNMR (101 MHz, $CDCl_3$) **13a**

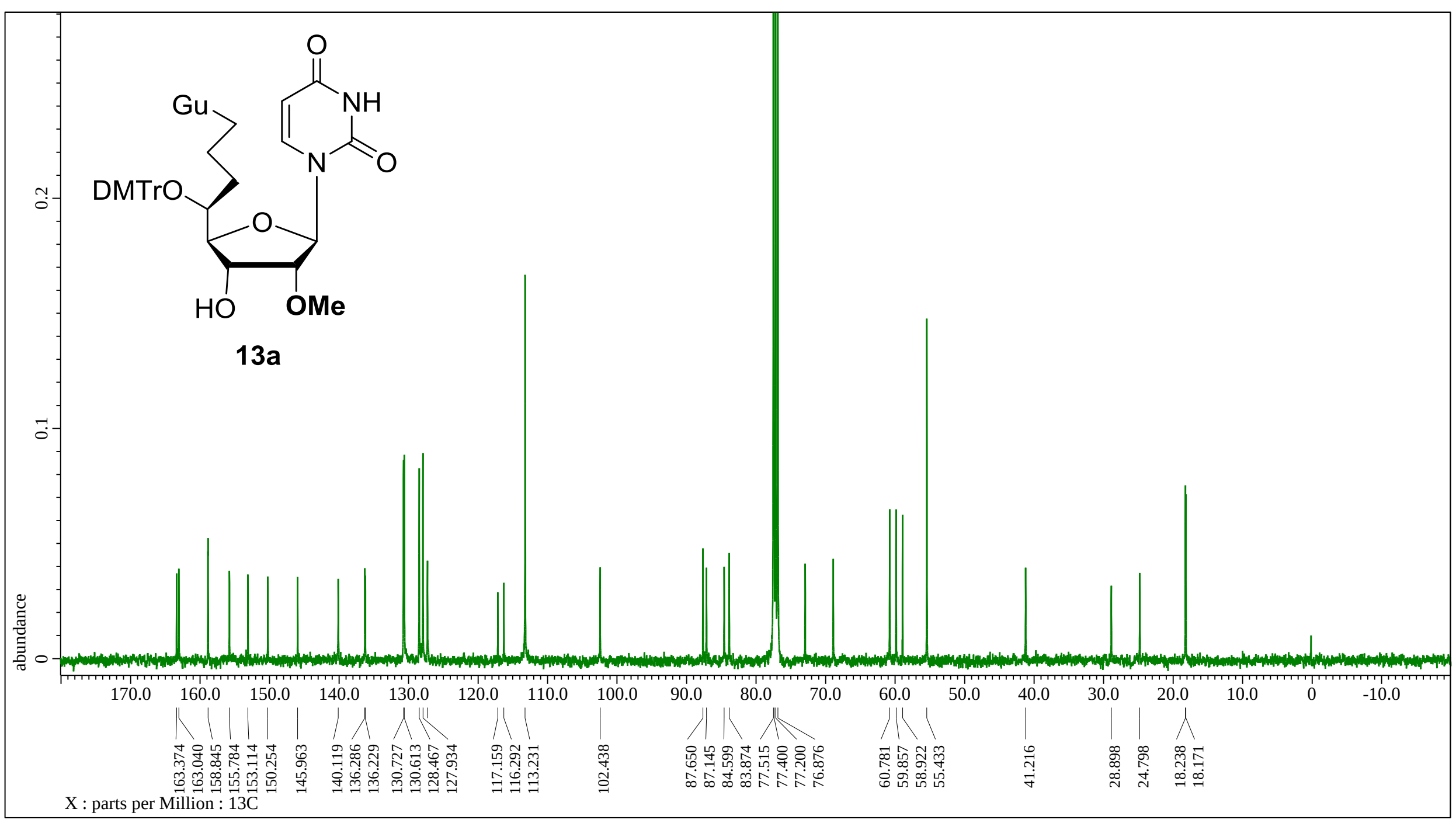

^{1}HNMR (400 MHz, $CDCl_3$) **13b**

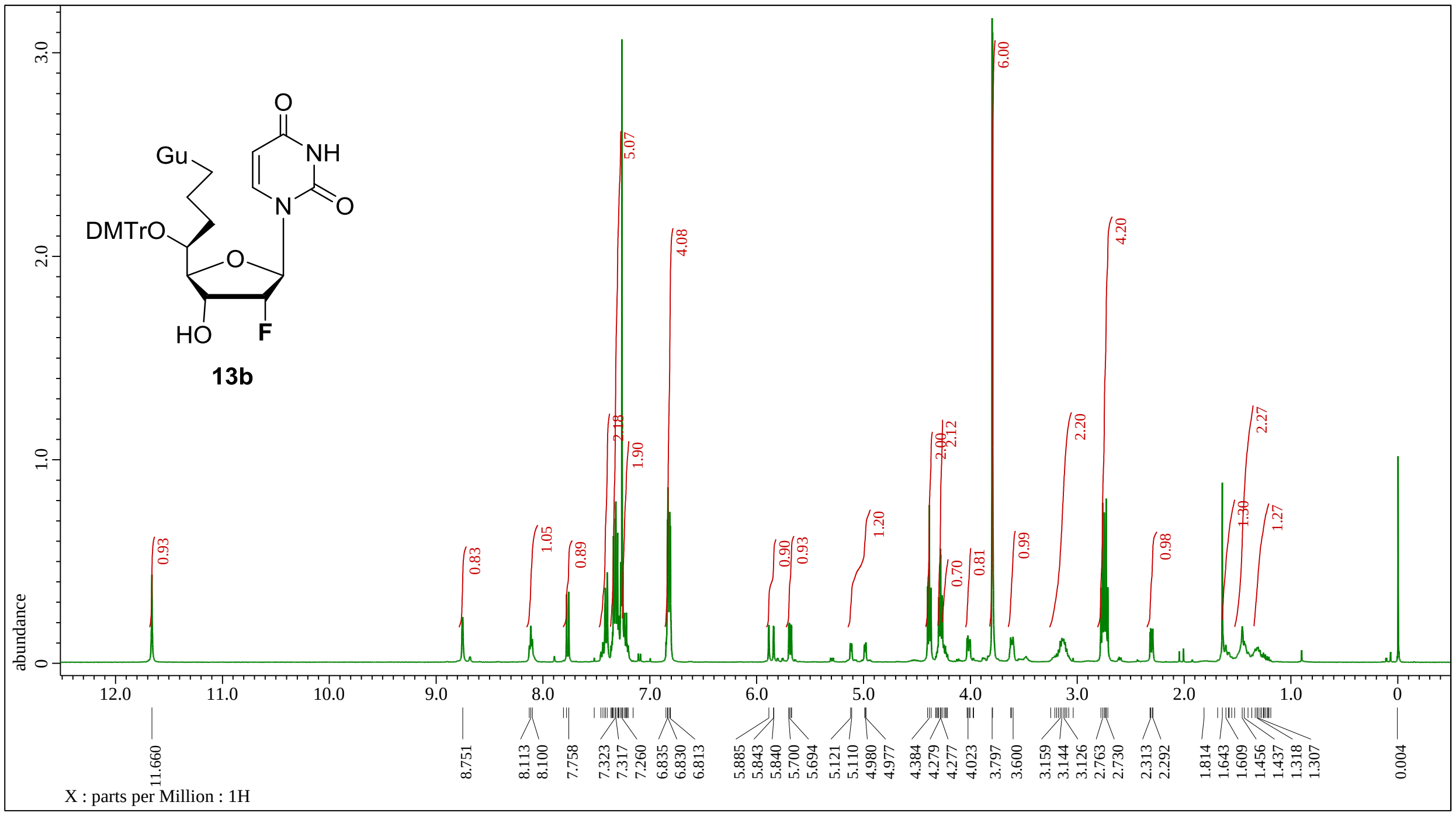

^{13}CNMR (101 MHz, $CDCl_3$) **13b**

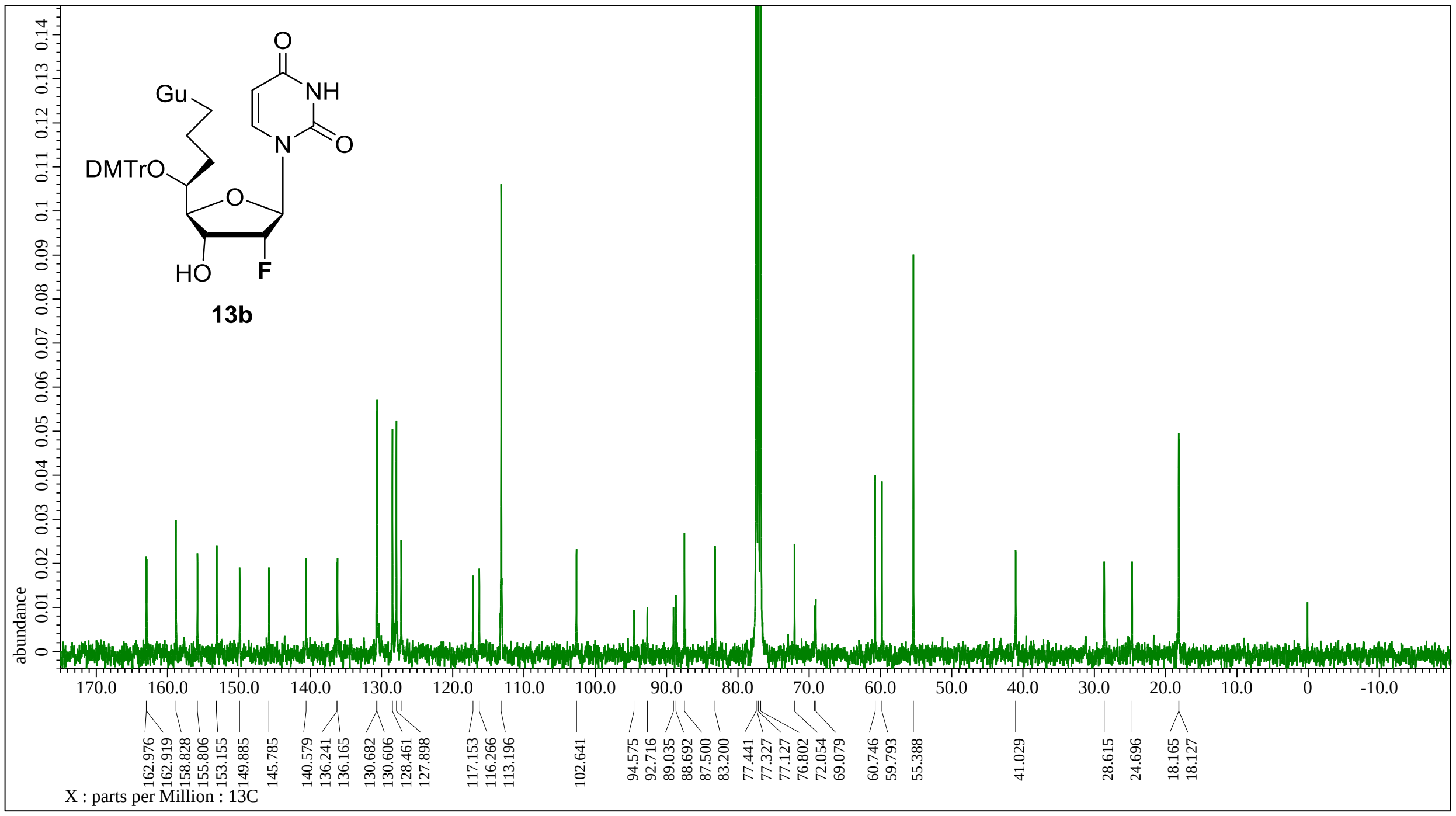

^{31}PNMR (162 MHz, $CDCl_3$) **14a**

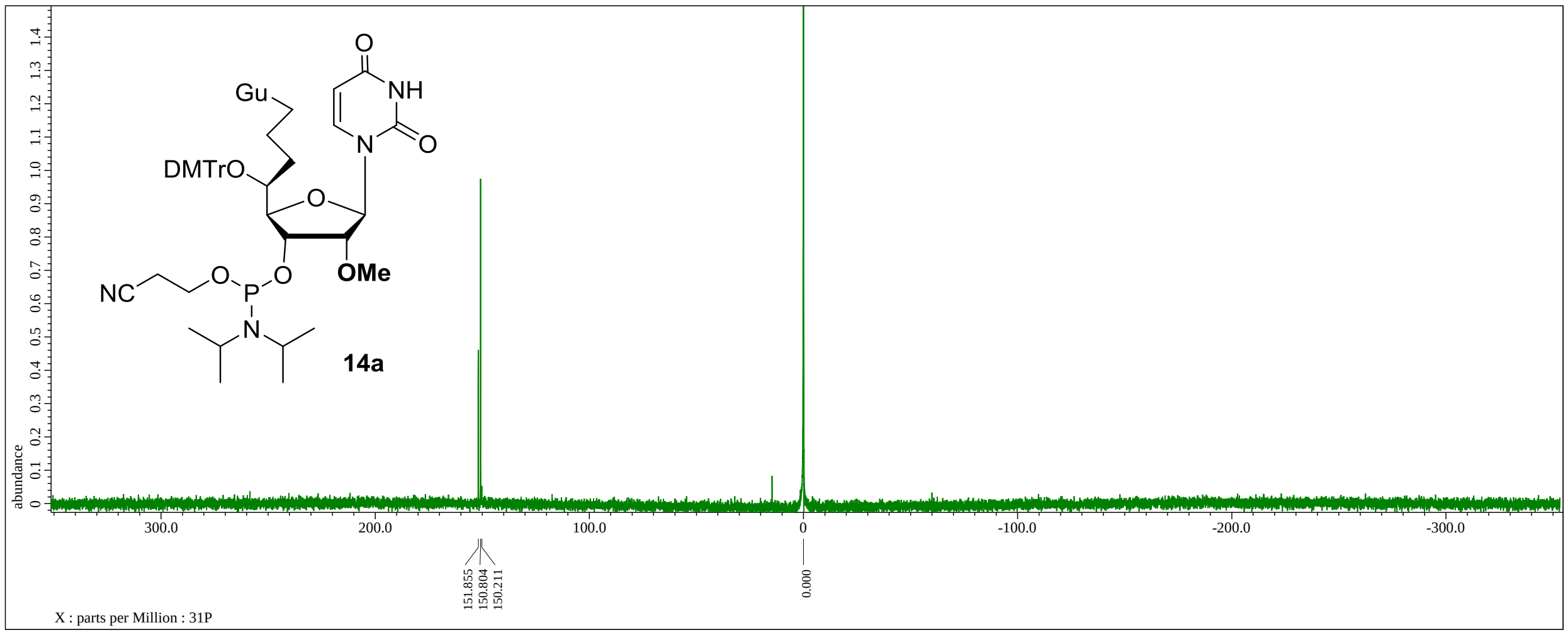

^{31}PNMR (162 MHz, $CDCl_3$) **14b**

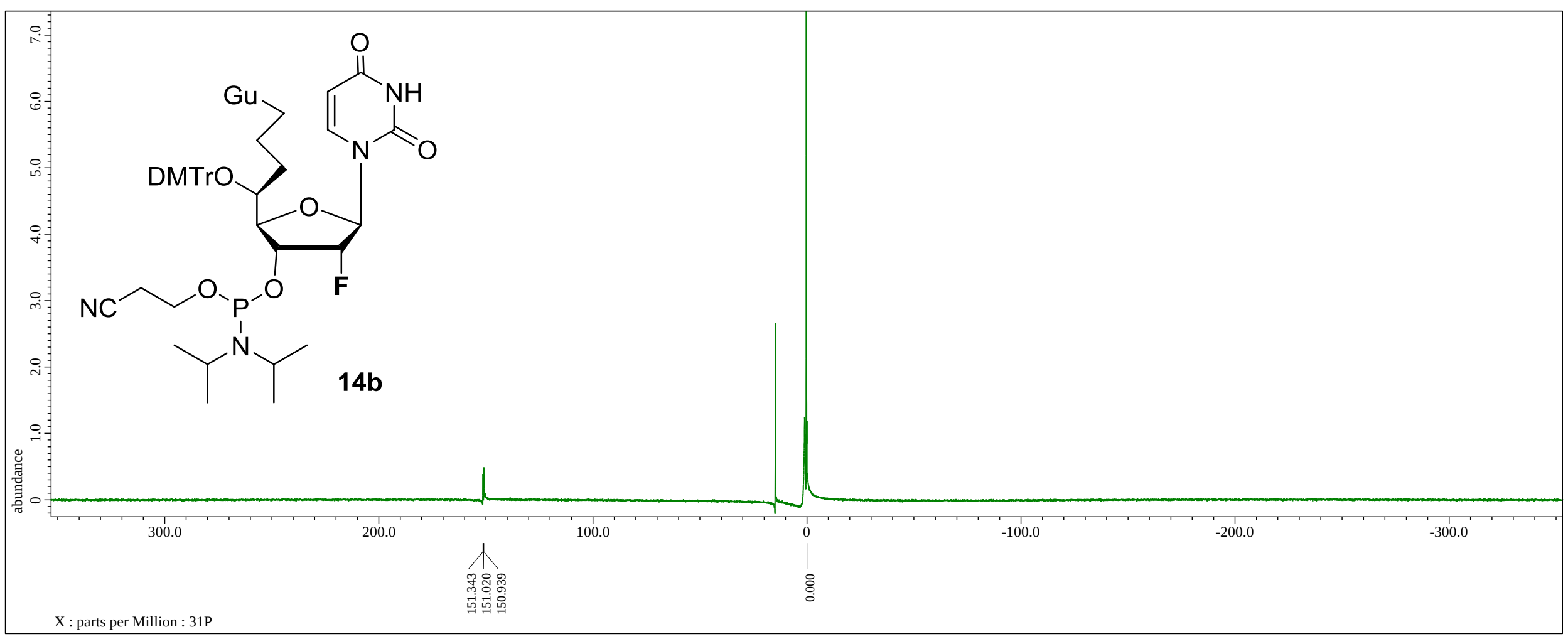

Supplement: Supplementary file 1 [file CMDC-21-e70413-s001.pdf]
